# MT1G induces lipid droplet accumulation through modulation of H3K14 trimethylation accelerating clear cell renal cell carcinoma progression

**DOI:** 10.1038/s41416-024-02747-y

**Published:** 2024-06-21

**Authors:** Sen Wang, Kexin Wang, Dong Yue, Xiaxia Yang, Xiaozao Pan, Feifei Kong, Rou Zhao, Qingli Bie, Dongxing Tian, Shuqing Zhu, Baoyu He, Zhang Bin

**Affiliations:** 1 Department of Laboratory Medicine, Affiliated Hospital of Jining Medical University, Jining Medical University, Jining, Shandong 272007 China; 2 Postdoctoral Mobile Station of Shandong University of Traditional Chinese Medicine, Jinan, Shandong 250355 China; 3 Department of Medical Imaging, Affiliated Hospital of Jining Medical University, Jining, Shandong 272007 China; 4 Department of Urology, Affiliated Hospital of Jining Medical University, Jining, Shandong Province 272007 China; 5 Department of Digestive Endoscopy, Affiliated Hospital of Jining Medical University, Jining, Shandong Province 272007 China

**Keywords:** Surgical oncology, Renal cell carcinoma

## Abstract

**Background:**

Lipid droplet formation is a prominent histological feature in clear cell renal cell carcinoma (ccRCC), but the significance and mechanisms underlying lipid droplet accumulation remain unclear.

**Methods:**

Expression and clinical significance of MT1G in ccRCC were analyzed by using TCGA data, GEO data and scRNASeq data. MT1G overexpression or knockdown ccRCC cell lines were constructed and in situ ccRCC model, lung metastasis assay, metabolomics and lipid droplets staining were performed to explore the role of MT1G on lipid droplet accumulation in ccRCC.

**Results:**

Initially, we observed low MT1G expression in ccRCC tissues, whereas high MT1G expression correlated with advanced disease stage and poorer prognosis. Elevated MT1G expression promoted ccRCC growth and metastasis both in vitro and in vivo. Mechanistically, MT1G significantly suppressed acylcarnitine levels and downstream tricarboxylic acid (TCA) cycle activity, resulting in increased fatty acid and lipid accumulation without affecting cholesterol metabolism. Notably, MT1G inhibited H3K14 trimethylation (H3K14me3) modification. Under these conditions, MT1G-mediated H3K14me3 was recruited to the CPT1B promoter through direct interaction with specific promoter regions, leading to reduced CPT1B transcription and translation.

**Conclusions:**

Our study unveils a novel mechanism of lipid droplet accumulation in ccRCC, where MT1G inhibits CPT1B expression through modulation of H3K14 trimethylation, consequently enhancing lipid droplet accumulation and promoting ccRCC progression.

**Figure:**
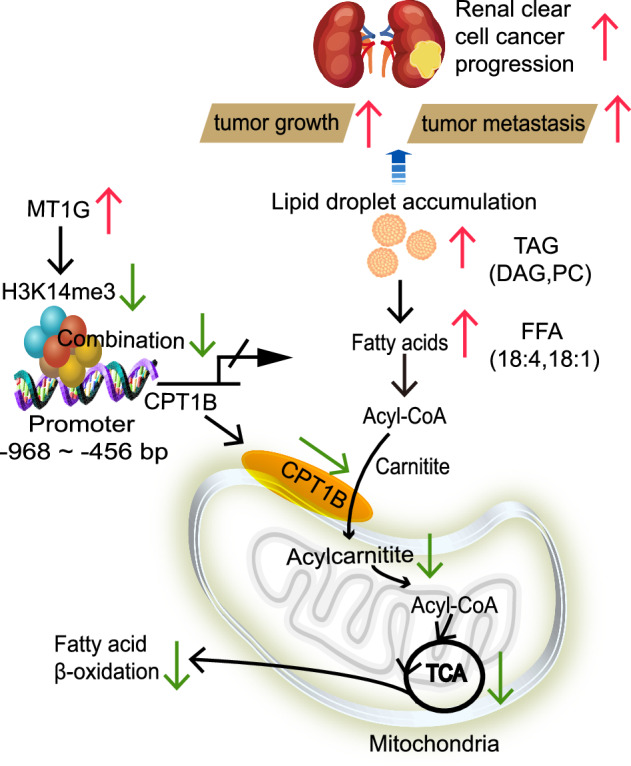
Graphical abstract figure Schematic diagram illustrating MT1G/H3K14me3/CPT1B-mediated lipid droplet accumulation promoted ccRCC progression via FAO inhibition.

## Introduction

Clear cell renal cell carcinomas (ccRCCs) constitute approximately 75% of all cases of renal cell carcinoma (RCC) and are responsible for the majority of RCC-related deaths [1]. While surgical and ablative methods are effective treatments for early-stage ccRCC, a significant fraction of patients eventually develop metastases. This underscores the urgent need for early diagnostic markers, exploration of molecular mechanisms, and the identification of effective targets to enhance survival rates among kidney cancer patients. Historically, ccRCCs are characterized by malignant renal tubular epithelial cells with a clear cytoplasm, resulting from extensive lipid and glycogen accumulation [2]. Research in various cancer types has unveiled alterations in metabolic pathways governing tumor energy production and biosynthesis, termed “metabolic reprogramming”. Studies in ccRCC have been particularly enlightening, leading to the recognition of ccRCC as a metabolic disorder. In ccRCC, metabolic reprogramming typically encompasses changes in glucose and fatty acid metabolism, along with modifications to the tricarboxylic acid (TCA) cycle [3]. Lipid droplets, the primary intracellular lipid storage sites, serve as indicators of lipid deposition in cells. Research indicates that lipid droplet accumulation in ccRCC not only contributes to membrane biosynthesis but also exerts critical control over cancer proliferation and metastasis [4]. Therefore, targeting lipid deposition represents a promising therapeutic approach and an avenue for better comprehending the intricate mechanisms at play.

Recent evidence highlights the pivotal roles of Metallothioneins (MTs), small cysteine-rich proteins, in tumor initiation, progression, and drug resistance. Furthermore, specific MT isoforms’ varying expression levels offer diagnostic and therapeutic potential [5]. Among these isoforms, MT1G, a newly identified member of the MT1 family in humans, has been linked to ferroptosis in various cancers, including colorectal cancer, hepatocellular carcinoma (HCC), esophageal adenocarcinoma, prostate cancer, pancreatic adenocarcinoma, glioblastoma, and osteosarcoma [6–12]. However, the role of MT1G and its relationship with lipid accumulation in ccRCC remain unexplored. While histone modification is known to play a crucial role in tumorigenesis and malignant progression, its association with lipid metabolism in ccRCC has received scant attention. Importantly, the interplay between MT1G, histone modification, lipid metabolism, and their collective impact on ccRCC progression remains to be elucidated.

In our study, we observed a significant decrease in MT1G expression in ccRCC cells compared to adjacent normal renal cells, making it a promising diagnostic marker to distinguish renal cancer from normal tissue. Notably, high MT1G expression correlated with poorer prognosis in ccRCC patients. In vitro experiments revealed that MT1G promoted proliferation, migration, and resistance to sormentalafenib in ccRCC cells. In vivo, MT1G overexpression accelerated renal cancer tumor growth and metastasis. Mechanistically, MT1G was found to suppress the expression of CPT1B by inhibiting the binding of H3K14m3 to the CPT1B promoter, thereby impeding lipid droplet depletion and facilitating lipid droplet accumulation, ultimately driving ccRCC progression.

## Materials and methods

A detailed description of the materials and methods used in this study is available in the online supplementary materials.

## Results

### MT1G expression as a sensitive marker for ccRCC tissue identification and its prognostic implications

**Fig. 1:**
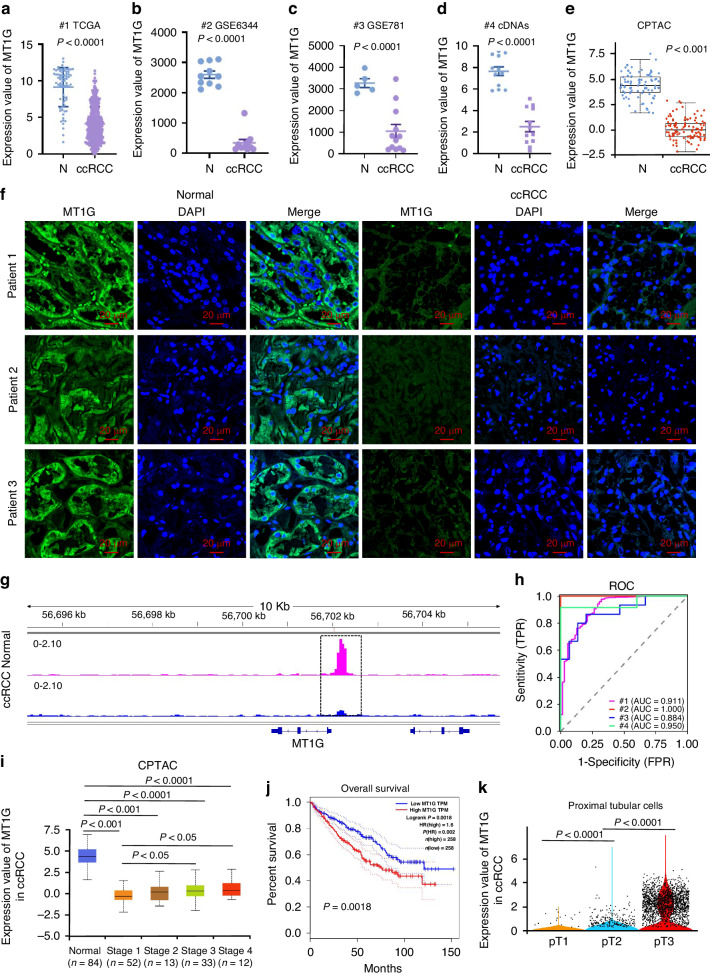
Expression, diagnostic and prognostic analysis of MT1G in ccRCC. **a** MT1G expression analysis in the TCGA KIRC cohort were analyzed in ccRCC tissue (*n* = 530) relative to normal prostate tissue (*n* = 72, unpaired *t* test, *P* < 0.0001). **b**, **c** Comparison of MT1G gene expression levels between ccRCC tissues and normal tissues in online datasets (GSE6344 (*n* = 10, ccRCC *n* = 10), paired *t* test, *P* < 0.0001 and GSE781(*n* = 5, ccRCC*n* = 12), unpaired *t* test, *P* < 0.0001). **d** Real-time PCR analysis of MT1G expression in RNA samples extracted from ccRCCand adjacent normal tissue. (*n* = 15, paired *t* test, *P* < 0.0001). **e** Evaluation of MT1Gprotein levels in matched ccRCC patient samples (*n* = 110) and adjacent normal samples (*n* = 84) by using UCLCAN online software, *P* < 0.001. **f** Fluorescent immunohistochemical staining of MT1G protein inccRCC tumor and normal tissue from the same patient from Department of Urology, Affiliated Hospital of Jining Medical College. MT1G antibody (Cusabio) ratio was 1:50. Scale bar was 20 µm. **g** ATAC-seq signal in normal kidney tissue (Normal, red track) and below ccRCC tissue (ccRCC, blue track) at the MT1G locus (bottom) with differentially accessible. **h** Receiver-operating characteristics (ROC) curve for MT1G expression in detecting ccRCC cancer and normal kidney tissue using various datasets(#1:TCGA KIRC datasets, #2:GSE6344, #3:GSE781 and #4: cDNAs) datasets. **i** Analysis of MT1Gprotein levels in matched normal and ccRCC cancerpatient samples with different pathological stages by using UCLCAN online software. **j** Analysis of MT1G expression and its correlation with survival prognosis in patients with ccRCC cancer, *p* < 0.005. **k** RCC 1 (pT1), RCC2 (pT2) and RCC 5 (pT3) patient data were downloaded from the single-cell sequencing data GSE224630 and cell subsets were classified according to the cell marker gene. MT1G gene expression in tubular epithelial cells in renal clear cell carcinoma of different stages was analyzed for statistical analysis.

Analysis of the TCGA database revealed that the MT1G gene exhibits reduced expression in ccRCC cancer (Fig. 1a). This finding was corroborated by data from the GES6344 and GSE781 databases, which also showed diminished MT1G expression in ccRCC cancer patients (Fig. 1b, c). To confirm this at the mRNA level, we collected cDNAs from both ccRCC cancer and normal adjacent tissues, performing qPCR assays that validated this observation (Fig. 1d). Moreover, the CPTAC database analysis indicated that MT1G’s protein level was lower in ccRCC cancer than in normal adjacent tissue (Fig. 1e). Immunofluorescence results further supported a decrease in MT1G protein expression in ccRCC cancer patients (Fig. 1f, Supplementary Fig. 1a). ATAC-seq data indicated reduced chromatin openness in ccRCC cancer tissues compared to normal tissues (Supplementary Fig. 1b). Notably, among these genes, MT1G emerged as a crucial player with diminished chromatin openness in ccRCC (Fig. 1g). The ROC curve demonstrated that MT1G mRNA expression in the aforementioned datasets (TCGA, GSE6344, GSE781, and cDNAs) exhibited high clinical diagnostic value (AUC = 0.911, 1.000, 0.884, 0.950) (Fig. 1h). Interestingly, we observed a positive correlation between high MT1G expression and the malignant progression stage of ccRCC cells (Fig. 1i), while it did not impede MT1G’s utility as a ccRCC tissue indicator. Prognostic analysis indicated that up-regulation of MT1G was linked to poor prognosis and lower disease-free survival rates for ccRCC patients (Fig. 1j and Supplementary Fig. 1c, d). *T* tests revealed that high MT1G expression was more prevalent in advanced ccRCC patients over 60 years old, as well as in patients with higher pathological grades (*P* < 0.05), higher clinical stages (III-IV stage) (*P* < 0.05), T3-4 depth (*P* < 0.05), and deceased patients (Supplementary Fig. 1e–k). Chi-square tests showed a significant positive association between high MT1G expression and patient age and histologic grade (*P* < 0.05) (Table 1). Single-cell sequencing data indicated that MT1G was primarily expressed in proximal tubule cells (Supplementary Fig. 1l, m, n), which are the presumptive origin of ccRCC cells [13]. We further analyzed the expression of MT1G in renal clear cell carcinoma tubular epithelial cellswith different malignant progression stages in GSE224630 single cell data, and found that the expression of MT1G in tubular epithelial cells increased with increasing malignant stage (Fig. 1k).These findings suggest that MT1G may play a tumor-promoting role during ccRCC progression.

**Table 1:** Relationship between MT1G expression and clinicopathological parameters in KIRC patient.

Clinicopathological parameters	Patient type	Expression level of MT1G ^a^		
		High Number (n) Low Number (n)		*P value*
age	>60	119	144	0.04494*
	<60	142	121	
stage	1	127	136	0.0984
	2	22	34	
	3	64	58	
	4	49	33	
histologic_grade	G1	6	8	0.02055*
	G2	105	119	
	G3	100	105	
	G4	50	25	
M	M0	203	213	0.1094
	M1	46	32	
N	N0	118	121	1
	N1	8	8	
T	T1	130	139	0.3674
	T2	30	38	
	T3	96	82	
	T4	7	4	
gender	female	82	102	0.08223
	male	181	161	

^a^The high-level and low-level groups was determined by the average score of MT1G. *P* < 0.05 represents significant statistical significance.

### MT1G promotes ccRCC tumor cell growth and metastasis in vitro and in vivo

To investigate the role of MT1G in ccRCC cancer development, we conducted cell function experiments using ccRCC 786-O and A498 cell lines. Lentiviruses carrying MT1G overexpression and knockdown constructs were successfully introduced into these cells, resulting in altered MT1G expression levels, both at the mRNA and protein levels (Supplementary Fig. 2a, b). Cell proliferation assays revealed that MT1G overexpression promoted the proliferation of 786-O and A498 cells compared to control cells (Supplementary Fig. 3a, b), whereas MT1G knockdown suppressed cell viability in both ccRCC cell lines (Supplementary Fig. 3c, d). Flow cytometry analysis demonstrated that MT1G overexpression reduced the number of cells in the G1 phase while increasing those in the S phase for 786-O cells (Supplementary Fig. 3e). In A498 cells, MT1G overexpression decreased the number of G1 phase cells and increased those in the G2/M phase (Supplementary Fig. 3f). Conversely, MT1G knockdown led to opposite effects in both cell lines (Supplementary Fig. 3g, h). These alterations in cell cycle progression correlated with changes in the expression of cyclin kinases CDK 2, CDK 4, CDK 6, CyclinD1, and the cell cycle inhibitory gene P21 (Supplementary Fig. 2c). Apoptosis assays using flow cytometry showed that MT1G had no significant effect on apoptosis in 786-O and A498 cells (Supplementary Fig. 2d, e). Sorafenib resistance experiments demonstrated that MT1G overexpression significantly reduced the sensitivity of ccRCC cells to sorafenib, while MT1G knockdown enhanced sensitivity (Supplementary Fig. 3i-j). Moreover, qPCR and western blot assays revealed that sorafenib could concentration-dependently induce the expression of MT1G at both the mRNA and protein levels (Supplementary Fig. 3k, l). In cell migration assay using transwell chambers, MT1G overexpression promoted the migration of ccRCC cells relative to control cells, while MT1G knockdown reversed this effect (Supplementary Fig. 3m–p). Additionally, high MT1G expression led to alterations in migration-associated marker proteins, including the inhibition of E-cadherin and increased expression of N-cadherin and Vimentin, both at the mRNA (Supplementary Fig. 2f, g) and protein levels (Supplementary Fig. 3q).

**Fig. 2:**
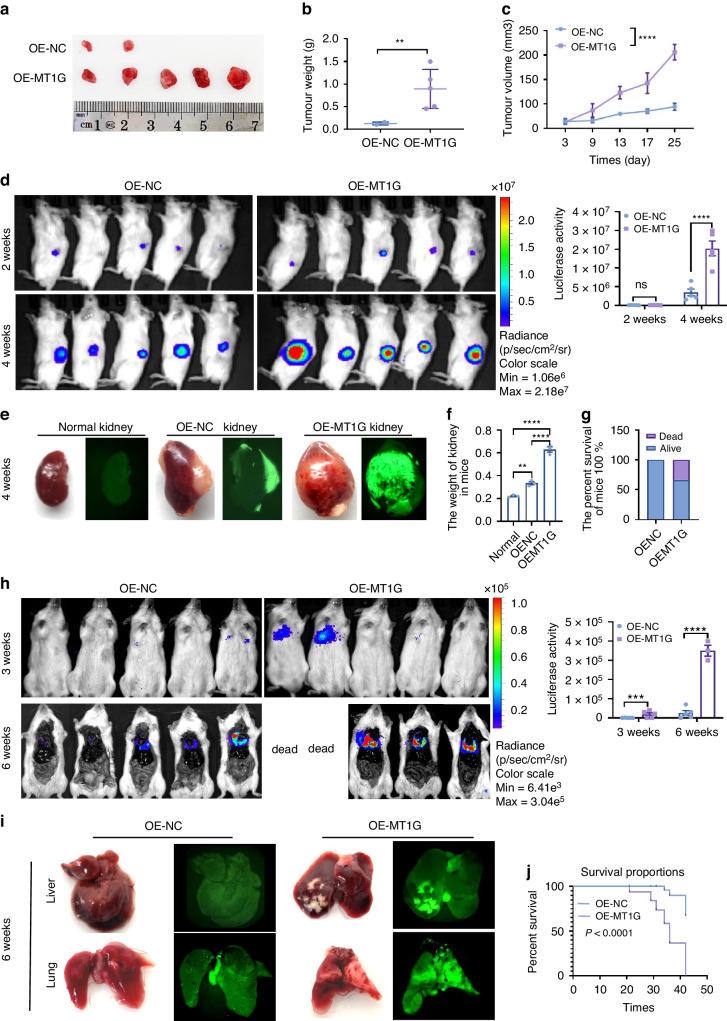
Analysis of MT1G-regulated tumor growth and metastasis in ccRCC models in vivo. **a** Subcutaneous injection of 786-O cells stably overexpressing MT1G or control virus into BALB/c nude mice (*n* = 5). Subsequently, xenograft tumors were dissected and representative images were obtained at day 26 post-injection. **b** Statistical analysis of tumor weights after dissection. **c** Tumor growth curve during the inoculation period. Data are presented as the mean ± SD, *n* = 5. ****p < 0.0001, ***P < 0.001, **P < 0.01, and *P < 0.05 as determined by the *T* test. **d** Live imaging analysis of the effect of MT1G expression on renal clear cell cancer growth in situ. 5 × 10^6 786-O cells stably overexpressing MT1G or control virus were subcutaneously injected into NVSG. After 2 weeks, tumors were dissected out and 1 mm3 of tumors from two groups were injected into the kidney in situ(*n* = 7), five mice were randomly selected from each group for live imaging, and kidney tumor size in mice were quantified at weeks 2 and 4 (*n* = 5). Fluorescence intensity in situ of mice were compared between OENC and OEMT1G group mice, data were presented as the mean ± SD, *n* = 5. ****P < 0.0001, ***P < 0.001, **P < 0.01, and *P < 0.05 as determined by the *T* test. **e** GFP signals analysis of OEMT1G and OENC orthotopic clear cell renal carcinoma mice by using the Axio Zoom V16 luciferase imaging system (ZEISS). Three of OEMT1G mice that were preferentially dead, three of OENC mice were randomly sacrificed as control mice, and kidneys from above mice were harvested and Axio Zoom V16 luciferase imaging based on GFP were performed. Representative fluorescent pictures of the kidney in OEMT1G group and OENC group orthotopic renal carcinoma mice at week 4 were shown. **f** Analysis of mouse orthotopic renal tumor weight in OEMT1G group and OENC group mice at week 4. The normal right kidney of the OENC group was used as a normal reference control. Three mice of OEMT1G group and OENC group cells ulated orthotopic renal tumor has been compared using *t* test (*n* = 3). **g** Survival ratio analysis in OEMT1G and OENC orthotopic clear cell renal carcinoma mice. The mice in the OENC group did not die in the fourth week, and the OEMT1G group began to die in the fourth week, and 7 died 3. **h** Live imaging analysis of the effect of MT1G expression on renal clear cell cancer tumor metastasis. 2 × 10^6 renal clear carcinoma cell carcinoma 786-O cells containing luciferase were injected into NVSG mice through tail vein, live imaging of mice were shown at week 3 and 6, *n* = 5 or 3. Fluorescence intensity in NVSG mice were compared between OENC and OEMT1G group orthotopic clear cell renal carcinoma mice, data were presented as the mean ± SD, *n* = 5 or 3. ****P < 0.0001, ***P < 0.001, **P < 0.01, and *P < 0.05 as determined by the *T* test. **i** GFP signals analysis in OEMT1G and OENC tail vein metastases mice by using the Axio Zoom V16 luciferase imaging system (ZEISS). One of OEMT1G mice and one of OENC mice were randomly sacrificed, and the lung and liver were harvested for Axio Zoom V16 luciferase imaging based on GFP were performed. **j** Survival analysis of OEMT1G and OENC tail vein metastases mice. 2 × 10^6 renal clear carcinoma cell carcinoma 786-O cells were injected into NVSG mice through tail vein (*n* = 7), survival curves were statistically analyzed during the cultivation period.

To assess whether MT1G promotes ccRCC cell growth in vivo, we subcutaneously injected ccRCC 786-O cells into four-week-old male nude mice. After 26 days, mice were euthanized and tumor tissues were removed and weighed. During these 26 days, the tumor volumes were measured and immunohistochemistry (IHC) analysis of tumor tissues using MT1G, Ki67, E-cadherin, and N-cadherin antibodies were performed. Above results revealed that MT1G overexpression increased the subcutaneous tumorigenic capacity of ccRCC 786-O cells compared to the control group, resulting in more tumors (Fig. 2a), increased tumor weight (Fig. 2b), and larger tumor volumes (Fig. 2c). Hematoxylin-eosin (HE) staining and IHC results showed that MT1G overexpression correlated with increased expression of Ki67, N-cadherin, and Vimentin, as well as decreased E-cadherin expression in xenograft tumor cells (Supplementary Fig. 4a). Equal weight of subcutaneous tumor from MT1G-overexpressing cells and control cells carrying a luciferase/GFP reporter gene were injected into right kidney of NVSG mice, and the effect of MT1G expression on the growth of renal orthotopic tumor was analyzed by live imaging, and these results showed that MT1G overexpression promoted the renal clear cell carcinoma growth in situ (Fig. 2d). Kidney GFP signals and kidney weight in OEMT1G orthotopic clear cell renal carcinoma mice were much more than OENC group mice (Fig. 2e, f). Analysis of survival ratio showed that renal tumors mice in situ with MT1G overexpression died more than control OENC group (Fig. 2g). Moreover, a detectable liver metastatic nodules was found in orthotopic renal carcinoma model mice with MT1G overexpression, whereas it was not be found in control group (Supplementary Fig. 4b). Furthermore, we injected MT1G-overexpressing cells and control cells carrying a luciferase/GFP reporter gene into the tail veins of severely immunodeficient NVSG mice. After six weeks, we observed liver and lung metastases using live imaging. MT1G-overexpressing 786-O cells developed more detectable liver and lung metastases than the control group (Fig. 2h, i; Supplementary Fig. 4c). Additionally, we conducted tail vein metastasis experiments in four-week-old male nude mice to confirm the role of MT1G in promoting lung and liver metastasis. Although nude mice are only deficient in T lymphocytes, resulting in a low metastasis rate compared to NVSG mice, IVIS imaging revealed that MT1G also promoted the formation of liver and lung metastatic nodules compared to the respective control groups (Supplementary Fig. 4d). Survival curve analysis of tail vein transfer model mice showed that 786-O cells expressing high MT1G significantly inhibited viability compared with control group mice (Fig. 2j).

### MT1G modulates fatty acid metabolism by downregulating fatty acyl carnitine expression in ccRCC cells

To elucidate the potential mechanisms underlying MT1G’s role in human ccRCC progression, we conducted GSEA to explore signaling pathways from the KEGG database in samples with high and low MT1G expression. Our analysis of KEGG terms revealed a significant association between MT1G expression and fatty acid metabolism (Normalized Enrichment Score [NES] = –2.41, *p* = 0.00, *q* = 0.00) (Table 2). Moreover, GSEA data indicated that genes related to fatty acid metabolism were predominantly enriched in the low MT1G expression group (Supplementary Fig. 5a). Notably, analysis of the UCLAN database demonstrated a correlation between MT1G expression in ccRCC tissue and the obesity level of ccRCC patients (Supplementary Fig. 5b). Differential gene enrichment analysis of transcriptomic data following MT1G knockdown revealed significant impacts on genes associated with metabolic pathways, including the sterol signaling pathway, cholesterol metabolism, fatty acid metabolism, glucose and lipid metabolism, among others (Supplementary Fig. 5c). ATAC-seq results also showed that MT1G significantly influenced differential open genes enriched in metabolic pathways, such as the sphingolipid signaling pathway and glucose and lipid metabolism (Supplementary Fig. 5d).

**Table 2:** The expression of MT1G gene in tumor was analyzed by GSEA.

ID^a^	Set Size^b^	ES^c^	NES^d^	*P* value	*P*. adjust^e^	*Q* values^f^
**KEGG_FATTY_ACID_METABOLISM**	**17**	**−0.62**	**−2.41**	**0.00**	**0.00**	**0.00**
KEGG_PROPANOATE_METABOLISM	14	−0.52	−1.91	0.01	0.08	0.07
KEGG_PPAR_SIGNALING_PATHWAY	32	−0.37	−1.87	0.01	0.09	0.07
KEGG_PHOSPHATIDYLINOSITOL_SIGNALING	29	−0.33	−1.57	0.02	0.20	0.17
KEGG_GLYCEROLIPID_METABOLISM	17	−0.36	−1.40	0.08	0.33	0.28
KEGG_ADIPOCYTOKINE_SIGNALING_PATHWAY	24	−0.30	−1.38	0.12	0.39	0.33
KEGG_GLYCEROPHOSPHOLIPID_METABOLISM	29	−0.24	−1.17	0.26	0.61	0.51

^a^Is the name of the gene set.

^b^Represents the total number of genes in the gene set.

^c^Represents enrichment score.

^d^Represents the normalized enrichment score.

^e^Is a function used to adjust the p value, which can help to control the false positive error rate.

^f^Is the p value corrected for multiple hypothesis testing.

The high-level and low-level groups were determined by the average score of MT1G. *P* < 0.05 represents significant statistical significance.

**Fig. 3:**
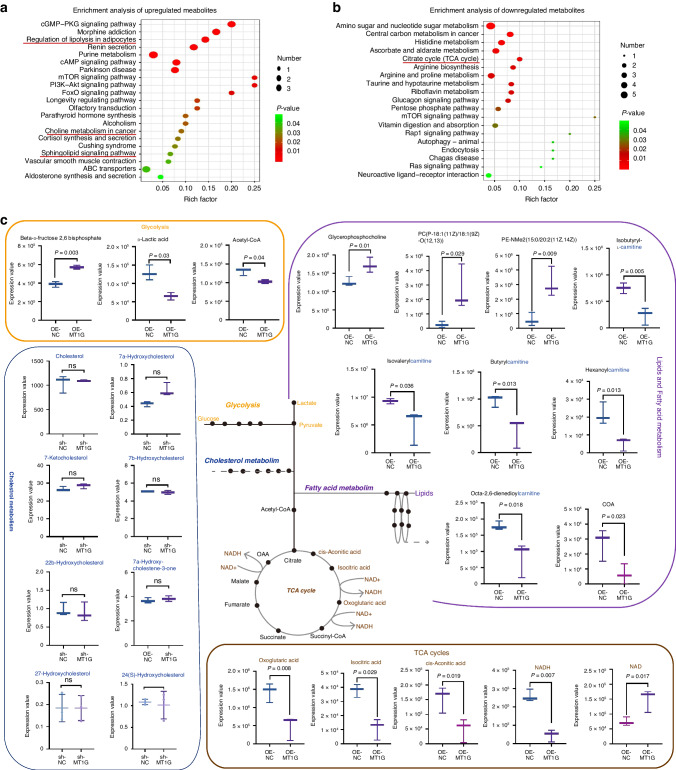
Metabolomic impact of MT1G regulation. **a**, **b** KEGG analysis of the upregulated and downregulated metabolites induced by MT1G. The significance of metabolic pathway enrichment was indicated by the *p* value. Enriched pathways were shown in the bubble plot. Metabolic pathway names were on the vertical axis, while the horizontal axis represented the enrichment factor (Rich factor=Number of significantly differential metabolites/Total metabolites in this pathway). A larger rich factor indicates greater enrichment. Colors transition from green to red, signifying decreasing *p* values. Larger bubbles represent higher metabolite enrichment. **c** Classification of significantly altered metabolites influenced by MT1G across glycolysis, lipid, fatty acid metabolism, cholesterol metabolism, and the TCA cycle pathways. Quantitative data was represented as fold change (FC) + SD relative to control (shNC or OENC). Statistically significant changes are denoted by *P* < 0.05.

To explore metabolic alterations influenced by MT1G expression, we conducted non-targeted metabolomics analysis in 786-O cells, followed by LC-MS analysis of cell lysates. We identified 62 up-regulated and 75 down-regulated metabolites affected by MT1G overexpression (*P* < 0.05) (Supplementary Fig. 5e). The variable weight values (VIP) were used to assess the influence and explanatory power of each metabolite’s expression pattern on group classification discrimination (VIP > 1, *P* < 0.05). Among the top three metabolites with the highest VIP scores, isovalerylcarnitine (VIP = 13.22, log 2(OEMT1G/OENC)= –0.46, *P* = 0.005), PE-NME2(15:0/20:2(11Z,14Z)) (VIP = 12.69, log 2(OEMT1G/OENC) = 2.483, *P* = 0.046), and PC(P-18:1(11Z)/18:1(9Z)-O(12,13)) (VIP = 10.147, log2(OEMT1G/OENC) = 2.785, *P* = 0.005) stood out (Supplementary Fig. 5f). Notably, isovalerylcarnitine, isobutyrylcarnitine, butyrylcarnitine, and oxoglutaric acid were significantly decreased, while glycerophosphocholine, PC(P-18:1(11Z)/18:1(9Z)-O(12,13)), and PE-NMe2(15:0/20:2(11Z,14Z)) were upregulated in the MT1G-higher expression group among the top 20 VIP score metabolites (Supplementary Fig. 5f). Further analysis of differential metabolites using KEGG pathways revealed that upregulated metabolites were associated with the regulation of lipolysis in adipocytes and the sphingolipid signaling pathway, among others, while downregulated metabolites were enriched in the TCA cycle (Fig. 3a, b). We classified remarkably differential metabolites related to glycolysis, lipid and fatty acid metabolism, cholesterol metabolism, and the TCA cycle pathway (Fig. 3c). Our results demonstrated that MT1G downregulated the TCA cycle and glycolysis processes, as well as fatty acyl carnitines, while upregulating lipid-related metabolites (Fig. 3c). Notably, targeted cholesterol metabolomics analysis showed that MT1G had no effect on cholesterol metabolism (Fig. 3c). Pearson correlation analysis revealed strong negative correlations among differential metabolites. Notably, glycerophospholipid metabolites (PC(P-18:1(11Z)/18:1(9Z)-O(12,13)), PE-NME2(15:0/20:2(11Z,14Z)), glycerophosphocholine) and carnitine metabolites (isobutyryl-L-carnitine, butyrylcarnitine, and isovalerylcarnitine) exhibited significant negative correlations (*P* < 0.05, correlation > 0.95) (Supplementary Fig. 5g, h). Carnitine and acylcarnitines, such as isobutyrylcarnitine, isovalerylcarnitine, and butyrylcarnitine, were identified as biomarkers related to fatty acid metabolism [14]. These findings suggest that MT1G inhibits the TCA cycle and glycolysis processes while suppressing fatty acid metabolism and upregulating lipid levels.

### MT1G upregulates lipid metabolites and induces lipid droplet accumulation in ccRCC cells thereby accelerating ccRCC development

**Fig. 4:**
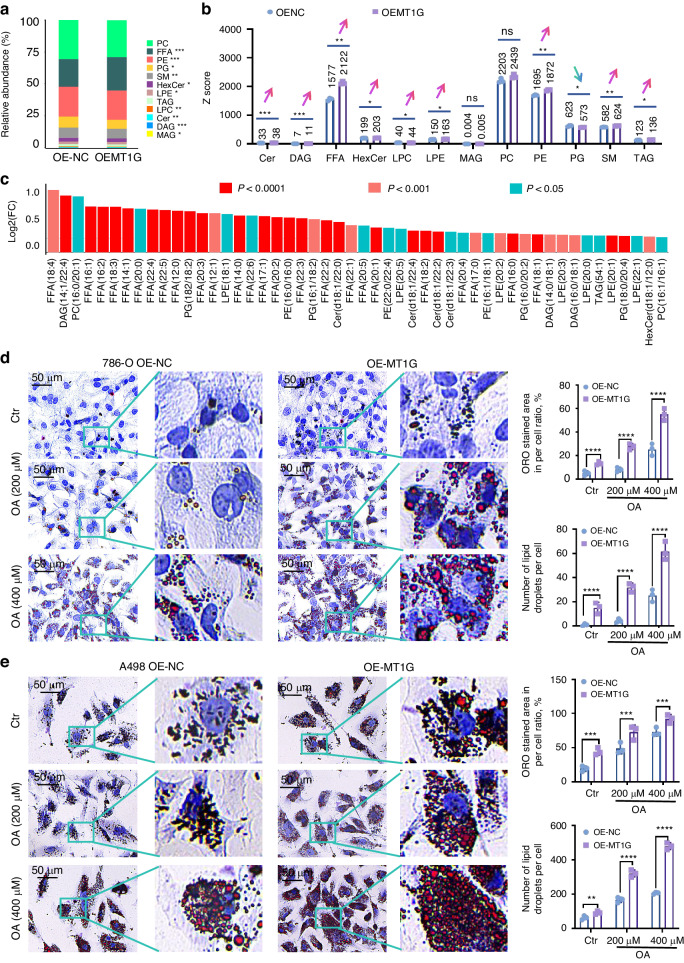
Analyses of targeted lipid metabolomics and lipid droplets regulated by MT1G. **a** Stacked bar chart shows the relative abundance of metabolite classes in different groups. Significance levels are indicated as ****P < 0.0001, ***P < 0.001, **P < 0.01, and *P < 0.05, determined by the *T* test. **b** Z Score column plot categorizes differentially expressed metabolites into 12 lipid types. Significance levels are depicted as ****P < 0.0001, ***P < 0.001, **P < 0.01, and *P < 0.05, determined by the *T* test. **c** Analysis of fold changes and *P* values for the top 50 VIP-scored differential metabolites. The vertical axis represents log2 (FC) values, while the horizontal axis represents metabolic products. Significance is indicated as ****P < 0.0001, ***P < 0.001, **P < 0.01, and *P < 0.05, determined by the *T* test. **d** Detection and quantification of lipid droplets using ORO staining. 786-O cells with stable MT1G overexpression and control cells were subjected to various treatments, and oil droplets were quantified using Image J software.****P < 0.0001, ***P < 0.001, **P < 0.01, and *P < 0.05, determined by the *T* test.

To investigate the impact of MT1G on fatty acid metabolism, we conducted targeted lipidomics, which revealed alterations in 308 lipid molecules, with 136 being up-regulated and 16 down-regulated following MT1G overexpression (Supplementary Fig. 6a). A pie plot illustrating the proportion of identified metabolite classes across all samples demonstrated that the majority of lipids detected in renal cancer cells were phosphatidylcholines (PC), free fatty acids (FFA), and lysophosphatidylcholines (LPC) (Supplementary Fig. 6b). A stacked bar chart displaying the relative abundance of each metabolite class in different groups showed that MT1G significantly modified the lipid composition ratios, particularly those of fatty acids (Fig. 4a). Further statistical analysis identified ten types of lipids and fatty acids that were significantly up-regulated following MT1G overexpression (Fig. 4b). We ranked the top 50 significantly differential metabolites based on the fold difference, and results indicated that the fatty acid with the most substantial fold change after MT1G overexpression was FFA (18:4), while the lipid with the most pronounced difference was diacylglycerol (DAG) (14:1/22:4) (Fig. 4c). Pathway enrichment analysis, using pathway-associated metabolite sets from the Small Molecule Pathway Database (SMPDB), revealed that MT1G influenced metabolites primarily enriched in long and medium-chain fatty acid beta-oxidation and fatty metabolism, consistent with our earlier findings (Supplementary Fig. 6c). We performed ORO staining to investigate whether lipid droplets accumulated in renal cancer cells due to MT1G expression. ORO staining revealed a significant increase in lipid droplet intensity, including both the ORO staining area and the number of lipid droplets per cell, in MT1G-overexpressing 786-O and A498 cells compared to the control groups (Fig. 4d, e). Furthermore, cells with high MT1G expression exhibited significantly inhibited lipid utilization, as evidenced by increased lipid droplets compared to controls when treated with 200 or 400 μmol oleic acid (Fig. 4d, e).The impact of MT1G knockdown on lipid droplet accumulation and the growth of ccRCC were also performed, the resultsdemonstrate that MT1G knockdown significantly reduces lipid droplet accumulation in ccRCC cells when treated with 200 μmol oleic acid (Supplementary Fig. 6d, e). Moreover, our subcutaneous tumor experiment in immunodeficiency mice revealed that MT1G knockdown markedly inhibits ccRCC growth, with a noticeable decrease in the number of lipid droplets in tumors formed by MT1G knockdown cells compared to the control group (Supplementary Fig. 6f–h). These findings collectively suggest that MT1G promotes lipid accumulation in ccRCC cells thereby accelerating ccRCC development.

### MT1G suppresses fatty acid β-oxidation by inhibiting CPT1B, promoting lipid droplet accumulation

**Fig. 5:**
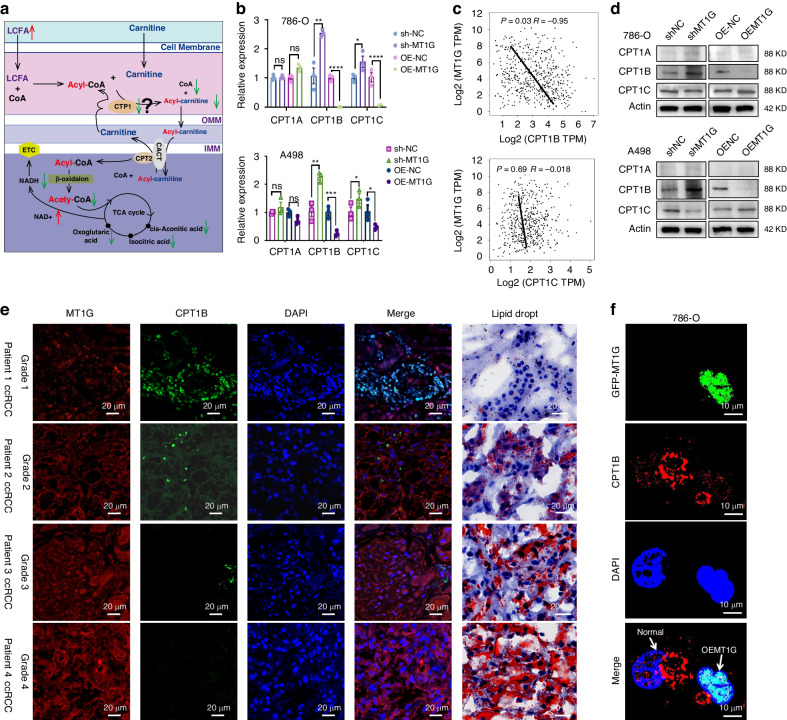
Association analysis between MT1G and CPT1B. **a** Mechanism diagram and analysis of differential metabolites involved in the transport of long-chain fatty acids into mitochondria for fatty acid oxidation. **b** qPCR analysis of CPT1B mRNA levels following MT1G overexpression or knockdown in both 786-O cells and A498 cells. **c** Western blot analysis of CPT1B protein levels after MT1G overexpression or knockdown. **d** Correlation analysis of CPT1B and MT1G using GEPIA. **e** Immunofluorescence experiments to examine the expression of CPT1B and MT1G in different grades of ccRCC patient tissues, alongside oil droplet detection. **f** Cells infected with MT1G-GFP virus (OEMT1G) and control cells not infected MT1G-GFP virus (Normal) were subjected to immunofluorescence staining experiments to examine the expression of CPT1B in clear cell renal carcinoma cells 786-o.

**Fig. 6:**
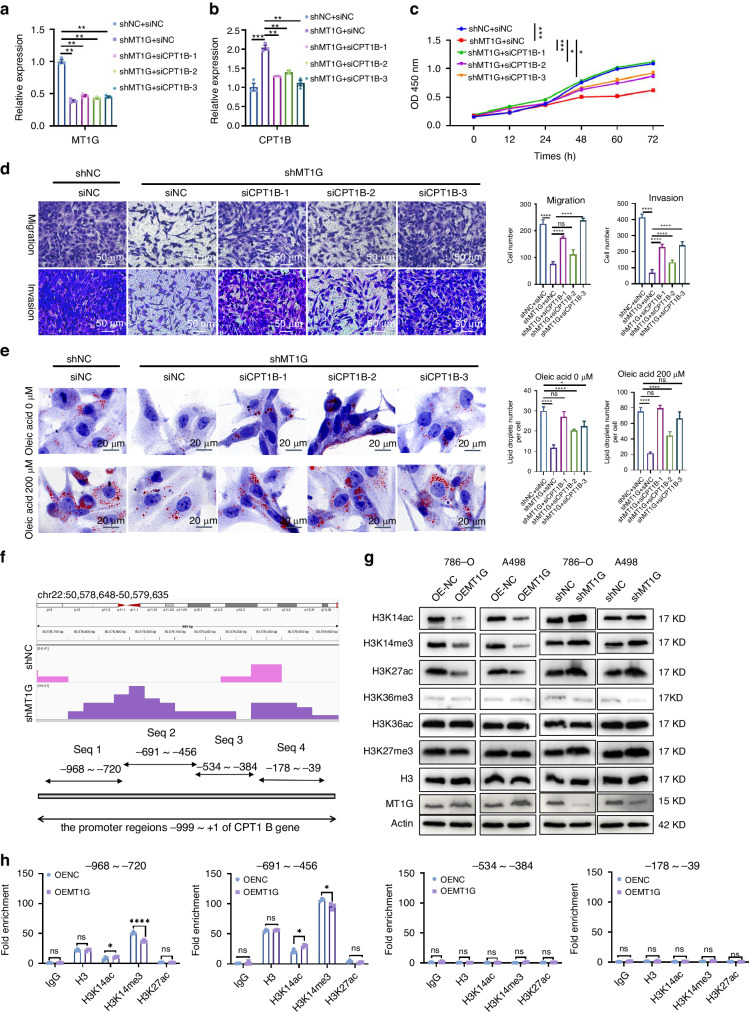
Analysis of cell proliferation, migration, and altered lipid droplet accumulation due to CPT1B knockdown in shMT1G cells and histone modifications at the CPT1B promoter. **a**, **b** Analysis of CPT1B knockdown efficiency after MT1G knockdown by qPCR in 786-O shMT1G cells. Three siRNAs were transfected, and transfection efficiency was measured by qPCR. **c** Cell proliferation assay was performed after CPT1B knockdown in shMT1G 786-O cells (*n* = 5). ****P < 0.0001, ***P < 0.001, **P < 0.01, and *P < 0.05, determined by the *T* test. **d** Migration assay was performed after CPT1B knockdown in shMT1G 786-O cells. ****P < 0.0001, ***P < 0.001, **P < 0.01, and *P < 0.05, determined by the *T* test. **e** Analysis of lipid droplet accumulation by ORO staining after CPT1B knockdown in shMT1G 786-O cells. ****P < 0.0001, ***P < 0.001, **P < 0.01, and *P < 0.05, determined by the *T* test. **f** Assessment of CPT1B promoter openness in shMT1G and shNC cells using ATAC-seq. Peaks in the CPT1B promoter region (chr22:50,578,648-50,579,635) are shown in the IGV viewer, with four primer sequences underlined for ChIP-qPCR. **g** Western blot analysis of histone modifications (H3K36me3, H3K36ac, H3K14me3, H3K14ac, H3K27me3, and H3K27ac) and histone H3 in MT1G knockdown 786-O cells, MT1G-overexpressed 786-O cells, and related control 786-O cells. Histone H3 serves as the internal control. **h** ChIP-qPCR analysis of whether histone H3K14me3, H3K14ac, and H3K27 specific antibodies bind to the indicated CPT1B promoter regions.

Mitochondrial fatty acid β-oxidation is the major pathway for the catabolism of fatty acids, the transfer of fatty acids into the mitochondria for fatty acid oxidation (FAO) need carnitine acetyltransferases which can catalyze the reversible transfer of acyl groups between coenzyme A (CoA) and carnitine, converting acyl-CoA esters into acyl-carnitine esters and vice versa [4]. This exchange step between CoA and carnitine was essential as the mitochondrial inner membrane was impermeable to long-chain CoA fatty acids [15]. The carnitine palmitoyltransferase system is responsible for delivering the long-chain fatty acid from cytoplasm into mitochondria for oxidation, where carnitine palmitoyltransferase I (CPTI) catalyzes the rate-limiting step of fatty acid oxidation (FAO), resulting in intracellular lipid accumulation. The CPT1 family of proteins contains 3 isoforms: CPT1A, CPT1B and CPT1C [16, 17]. Non-targeted metabolomics and lipid-targeted metabolomics combined analysis showed that long-chain fatty acids increased, while downstream acylcarnitines and COA decreased, and the TCA cycle decreased after MT1G overexpression (Fig. 5a). Therefore, we hypothesized that MT1G may inhibit the expression of CPTs, thus inhibiting the metabolism of fatty acids, leading to the accumulation of lipid droplets. The relative mRNA levels of CPT1B and CPT1C were significantly lower in MT1G over-expressing ccRCC cells (Fig. 5b), whereas the CPT1A mRNA levels remained unchanged (Fig. 5b). Correlation analysis showed MT1G also has a negative correlation with CPT1B (R = −0.095, *P* < 0.05) but bot CPT1C (R = −0.018, *P* = 0.69) in TCGA KIRC dataset (Fig. 5c). In addition, CPT1B were also decreased at protein levels without CPT1A and CPT1C protein levels trend changing (Fig. 5d). Polychromatic immunofluorescence experiments further demonstrated a negative relationship between MT1G and CPT1B at the protein level in different grades of ccRCC patient tissues. As MT1G levels increased, CPT1B levels progressively decreased in different grades of ccRCC cancer patients, accompanied by lipid droplet accumulation (Fig. 5e, Supplementary Fig. 7a). 786-O cells were lentivirally infected with GFP/MT1G constructs, with GFP-positive cells confirming successful infection. MT1G overexpression in these cells led to a notable decrease in CPT1B expression (Fig. 5f). Furthermore, transfection of pcdna3.0-MT1G suppressed CPT1B expression, while si-RNA-mediated MT1G knockdown inhibited CPT1B expression as observed in polyolor immunofluorescence experiments (Supplementary Fig. 7b).To confirm that MT1G inhibited lipid droplet accumulation through CPT1B, we designed three pairs of CPT1B siRNAs and transfected them into shMT1G cells. The results showed that MT1G knockdown led to CPT1B activation, and CPT1B knockdown effectively reversed this phenomenon (Fig. 6a, b; Supplementary Fig. 8a). Cell proliferation assay showed that low MT1G expression significantly inhibited cell proliferation. However, re-lowering the CPT1B gene, in addition to MT1G knockdown, by transfecting three different siRNAs targeting CPT1B effectively restored the proliferation-inhibitory effect of MT1G knockdown. Among these, siCPT1B-1 fully complemented the proliferation-inhibitory effect of MT1G knockdown (Fig. 6c). Cell migration assay demonstrated that CPT1B also reversed the weakened migration caused by MT1G knockdown (Fig. 6d). ORO staining experiments confirmed that the increased lipid depletion observed after MT1G knockdown was significantly restored after CPT1B knockdown in both oleic acid-treated and untreated groups (Fig. 6e). These results suggest that MT1G induces intracellular lipid accumulation by suppressing CPT1B in ccRCC cells, thereby promoting ccRCC progression.

### MT1G suppresses CPT1B expression via H3K14 trimethylation modification

Active genes typically possess an open chromatin structure at their promoters, facilitating access to the transcription machinery [18]. The ATAC-seq technique, which detects chromatin accessibility, has been increasingly employed to elucidate gene expression regulation mechanisms [19]. To assess the impact of MT1G on chromatin accessibility, we employed ATAC-seq following MT1G knockdown. IGV browser views revealed a significant increase in chromatin accessibility within the –1000 to +1 bp region of the CPT1B promoter in the MT1G knockdown (shMT1G) group compared to the control group (shNC) (Fig. 6f). To investigate changes in histone modification status resulting from MT1G knockdown or overexpression, we examined six key histone modifications via western blotting. Our results indicated that MT1G overexpression reduced the levels of H3K14ac, H3K14me3, and H3K27ac, while MT1G knockdown increased their levels, with no impact on H3K36me3, H3K36ac, or H3K27me3 (Fig. 6g). To determine whether MT1G influenced the binding of specific proteins (H3K14ac, H3K14me3, and H3K27ac) to the CPT1B promoter region, we employed the chromatin immunoprecipitation qPCR method. We designed four primer pairs targeting different intervals of the CPT1B promoter (–999 to +1 bp regions) (Fig. 6f). Our results indicated that MT1G inhibited the chromatin accessibility of CPT1B by primarily suppressing H3K14me3 binding at sequences 1 and 2, located approximately –968 to -456 bp upstream of the CPT1B promoter (Fig. 6g, h). Due to CPT1B expression maybe controlled by transcription factors, so we utilized a transcription factor prediction website to analyze potential transcription factors binding to the CPT1B promoter region which regulated by MT1G. Our analysis identified three transcription factorsSP1, IRF1, and ICSBP may bind to this region (Supplementary Fig. 8b). To assess the impact of MT1G on the regulation of these transcription factors on the CPT1B promoter, we knocked down these three transcription factors in MT1G-deficient cell lines and evaluated the activity of the CPT1B promoter. Interestingly, we observed that while MT1G knockdown enhanced the promoter activity of CPT1B, knockdown of the three transcription factors did not alter the regulatory effect of MT1G knockdown on the CPT1B promoter (Supplementary Fig. 8c). These results suggest that MT1G regulated CPT1B expressionindependently of transcription factors, potentially through affecting the binding of H3K14me3 to the CPT1B promoter region, subsequently repressing CPT1B expression.

## Discussion

Metabolic reprogramming is pivotal in maintaining cellular homeostasis, with cancer cells often displaying dysregulation in key metabolic pathways, including glucose and fatty acid metabolism [20]. ccRCC, a subtype of RCC, is characterized by the presence of large intracellular lipid droplets [21]. Recent studies have highlighted the significance of aberrant β-oxidation processes in ccRCC, contributing to its tumorigenesis and progression, often indicating poorer prognosis for patients [22, 23]. However, the precise mechanisms underpinning altered lipid metabolism in ccRCC remained elusive until recently. In our study, we have identified MT1G promotion of disease progression in ccRCC patients, despite its low expression in ccRCC. Notably, MT1G enhances lipid accumulation in ccRCC cells through epigenetic repression of CPT1B expression. Knockdown of CPT1B counteracts the inhibitory effects on cell proliferation, migration, and lipid droplet reduction induced by MT1G knockdown. MT1G accomplishes this by inhibiting the binding of H3K14me3 to the CPT1B promoter region. Thus, our study unveils the novel MT1G/H3K14me3/CPT1B signaling axis, which drives lipid accumulation in ccRCC, promoting ccRCC tumor growth and metastasis.

In this study, we have established MT1G as an oncogenic factor in ccRCC. MT1G expression significantly correlates with advanced cancer stage and grade, both at the protein and mRNA levels. Moreover, high MT1G expression predicts poor prognosis in ccRCC patients, as supported by data from GEO and TCGA datasets. In vitro experiments demonstrated that MT1G promoted ccRCC cell proliferation and migration, consistent with Wu Zhang’s findings of MT1G’s role in negatively regulating ferroptosis in ccRCC [23]. In vivo, our subcutaneous neoplasia experiments in NVSG and nude mice consistently showed that MT1G overexpression enhanced kidney tumor growth. Furthermore, we observed that elevated MT1G expression accelerated ccRCC growth in situ. Tail vein lung metastasis assays in both NVSG and nude mice unveiled that high MT1G expression promoted lung and liver metastases, underscoring MT1G’s pro-tumorigenic role in ccRCC. Patients with ccRCC often face a grim prognosis due to chemoresistance and metastatic spread. Sorafenib, an established chemotherapeutic drug for kidney cancer, is hindered by the development of drug resistance, significantly impacting patient outcomes [24]. While MT1G has been reported to promote sorafenib resistance in HCC cells [10], its role in renal cancer remained unexplored. Our study establishes that MT1G inhibits sorafenib sensitivity in ccRCC cells. In summary, MT1G emerges as a promising target for future clinical applications, particularly in patients resistant to sorafenib treatment.

Lipid droplet formation is a defining histological feature in ccRCC but the underlying mechanisms and importance of this biological behavior have remained enigmatic [25]. Suppression of FFA oxidation has been shown to contribute to lipid storage, which is a necessary tumor adaptation. The lipid droplets contain different lipid species (fatty acids (FFAs), triglycerides and cholesterol esters) and non-lipid component (glycogen) [26]. Targeted cholesterol metabolomics revealed the expression of cholesterol was not altered in MT1G high expression cells relative to the control group, suggesting that MT1G promoted lipid droplet accumulation independent of cholesterol. Clinical studies and mechanistic investigations into the roles of different enzymes in FA metabolism pathways have revealed new metabolic vulnerabilities that hold promise for clinical effect [4]. Enzymes involved in the intrinsic FFA metabolism pathway include FFA synthase, acetyl-CoA carboxylase, ATP citrate lyase, stearoyl-CoA desaturase 1, carnitine palmitoyltransferase 1 A and the perilipin family, and each might be potential therapeutic targets in ccRCC owing to the link between lipid deposition and ccRCC risk [3, 27]. In our study, we found for the first time that MT1G can be significantly inhibited the expression of carnitine palmitoyltransferase 1 B (CPT1B), therefore, molecular of MT1G may act as targeted agents to target carnitine palmitoyltransferase. FFA metabolism could potentially be targeted for therapeutic intervention in ccRCC as small-molecule inhibitors targeting the pathway have shown promising results in preclinical models. Storage of excess FFAs is necessary for maintaining endoplasmic reticulum (ER) integrity, and suppressing lipid reactive oxygen species to prevent lipotoxicity [4]. Targeted lipidomics analysis showed that 27.79% of lipids in renal cancer cells were fatty acids, and MT1G overexpression cell lines significantly promoted the total amount of fatty acids. In addition, half of the top 50 metabolites with the highest meaningful fold change and *P* values affected by MT1G high expression were fatty acids. This result suggested that MT1G most likely increased the integrity of the ER and reduced lipotoxicity and thus promoted renal cancer cell growth by promoting increased fatty acids. Elevated lipid storage levels might also benefit ccRCC tumors, as elevated phosphatidylcholine can maintain cell membrane fluidity, which enhances metastatic capability [28], as has been found in other malignancies such as lung carcinoma and glioma. Thus we can explain our results that the augment of phosphatidylcholine after MT1G overexpressing may be why MT1G promoted ccRCC cells invasion and migration in vitro and metastasis in vivo. Metabolomics data show that MT1G high expression lead to glycolysis key metabolites beta-frutose-2,6 bisphosphate upregulation, lactate downregulation. It has been reported that the gluconeogenic enzyme fructose-1, 6-bisphosphatase 1 (FBP1) was uniformly depleted in ccRCC tumors [29]. Whereas the beta-frutose-2,6 bisphosphate was catalyzed by FBP1 to the downstream beta-frutose-1,6 bisphosphate [30], thus explaining why frutose-2,6 bisphosphate was elevated and lactate decreased. The above results suggest that MT1G inhibited the glycolytic phenomenon and promoted the progression of ccRCC. Even though it was not consisted with the “Warburg effect” that cancer cells take large amounts of sugar and produce lactic acid in the presence of hyperglycolysis [31], we concluded that by contrast, the coincident accumulation of glycogen granules in ccRCC was currently thought to be indispensable for tumorigenesis [32], suggesting that both the lipid component and non-lipid component glycogen granules in lipid dropts promotes ccRCC progression. The carnitine palmitoyltransferase system is responsible for delivering the long-chain FFAs from cytoplasm into mitochondria for oxidation, where carnitine palmitoyltransferase I (CPTI) catalyzes the rate-limiting step of FAO [33, 34]. Concurrently, MT1G mediated carnitine palmitoyltransferase 1B (CPT1B) suppression prevents FAO and results in accumulation of cytoplasmic lipids.

In eukaryotes, DNA is compactly organized in nucleosomes (chromatin), where it is intricately associated with histone proteins. These histone modifications play a crucial role in regulating DNA transcription [35]. Recent large-scale sequencing analyses have unveiled significant alterations in histone modifications, underscoring the pivotal role of epigenetic changes in ccRCC [29]. Specific combinations of histone modifications serve as characteristic signatures of gene activity [36–38]. For instance, the presence of histone H3 lysine 36 trimethylation (H3K36me3) and histone H3 lysine 14 trimethylation (H3K14me3) on the gene body is a universal hallmark of active genes [39]. Conversely, developmentally silenced genes often exhibit enrichment in promoter-bound histone H3 lysine 27 trimethylation (H3K27me3) [40] and histone H3 lysine 14 acetylation (H3K14ac) [41, 42]. Certain histone modifications, such as H3K36ac [43] and H3K27ac, are known to promote oncogenic processes in tumors [44, 45]. Our study reveals that MT1G exerts regulatory control over histone modifications. Specifically, MT1G suppresses the expression of H3K14ac, H3K14me3, and H3K27ac, with the high expression of MT1G primarily influencing the interaction of the CPT1B promoter region with H3K14ac and H3K14me3. Chromatin immunoprecipitation followed by quantitative PCR (ChIP-qPCR) results demonstrate that elevated MT1G expression enhanced the binding of H3K14ac to the CPT1B promoter while inhibiting the binding of H3K14me3 to the CPT1B promoter region spanning from -968 to -456 bp. Notably, the DNA in the CPT1B promoter region is considerably less associated with H3K14ac compared to H3K14me3 [46, 47]. This leads us to posit that MT1G primarily hampers CPT1B transcription and translation by impeding the binding of H3K14me3 to the core promoter region of CPT1B.

In summary, our study provides groundbreaking insights into the regulatory role of MT1G in lipid metabolism accumulation. By inhibiting FAO, MT1G promotes the progression of renal cancer. Crucially, MT1G achieves this by suppressing CPT1B gene expression through the regulation of histone modifications. Given the significance of histone modifications in ccRCC development and the success of small molecule histone regulatory agents as therapeutic drugs, MT1G holds promise as a potential targeted drug for renal clear cell cancer.

### Supplementary information

Supplementary materials

WB uncropped figures 24.5.21

## Data Availability

The Original/source data of ATAC-sequencing (accession number: HRA005634) are deposited in the Genome Sequence Archive (GSA, https://ngdc.cncb.ac.cn/gsa/).
