# Supplementary material for: MT1G induces lipid droplet accumulation through modulation of H3K14 trimethylation accelerating clear cell renal cell carcinoma progression
Source: Br J Cancer. 2024 Jun 21;131(4):641–54. doi: 10.1038/s41416-024-02747-y (PMC11333765; doi:10.1038/s41416-024-02747-y)
Supplement: Supplementary file 1 — Supplementary materials [file 41416_2024_2747_MOESM1_ESM.docx]

#### 1.Supplementary materials and methods

#### 1.1 Patient information and bioinformatics analysis

CcRCC cancer and adjacent normal renal tissues were obtained from the Department of Urology at the Affiliated Hospital of Jining Medical College,the collection and study of human clinical samples were performed in accordance with the approved guidelines of the Ethics and Scientific Committees of Affiliated Hospital of Jining Medical College(Ethical number:2021-09-C016). We gathered and analyzed data pertaining to the MT1G transcript and protein levels in both ccRCC and normal tissues, drawing from the TCGA and GEO datasets (specifically GSE6344 and GSE781), as well as the CPTAC dataset (accessed via UALCAN). We also retrieved mRNA levels of various genes from ccRCC patients and relevant clinical data from the TCGA‐KIRC database through the Xena platform (https://xenabrowser.net/heatmap/#). Notably, the TCGA‐KIRC dataset included 533 ccRCC tissues and 72 normal kidney tissues, providing a substantial dataset for our investigation. Furthermore, we expanded our exploration of MT1G across different cancer cell types by obtaining mRNA levels of genes from the HPA RNA‐seq database in the ccRCC single-cell hub, available through The Human Protein Atlas ([www.proteinatlas.org](http://www.proteinatlas.org/)). To evaluate the pathways enriched in our gene set, we employed gene set enrichment analysis (GSEA), assessing these pathways based on their pathway enrichment score (ES). Enriched pathways were determined by using the hypergeometric distribution against the Kyoto encyclopedia of genes and genomes (KEGG) pathway database, as compiled by the MSigDB gene set compendium; significance was achieved for *P* <0.05.

**1.2 ATAC‐seq analyses**

Chromatin accessibility investigation involved the collection of ccRCC and adjacent tissues, along with MT1G knockdown 786-O cells and control cells in 10‐cm dishes. The ATAC‐seq procedure was performed by Genesky Biotechnologies Inc. in Shanghai. Subsequently, libraries were combined in equimolar proportions using barcodes and subjected to sequencing on the BGISEQ‐500 platform, which is operated by BGI in Shanghai, China. For ATAC‐seq analysis, we selected peaks that exhibited significant opening or closing effects, defined as having a |Log2 Fold change| > 1, based on MACS2 bdgdiff analysis. The raw and processed data can be made available upon reasonable request to the corresponding author.

#### 1.3 qPCR assay

Total RNA was extracted from 786-O ccRCC cells using the RNAfast 200 reagent (Shanghai Feijie) following the manufacturer's instructions. The Evo M-MLV RT kit with gDNA Clean for qPCR II (AG11711) was employed for reverse transcription. Quantitative PCR (qPCR) was conducted using the Takara SYBR® Premix Ex Taq™ II (Tli RNaseH Plus) kit (RR820Q) as per the provided guidelines to assess the relative expression of the beta-actin, MT1G genes, as well as fatty acid metabolism related genes CPT1A, CPT1B, and CPT1C in the collected ccRCC tissue samples. The primers were listed as supplementary table 1.

#### 1.4 ROC analysis of MT1G

The diagnostic utility of MT1G in multiple cancers was assessed using receiver operating characteristic (ROC) analysis through the 'pROC' package. Subsequently, the area under the curve (AUC) value, ranging from 0.5 to 1.0, was computed. A higher AUC value signifies a stronger diagnostic capability. In general, AUC values falling within the ranges of 0.5–0.7, 0.7–0.9, and 0.9–1.0 indicate low, moderate, and high predictive efficacy, respectively.

#### 1.5 Fluorescent immunohistochemical staining of MT1G

CcRCC cancer and paracancerous tissues were first fixed in 4% paraformaldehyde, followed by embedding in paraffin. The formalin-fixed wax blocks were subsequently subjected to dewaxing and hydration. Antigen retrieval was achieved through microwave treatment, and blocking was performed using sheep serum for 1 h. The primary antibodies (MT1G at a 1:50 dilution, [Cusabio](http://www.baidu.com/link?url=qjelGCDKd6U-zMlRvrsZGp2b3lg9WEl6EAtxYx9vH9cfAUsxajzI36riDVg3Ah73bbQRKB3YrJKBeqmGJdWla_), and CPT1B at a 1:50 dilution, abclonal) were incubated at room temperature for 4 h, followed by a 10-min incubation with secondary antibodies. A 1× dye working solution (100 μL, diluted at 1:100) was added to the glass slide and incubate at room temperature for 10 min. Antigen retrieval via microwave treatment was followed by natural cooling to room temperature. Afterward, 1 μl of DAPI working solution was added and incubated at room temperature for 5 min to stain nuclear. After three washes with TBST, an anti-fluorescence quenching sealing agent was applied, and the edges of the cover slide were sealed with clear nail polish, followed by analysis under a fluorescence microscope (abs50012).

#### 1.6 Cell culture and treatment

CcRCC cells 786-O and A498 cells were purchased from the National Collection of Authenticated Cell Cultures. These cells were cultured with RPMI 1640 medium and F12K DMEM medium supplemented with 10% fetal bovine serum (GIBCO No.: 10099141) and 1% penicillin/streptomycin (Sigma), and were authenticated by STR profiling and tested for mycoplasma contamination.

#### 1.7 Cell proliferation assay

#### Cell proliferation was assessed using the cck8 assay. Briefly, 786-O or A498 cells were seeded into 96-well plates at a density of 5×103 cells per well. Following the designated treatment, 20 μl of cck8 solution was added to each well and incubated for 1.5 h. Subsequently, the plates underwent brief low-speed shaking for 1 min and the absorbance was measured at OD 490 nm.

#### 1.8 Cell cycle

Cell cycle analysis was performed utilizing a Cell Cycle Staining Kit (Hangzhou KeyGEN BioTECH) in strict accordance with the manufacturer's guidelines. In summary, 786-O and A498 cells were harvested, washed, fixed in pre-cooled methanol for 24 h, and treated with 0.4 ml of DNA staining solution, along with 10 µl of Propidium Iodide and 4 μl of RNAase, all at 4°C in the dark for 30 min. Finally, single-cell suspensions were analyzed using a BD FACS Calibur flow cytometer (BD Biosciences). Data were analyzed using FlowJo software v10.0.8 (BD Biosciences).

#### 1.9 Cell apoptosis assay

Cell membrane integrity was assessed employing an Annexin V-FITC/PI Apoptosis Kit (Hangzhou KeyGEN BioTECH according to the manufacturer's instructions. Briefly, 786-O and A498 cells were treated with 500 μl of Binding Buffer and gently agitated to attain single-cell suspension. Subsequently, 5 μl of Annexin V-FITC and 5 μl of Propidium Iodide were added, mixed, and incubated for 5~10 min in the dark. Finally, cell analysis was conducted utilizing a BD FACS Calibur flow cytometer, and the acquired data underwent analysis using FlowJo software v10.0.8.1 (BD Biosciences).

#### 1.10 Transwell cell migration and invasion assay

Following 24 h of the prescribed treatment, the cells were harvested, resuspended in serum-free medium, and 100 μl cell suspension containing 5 × 10^4 cells was added to the upper chambers of a transwell culture plate. In the lower chambers, 500 μl of medium containing 10% FBS was added. The plate was incubated at 37 °C for 24 h, the cells on the upper surface of the polycarbonate films were gently removed with wet cotton swabs, followed by fixation in pre-cooled methanol for 30 min. Cells were then stained with a 1% crystal violet solution for 15 min, washed three times with PBS, and examined under a microscope.

#### 1.11 Western blotting

#### Cells were homogenized in ice-cold RIPA buffer supplemented with a proteinase inhibitor cocktail (Sigma-Aldrich). Protein concentrations were determined using a BCA protein assay kit (Thermo Fisher). Equal amounts of protein were then separated on SDS polyacrylamide gels, followed by immunoblotting with the following primary antibodies: anti-cyclin D1 (1:1,000, CST), anti-cdk2 (1:1,000, CST), anti-cdk4 (1:1,000, CST), anti-cdk6 (1:1,000, CST), anti-p21 (1:1,000, CST), anti-MT1G (1:1,000, Omnimab), anti-CPT1A (1:1,000, Abclonal), anti-CPT1B (1:1,000, Abclonal), anti-CPT1C (1:1,000, Abclonal), anti-β-actin (1:2000, CST), anti-histone H3-K27ac (1:1000, Abclonal), anti-histone H3-K36ac (1:1000, CST), anti-histone H3-K14ac (1:1000, CST), anti-trimethyl-histone H3-K14(1:1000, Abclonal), anti-trimethyl-Histone H3-K36 ((1:1000, Abclonal), anti-trimethyl-histone H3-K27(1:1000, Abclonal), and anti-H3 (1:1000, Proteintech). Membranes were then incubated with a peroxidase-conjugated secondary antibody, and specific bands were detected using an imaging system.

#### 1.12 Xenograft experiments in mice

All animal experiments received approval from the Animal Ethics Committee of Jining Medical College (Approval No. 2021B097). Four-week-old male BALB/c nude mice and NVSG mice, the internationally recognized tool mice with the highest degree of immunodeficiency, were purchased from Jinan Pengyue Experimental Animal Breeding Co., LTD. The mice were subcutaneously administered with either 1×107 MT1G over-expressing cells, MT1G knockdown cells OENC or shNC control 786-O cells, with each group consisting of five or three mice. The mice were housed with a 12-h light/dark cycle. All procedures strictly adhered to established guidelines. Ultimately, the mice were euthanized with CO2, and the resultant tumor samples were subjected to H&E, IHC.

#### 1.13 Immunohistochemical (IHC) and HE staining

IHC staining was performed according to the kit instructions described as IHC kit ([BOSTER Biological Technology co.ltd](https://www.baidu.com/link?url=f9FiAGflB12QX_UIxG-AyjHSLwC4oXG-9zUaf_u0mhmYoAG_-DQ8lX1-rmmjLD_x&wd=&eqid=f4fb4a0000025cce00000002652c9c46) ) . Primary antibodies employed for IHC staining included MT1G (1:100, CUSABIO), Ki67(1:100), E-cadherin(1:100), N-cadherin(1:100) and Vimentin(1:100) (CST). HE staining was performed HE staining kit followed as Beijing Leagene Biotechnology co.ltd.

**1.14 Orthotopic xenograft tumor model and IVIS Imaging**

A total of 1×106 cells suspended in 0.1 ml PBS were injected into the subcutaneous of NVSG mice, with each group consisting of 1 mice at 4 weeks of age. Two weeks later, subcutaneous tumors were dissected and the subcutaneous tumor was washed by PBS for three times using sterile scissors to cut the tumor into 1 mm3 pieces. After anesthesia with chloral hydrate, hair was shaved off near the right abdomen and disinfected with iodine. A 1cm incision was made on the skin and peritoneum, and the kidney was removed. Small pointed forceps were used to puncture the kidney, and the small lump was inserted into the kidney and sent back to the kidney. The peritoneum skin was sutured, and the surgical incision was disinfected with iodine. The signal intensity emanating from luciferase-labeled cells within orthotopic and metastatic lesions was monitored once per week in vivo, employing a bioluminescence imaging system (IVIS 200, PerkinElmer). Mortality rates were documented. Subsequently, tissue samples were harvested, measured, and evaluation of kidney tumor size, lung and liver metastases was carried out based on GFP signals utilizing the Axio ZoomV16 luciferase imaging system (ZEISS).

#### 1.15 Tail vain injection and IVIS Imaging

A total of 2×106 786-O cells, either overexpressing MT1G or serving as control and carrying a luciferase/GFP vector, were suspended in 100 µl of PBS and intravenously injected into the NVSG mice. Intraperitoneal administration of D-luciferin (75 mg/kg) (122799, PerkinElmer) was conducted, and imaging of the mice in the prone position was acquired 15 min post-injection using the IVIS Imaging System (IVIS 200, PerkinElmer) at 3 and 6 weeks to track the in vivo luciferase signal for tumor development. Following euthanasia with CO2, lung and liver samples were harvested at 4 weeks post-tail vein injection, and evaluation of lung and liver metastases was carried out based on GFP signals utilizing the Axio Zoom V16 luciferase imaging system (ZEISS). Mortality rates were recorded, and all mice were sacrificed at the 8-week post-injection. Tissue samples were collected and measured.

#### 1.16 Lipid droplet staining

For fat droplet staining, a modified Oil Red O dye (Beijing Leagene) was employed. Initially, MT1G over-expression or knock down cell lines and control cells were seeded into 6-well plates and cultured in the presence or absence of oil droplets for a duration of 24-48 h. The culture medium was then discarded, and the cells were washed with PBS, fixed with 4% paraformaldehyde for 20 min, and then stainedfor 30 min. After five washes with PBST, the cells were examined microscopically.

#### 1.17 Untargeted metabolomic analysis

Extraction and sample preparation adhered to an established protocol provided by Shanghai Lu-Ming Biotech Co. Ltd. (Shanghai, China, 2021). Non-targeted metabolite analysis was performed by OE Biotech (Shanghai, China). Metabolic profiling was carried out employing a Dionex Ultimate 3000 RS UHPLC system, which was equipped with a Q-Exactive quadrupole-Orbitrap mass spectrometer and a heated electrospray ionization (ESI) source (Thermo Fisher Scientific, Waltham, MA, USA). The analysis encompassed both ESI-positive and ESI-negative ion modes utilizing an ACQUITY UPLC BEH C18 column (1.7 μm, 2.1 × 100 mm^2^).Quality controls (QCs) were conducted at regular intervals, specifically after every 10 samples, during the analytical run to ensure the generation of a reproducible dataset. The acquired LC-MS raw datasets were then subjected to analysis employing the Progenesis QI software (Waters Corporation, Milford, USA).

#### 1.18 Targeted metabolomics analysis

A targeted lipidomics technology platform (UPLC-TQMS) was employed for the quantitative analysis of lipids in biological samples, sourced from Metabo-Profile in Shanghai, P.R. China. Raw data files originating from UPLC-MS/MS analyses were processed using the TMBO software (v.1.0, Metabo-Profile) to conduct peak integration calibration and metabolite quantitation for each compound. Principal component analysis and statistical analyses were performed using the iMAP platform (v.1.0, Metabo-Profile Biotechnology).

#### 1.19 siRNA and Plasmid transfection

#### When the cell density in the 6-well plates reached approximately 80%, and the cells exhibited robust growth without mycoplasma contamination, a fresh serum-containing medium was introduced before initiating transfection. This involved the combination of 200 μL Optimem medium and 20 μL RFect in a 1.5 ml sterile centrifuge tube, followed by gentle mixing, and a 5-min incubation at room temperature to create Solution A. In a separate 1.5 ml sterile centrifuge tube, 200 μL Optimem medium was combined with 8 ml of Solution A and either 5 μL of siRNA (20 μM) or 4 μg of plasmid, leading to the formation of Solution B. These two solutions were gently mixed and allowed to stand at room temperature for 15-20 min. Subsequently, the configured transfection complex was added to the petri dish, gently agitated, and immediately placed in a 37℃ incubator. After 8 h, the medium was replaced.

#### 1.20 Chromatin immunoprecipitation qPCR assay

The Magna ChIP™ A/G One-Day Chromatin Immunoprecipitation Kits (sigma,17-10085) were utilized to assess the binding of specified histones to the CPT1B promoter region. The procedure were performed according to the instructions, and the primary antibodies using ratio were H3K27ac (1:50, Abcam, ab4729), H3-K14ac(1:50, CST,7627T), H3-K14me3 (1:50, Abclonal,A5279), H3 (1:50, Proteintech, 17168-1-AP) and IgG (1:50, Proteintech, 30000-0-AP). Sonication was performed with : 30sec ON–30sec OFF, 20 cycles, at 200W power with break a 2min pause on ice every cycles.To check sonication-efficiency, 10μL of sample was reverse-crosslinked by adding 40μL ChIP elution buffer. DNA was incubated at 65°C for 1h and 1μL RNase A (10mg/mL) was added to sonicated DNA and incubated at 37°C for 1h. Finally, 5μL Proteinase K (20mg/mL) were added and incubate at 55°C for 2h. An 1.5% agarose gel was run to check DNA size distribution. After DNA purification, q-PCR was performed using SYBR Green Master mix. The sequence of the CPT1B promoter primer seq1-4 were showed as Supplematray table 1.

#### 1.21 Statistical Analysis

Statistical analyses were conducted using SPSS software version 26.0 (SPSS Inc., Chicago, IL, USA) and Python 3.8.0 (https://www.python.org). The Chi-square test was employed to compare patient characteristics between the high expression and low expression cohorts of MT1G. The AUC was calculated to assess the predictive model's performance. Data are presented as the mean ± SD. **** represented *P* <0.0001, *** represented *P* < 0.001, ** represented *P* < 0.01, and * represented *P* < 0.05.

**2.Supplementary figure legends**

**
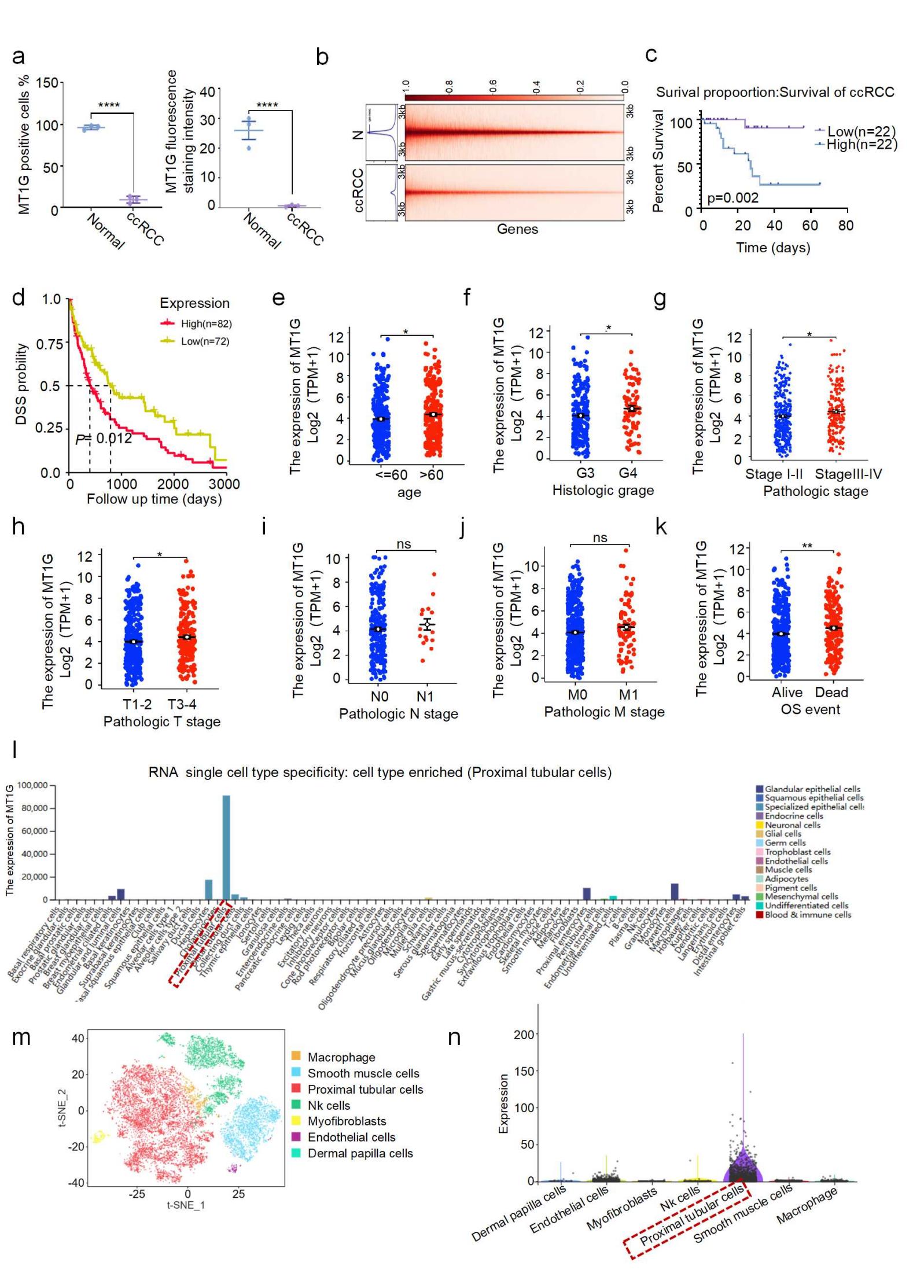
**

Supplementary Fig.1. Analysis of prognostic and clinical parameters affected by MT1G in ccRCC.a. Statistical analysis of immunofluorescence staining-positive cells and staining intensity for MT1G protein in ccRCC tissue and normal tissue (*****P*<0.001). a Heatmap analysis of ATAC-seq data depicting gene accessibility in ccRCC tissue and normal tissue, with stronger red color indicating higher gene openness. c. Survival curve analysis of 44 ccRCC cancer patients categorized into low and high MT1G expression groups based on median MT1G expression (*P* =0.002). d. Relapse-free survival analysis of KIRC patients with high and low MT1G mRNA levels from the KM-plotter analysis, *P* = 0.012). e-k. T-test analysis examining the correlation between MT1G expression and clinical parameters in the TCGA data (**P* <0.05, ** *P* <0.01). l. Analysis of MT1G expression in different types of tumor cells of ccRCC based on single-cell sequencing data from the Human Protein Atlas online dataset. m. RCC 1 (pT1), RCC2 (pT2) and RCC 5 (pT3) patient data were downloaded from the single-cell sequencing data GSE224630 and cell subsets were classified according to the cell marker gene. n. RCC 1 (pT1), RCC2 (pT2) and RCC 5 (pT3) patient data were downloaded from the single-cell sequencing data GSE224630 and cell subsets were classified according to the cell marker gene. MT1G gene expression in Macrophage,Smooth muscle cells,Proximal tubular cells,Nk cells, Myofibroblasts, Endothelial cells and Dermal papilla cells in above three renal clear cell carcinoma tissues were analyzed for statistical analysis.


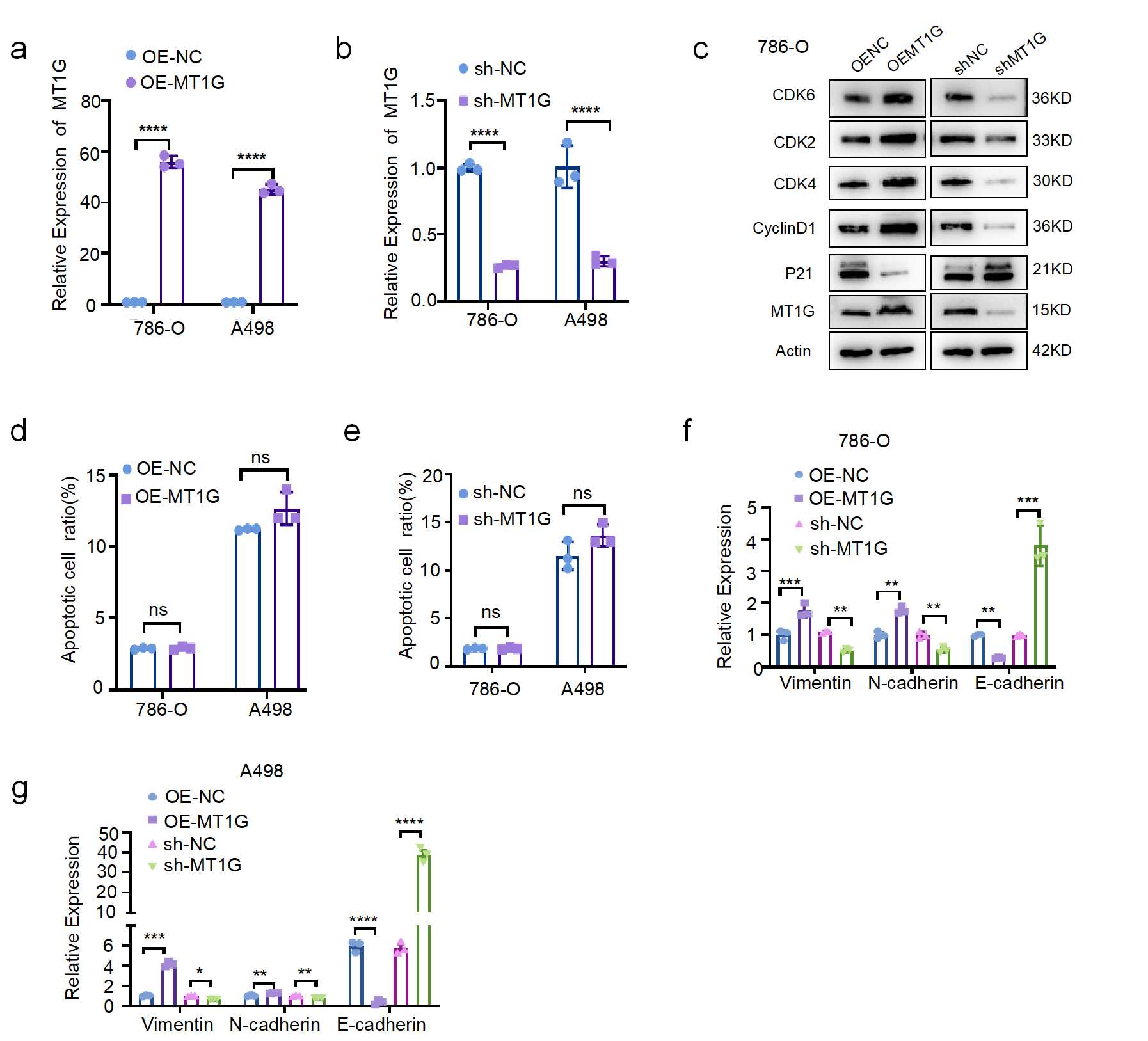


Supplementary Fig.2. Analysis of cell cycle, apoptosis, EMT marker expression affected by MT1G in ccRCC cells. a Detection of effective MT1G overexpression by qPCR in human KIRC cell lines 786-O and A498 after infection with MT1G-overexpression lentivirus. a Verification of effective MT1G knockdown by qPCR in human KIRC cell lines 786-O and A498 after infection with MT1G-knockdown lentivirus. c. Western Blot analysis of cell-cycle-related protein indicators (CDK6, CDK2, CDK4, Cyclin D1, P21) at the protein level in stably overexpressing or knockdown MT1G and corresponding control transfected 786-O cells, with actin as an internal reference. d-e. Cell apoptosis analysis using a cell apoptosis staining kit. Flow cytometry analysis of PI and AV markers in MT1G-overexpressing or MT1G-knockdown 786-O and A498 cells. f and g. qPCR analysis of EMT related genes in human KIRC cell lines 786-O and A498 after infected MT1G-overexpressing or MT1G-knockdown lentivirus.


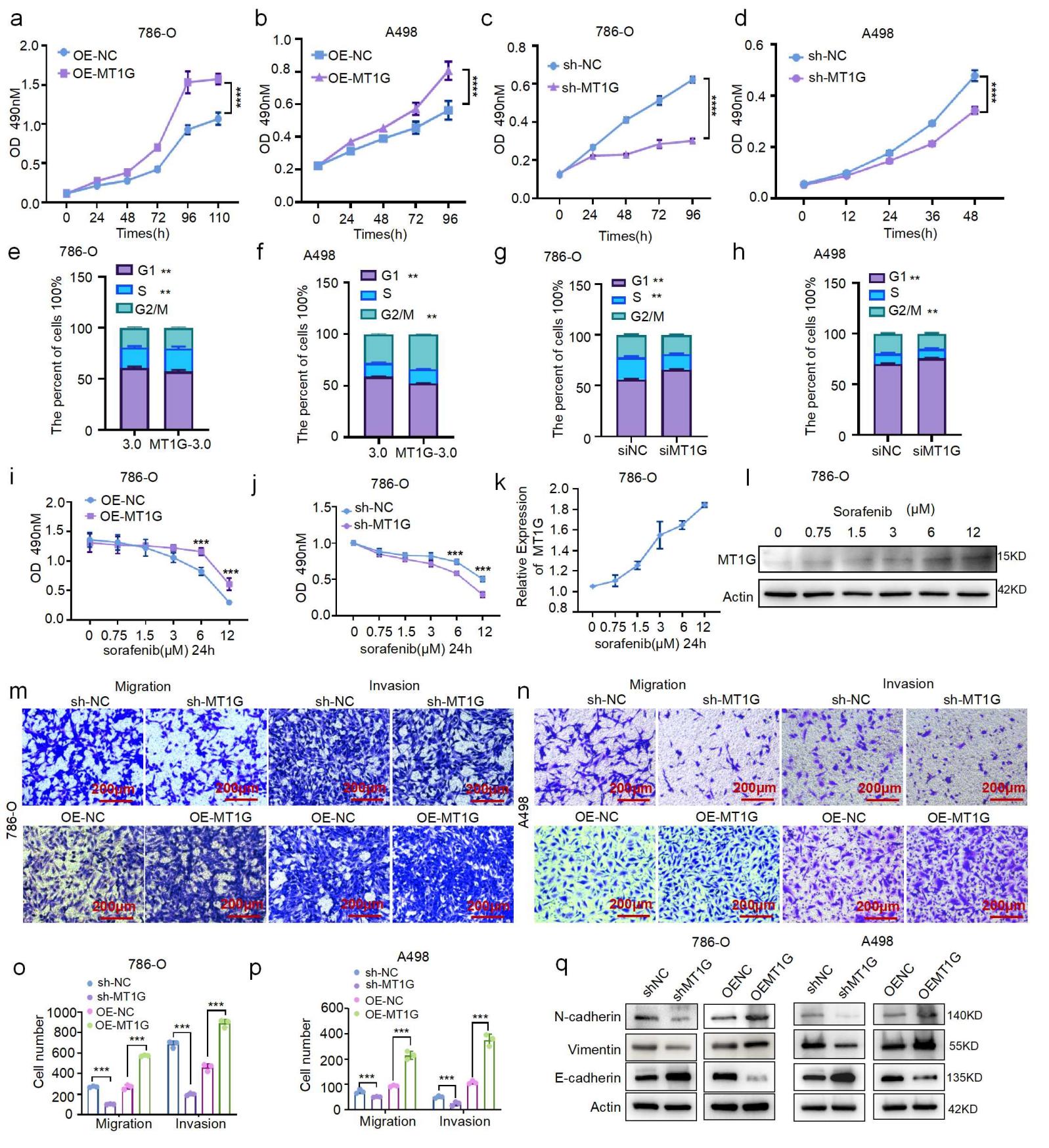


Supplementary Fig.3. Analysis of MT1G-induced effects on ccRCC proliferation, cell migration, and sorafinib sensitivity *in vitro*. A and a Cell proliferation assay (*n*=5) comparing the proliferation ability of MT1G-overexpressing (OE-MT1G) and control (OE-NC) 786-O and A498 cells. c and d. Cell proliferation assay (*n*=5) comparing the proliferation ability of MT1G-knockdown (sh-MT1G) and control (sh-NC) 786-O and A498 cells. e and h. Cell cycle analysis using a staining kit and flow cytometry analysis of PI markers in MT1G-overexpressing or MT1G-knockdown 786-O and A498 cells. i and j. Cell proliferation assay (*n*=5) after treatment with different concentrations of sorafenib in MT1G-overexpressing or MT1G-knockdown cells, examining the relationship between MT1G and sorafenib sensitivity. k. qPCR analysis of MT1G expression at the mRNA level in 786-O cells treated with different concentrations of sorafenib, with actin as a control gene. l. Western Blot analysis of cell protein extracted from 786-O cells treated with different concentrations of sorafenib, with actin as a control gene. m and n. Migration assay in 786-O and A498 cells with varying MT1G expression levels. o-p. Statistical analysis of the number of migratory cells. q. Western Blot analysis of EMT-related genes in human KIRC cell lines 786-O and A498 after infection with MT1G-overexpressing or MT1G-knockdown lentivirus. Data are presented as the mean ± SD. ****P* < 0.001, ***P* < 0.01, and **P* < 0.05 as determined by the T-test.


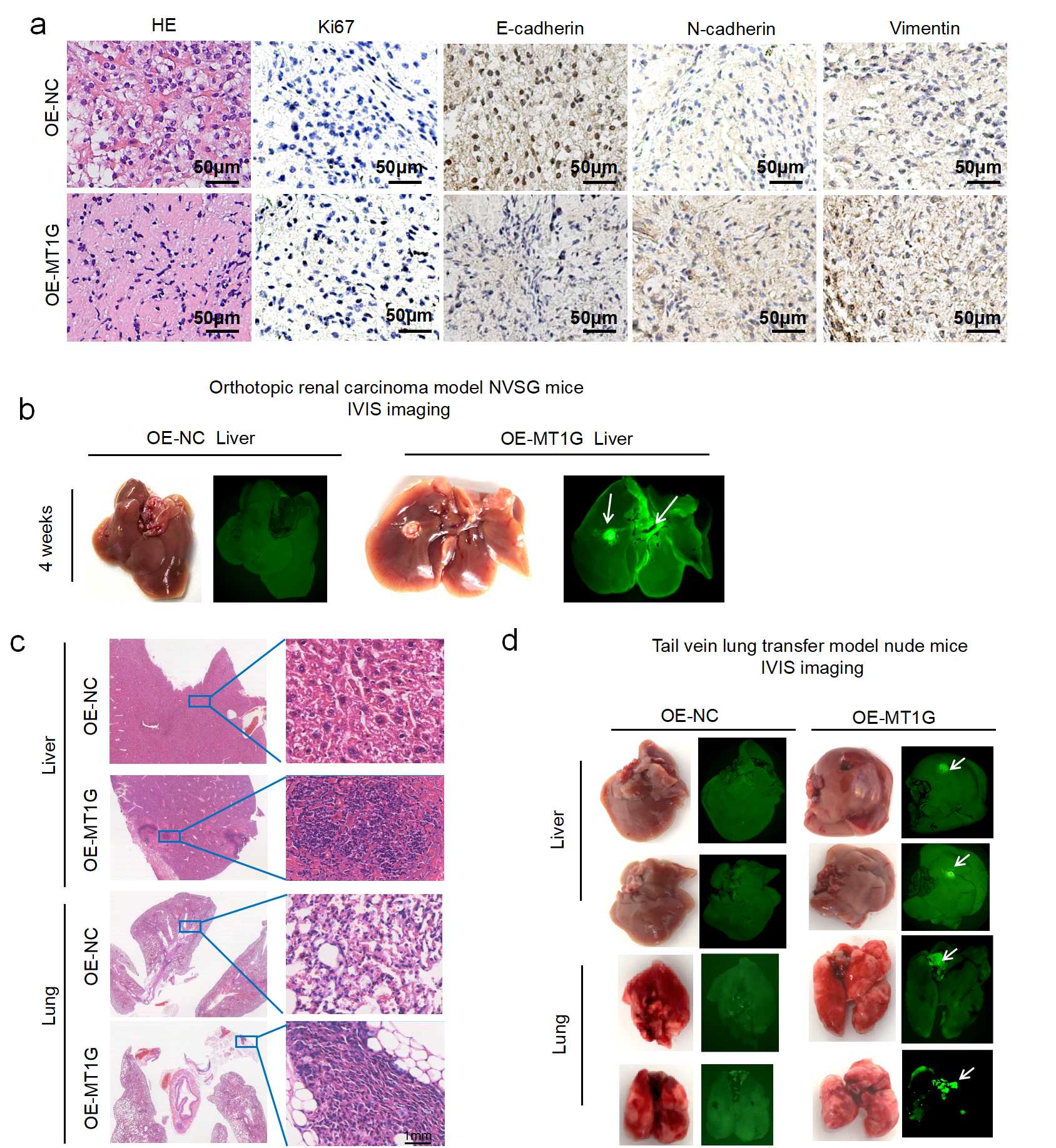


Supplementary Fig.4. Analysis of MT1G-induced effects on ccRCC through HE staining, Ki67, and EMT Markers by IHC. a IHC analysis of HE staining, Ki67 protein expression, and EMT markers in MT1G-overexpressing RCC cells. Statistical analysis of the staining intensity was performed (*****P* <0.001). b. GFP signals analysis of OEMT1G and OENC orthotopic clear cell renal carcinoma mice and tail vein lung transfer model nude mice by using the Axio Zoom V16 luciferase imaging system (ZEISS). Three of OEMT1G mice that were preferentially dead, three of OENC mice were randomly sacrificed as control mice, and kidneys from above mice were harvested and Axio Zoom V16 luciferase imaging based on GFP were performed. Representative fluorescent pictures of the liver and lung in OEMT1G group and OENC group mice with an orthotopic renal carcinomamice at week 4 were shown. c. HE staining of the liver and lung in the orthotopic renal carcinoma model mice. d. GFP signals analysis of OEMT1G and OENC tail vein lung transfer model nude mice by using the Axio Zoom V16 luciferase imaging system (ZEISS). Three of OEMT1G mice that were preferentially dead, three of OENC mice were randomly sacrificed as control mice, and liver and lung from above mice were harvested and Axio Zoom V16 luciferase imaging based on GFP were performed. Representative fluorescent pictures of the liver and lung in OEMT1G group and OENC group mice with an orthotopic renal carcinomamice at week 4 were shown.


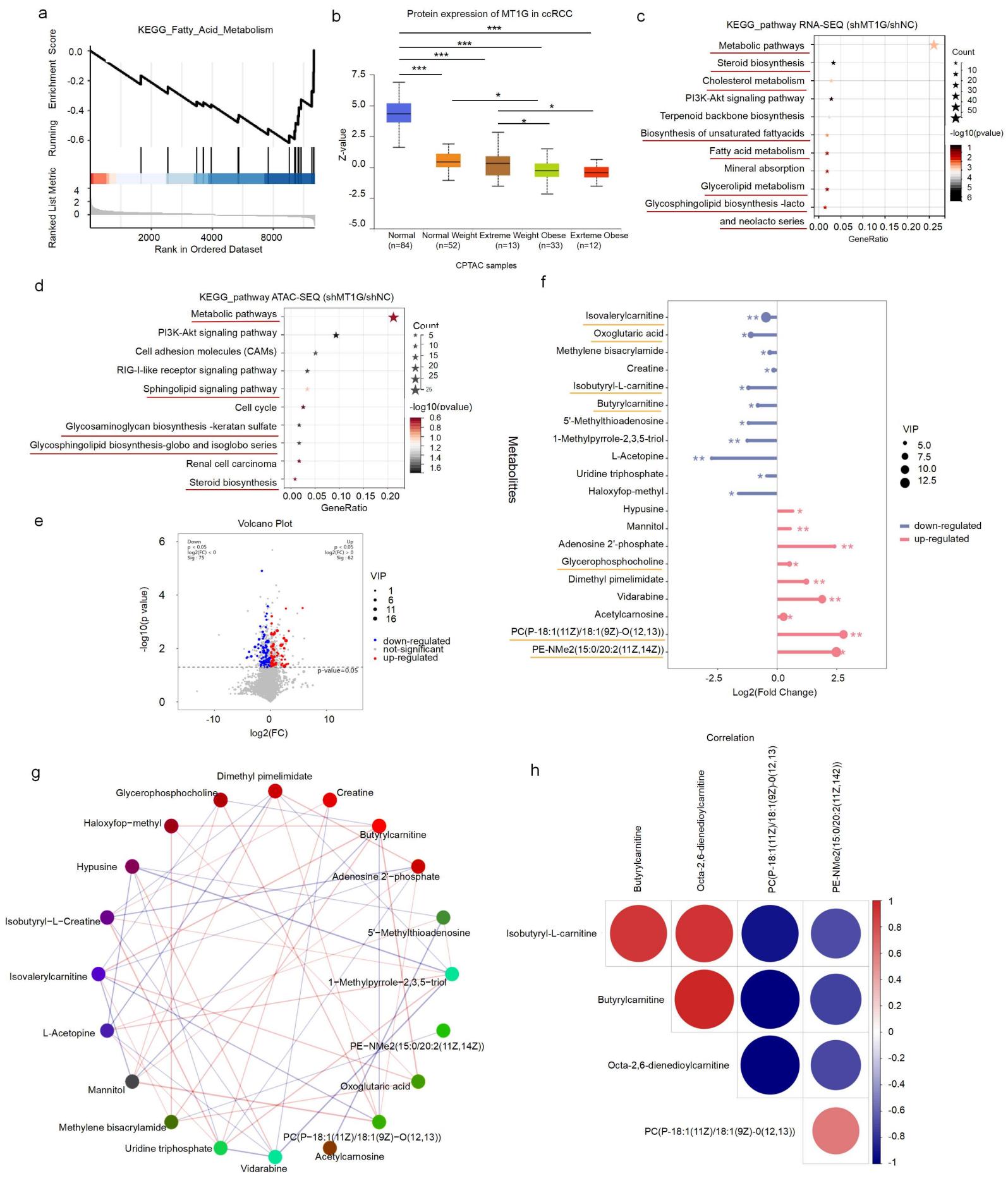


Supplementary Fig.5. Correlation between MT1G and lipid metabolism in ccRCC. a GSEA of MT1G expression in TCGA datasets, showing high and low expression groups. b. Analysis of the relationship between MT1G and obesity in ccRCC patients using the UCLCAN database. c-d. KEGG analysis of differentially expressed genes from transcriptome sequencing and ATAC-seq data induced by MT1G knockdown. e. Volcano plot analysis of differential metabolites, with red points indicating significantly up-regulated metabolites and blue points indicating significantly downregulated metabolites (*P* <0.05, VIP> 1, and FC <1). f. Analysis of fold changes and *P* -values for the top 20 VIP-scored differential metabolites. Red columns represent upregulated metabolites, and blue represents downregulated metabolites (*****P* <0.0001, ****P* < 0.001, ***P* < 0.01, and **P* < 0.05). g. Correlation network diagram analysis, depicting associations between differential metabolites and response intensity data (*P* <0.05 and correlation > 0.95). Shapes represent differential metabolites, and connections indicate associations (red for positive, blue for negative). h. Correlation analysis between the top 5 significantly different metabolites using Pearson correlation coefficient (red for positive, blue for negative).


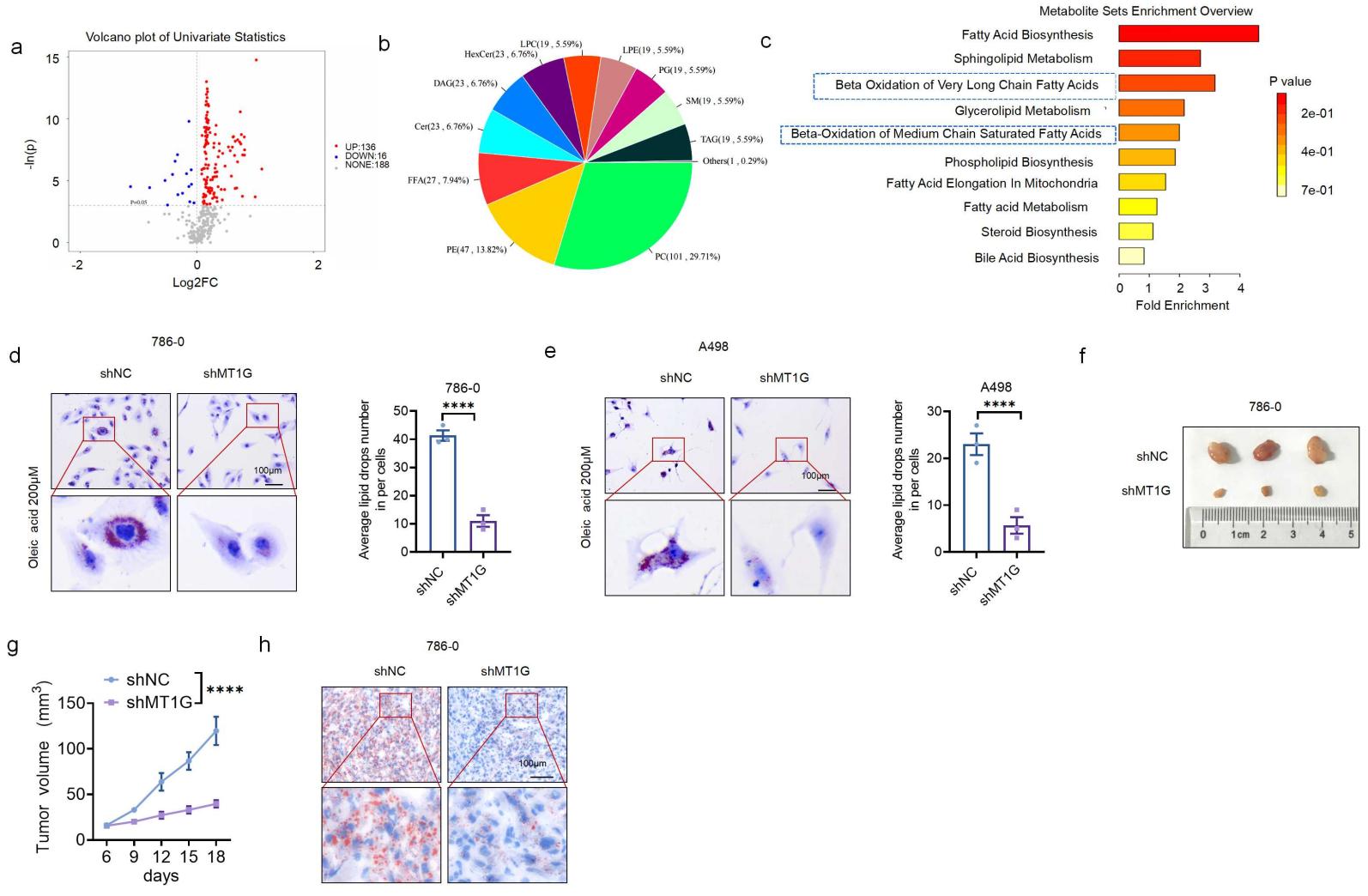


Supplementary Fig.6. Targeted Lipidomics in Response to MT1G. a. Volcano Plot illustrates fold change (FC) and p-value for each metabolite. The selection criteria for differential metabolites were set at *P* < 0.05 and |log2FC| >= 0. In the plot, red-highlighted points indicate metabolites increased in OEMT1G, while blue-highlighted points indicate decreased metabolites in OEMT1G. b. Pie chart analysis displays the proportions of differential metabolites. c. Pathway enrichment analysis using Pathway-associated metabolite sets (SMPDB). d and e. Detection and quantification of lipid droplets using ORO staining. 786-O and A498 cells with stable MT1G knock down and control cells were subjected to 200μM oleic acid, and lipid droplets were quantified using Image J software.*****P*<0.0001, ****P* < 0.001, ***P* < 0.01, and **P* < 0.05, determined by the T-test. f. Subcutaneous injection of 786-O cells stably knock down MT1G or control virus into severe immunodeficient mice (NVSG) (n = 3). Subsequently, xenograft tumors were dissected and representative images were obtained at day18 post-injection. g. Tumor growth curve during the inoculation period. Data are presented as the mean ± SD, n = 5. ****p<0.0001, ****P* < 0.001, ***P* < 0.01, and **P* < 0.05 as determined by the T-test. h. The dissected subcutaneous tumor tissue was divided into frozen sections followed by oil red O staining and photographed.


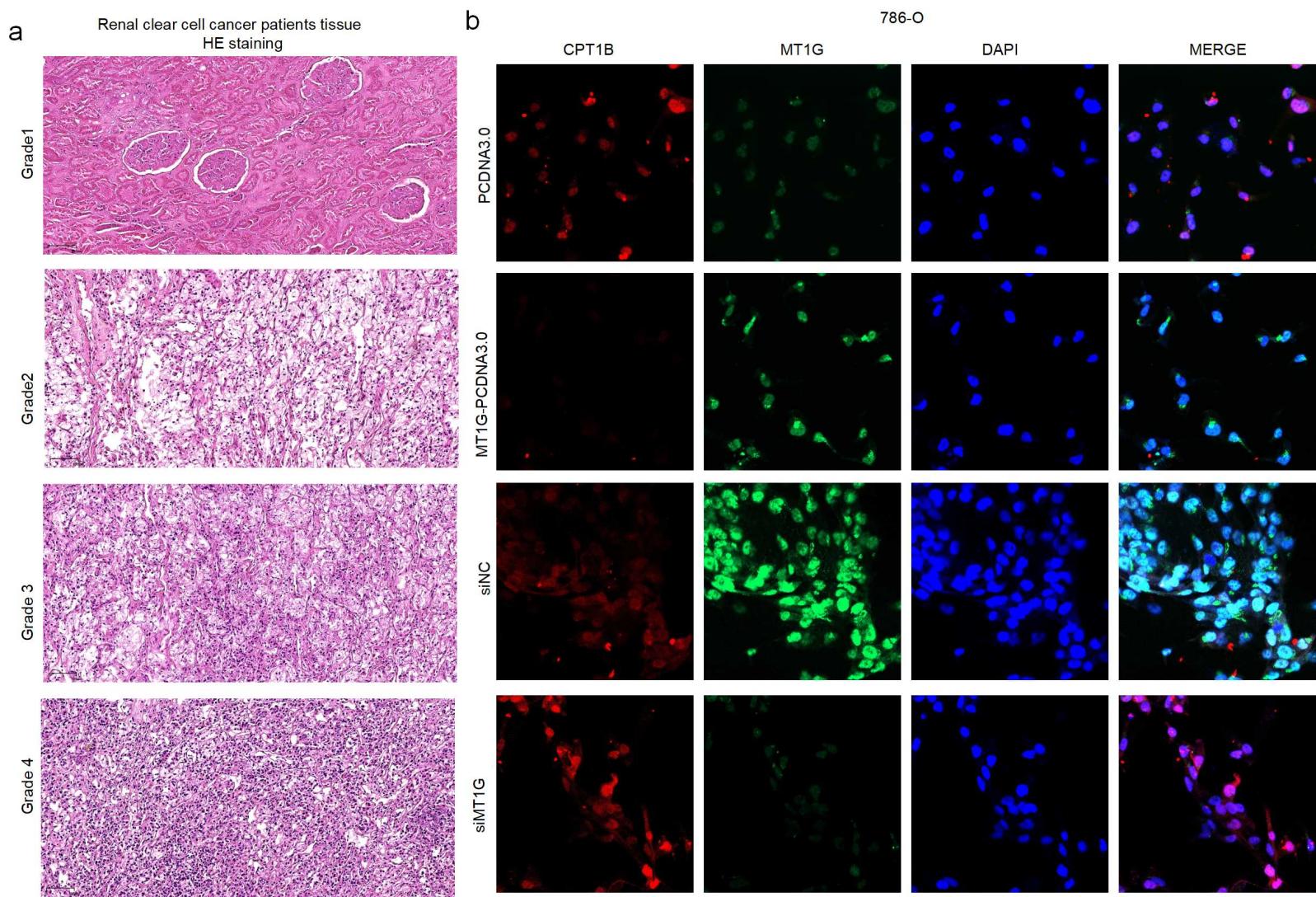


Supplementary Fig.7. Association analysis between MT1G and CPT1B. a. HE staining analysis of different grades of ccRCC patient tissues used for immunofluorescence experiments. b. Correlation analysis of MT1G and CPT1B expression through immunofluorescence experiments. Transfection of siRNA-MT1G resulted in MT1G knockdown in 786-O cells, while transfection of PCDNA-3.0-MT1G led to MT1G overexpression. MT1G and CPT1B antibodies (1:50) were used for immunofluorescence experiments.


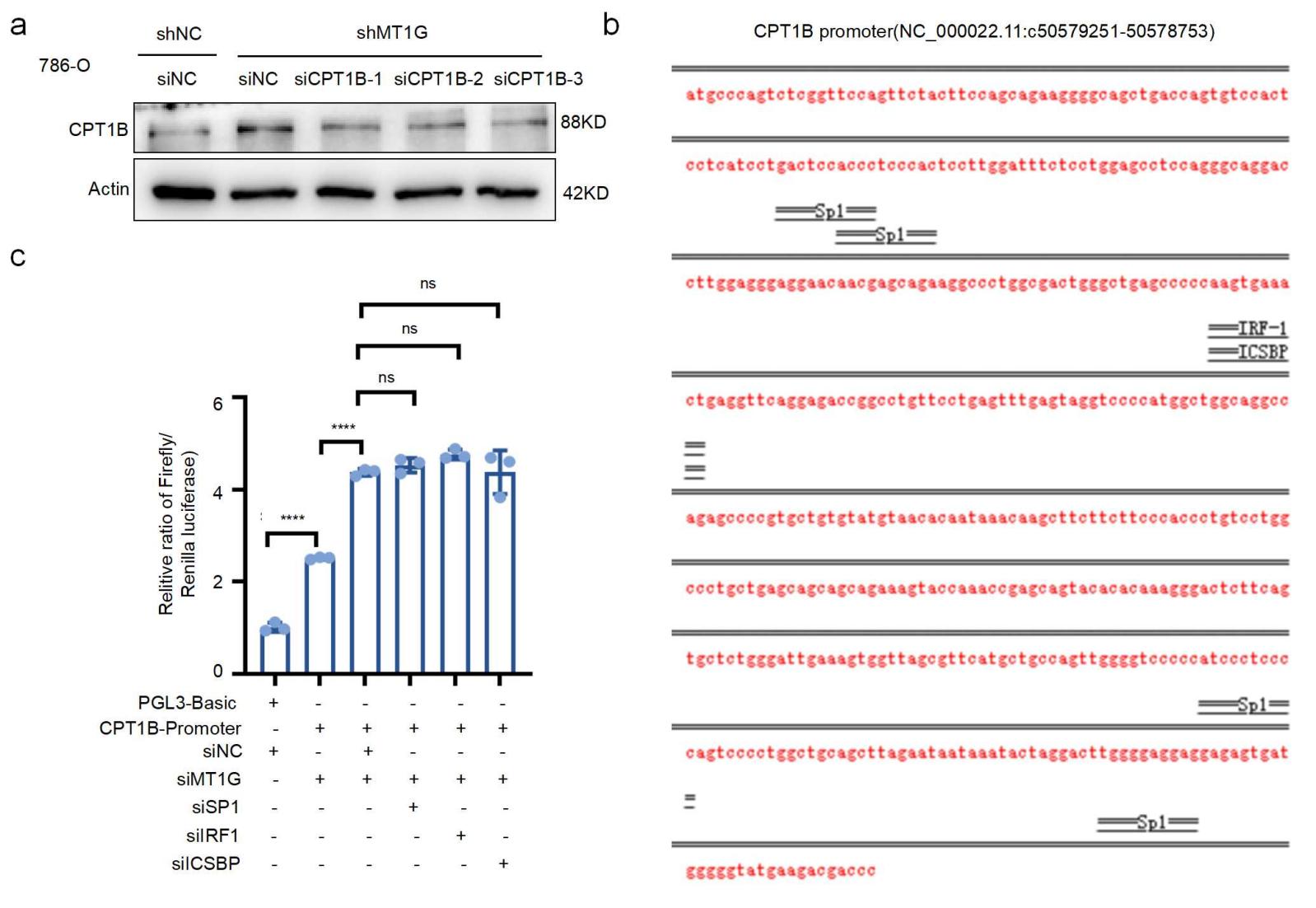


Supplementary Fig.8. a. Analysis of CPT1B knockdown efficiency after MT1G knockdown by western blot in 786-O shMT1G cells. The 786-O cell line with a stable knockdown of MT1G and control group cells were transfected with CPT1B siRNA-1, -2, and-3 or siNC for 48h, cells were harvested and the protein levels of CPT1B and Actin was determined by western blot. Actin was used for internal reference. b. The open region sequence of the CPT1B promoter caused by MT1G knockdown (NC_000022.11: c50579251-50578753) was constructed for luciferase reporter plasmid, and analyzed the potential transcription factors bound to it using the AliBaba 2.1 online database. The transcription factor site binding rate was selected above 80%. c. The siRNA sequence of IRF-1 was designed and the targeting sequence is "GCGTGTCTTCACAGATCTGAA"; the siRNA sequence of SPI is "CCTTCACAACTCAAGCTATTT"; and the siRNA sequence of ICSBP and the targeting sequence is "GCCCGCATCATGATTAAAGAA". The above three siRNA were cotransfected with MT1G-siRNA or siNC in 786-0 cells. After 24h, the PGL 3-Basic plasmid or CPT 1 B promoter plasmid combined with the Rlina plasmid were cotransfected into 786-O again, and 48h later, dual-luciferase reporter gene assay kit was used to examine firefly luciferase activity and renilla luciferase activity.Finally, the ratio of firefly luciferase activity/ renilla luciferase activity were statistically analyzed.

**3.Supplementary Table1** **The primer list of the indicated genes**

| Primer names ^a^ | Forward | Reverse |
| --- | --- | --- |
| MT1G | 5'-AGAGTGCAAATGCACCTCCTGC-3' | 5'-TTGTACTTGGGAGCAGGGCTGT-3' |
| β-actin | 5'-GAAGAGCTACGAGCTGCCTGA-3' | 5'-CAGACAGCACTGTGTTGGCG-3' |
| CPT1A | 5'-ATCAATCGGACTCTGGAAACGG-3' | 5'-TCAGGGAGTAGCGCATGGT-3' |
| CPT1B | 5'-CACGGACAGGAGTGAACCC-3' | 5'-CAGGCGTTTCTTCCAGGAGT-3' |
| CPT1C | 5'-GGCTAGGGACACGAGAGAGA-3' | 5'-GTCCAATCCCAGTGCAAGGA-3' |
| seq1 | 5'-AGGGAAAAGGTCACCGATGT-3' | 5'-CCAAACCGAGCAGTACACAC-3' |
| seq2 | 5'-GGACAGGGTGGGAAGAAGAA-3' | 5'- CTCATCCTGACTCCACCCTC-3' |
| seq3 | 5'-TCGTTGTTCCTCCCTCCAAG-3' | 5'-TCCCTAGGACTATGCCCAGT-3' |
| seq4 | 5'-GACCACAGACCCCAGAAGAA-3' | 5'- GTGGAGTTAGGACAGTGGGG-3' |
| SP1 | 5'-TGGCAGCAGTACCAATGGC-3' | 5'-CCAGGTAGTCCTGTCAGAACTT-3' |
| IRF1 | 5'-ATGCCCATCACTCGGATGC-3' | 5'- CCCTGCTTTGTATCGGCCTG-3' |
| ICSBP | 5'-ATGTGTGACCGGAATGGTGG-3' | 5'-AGTCCTGGATACATGCTACTGTC-3' |

^a^ The above gene primers were designed by using the primer 3 input online software.
