# Supplementary material for: MT1G induces lipid droplet accumulation through modulation of H3K14 trimethylation accelerating clear cell renal cell carcinoma progression
Source: Br J Cancer. 2024 Jun 21;131(4):641–54. doi: 10.1038/s41416-024-02747-y (PMC11333765; doi:10.1038/s41416-024-02747-y)

Supplementary Fig.2c

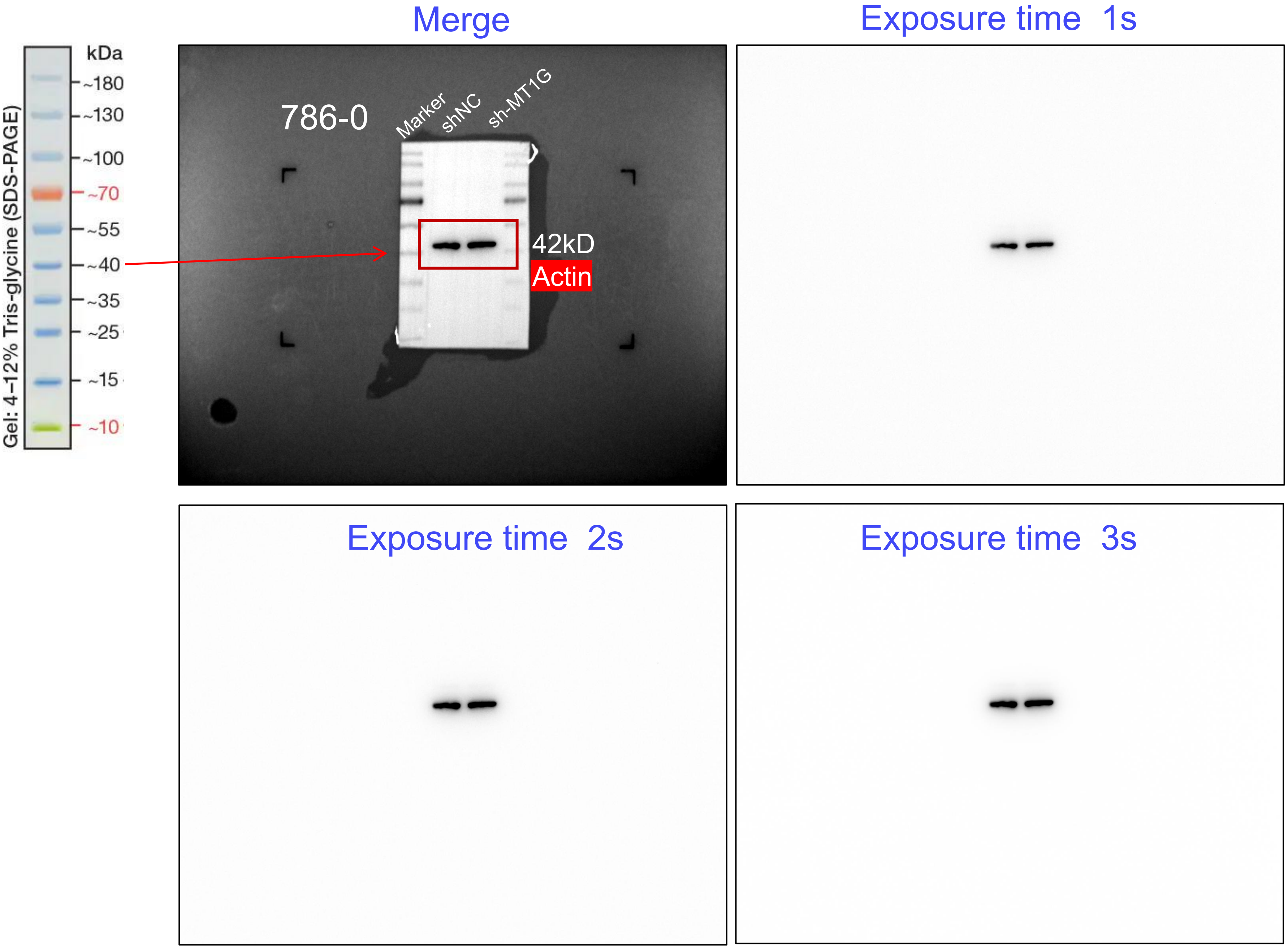

Supplementary Fig.2c

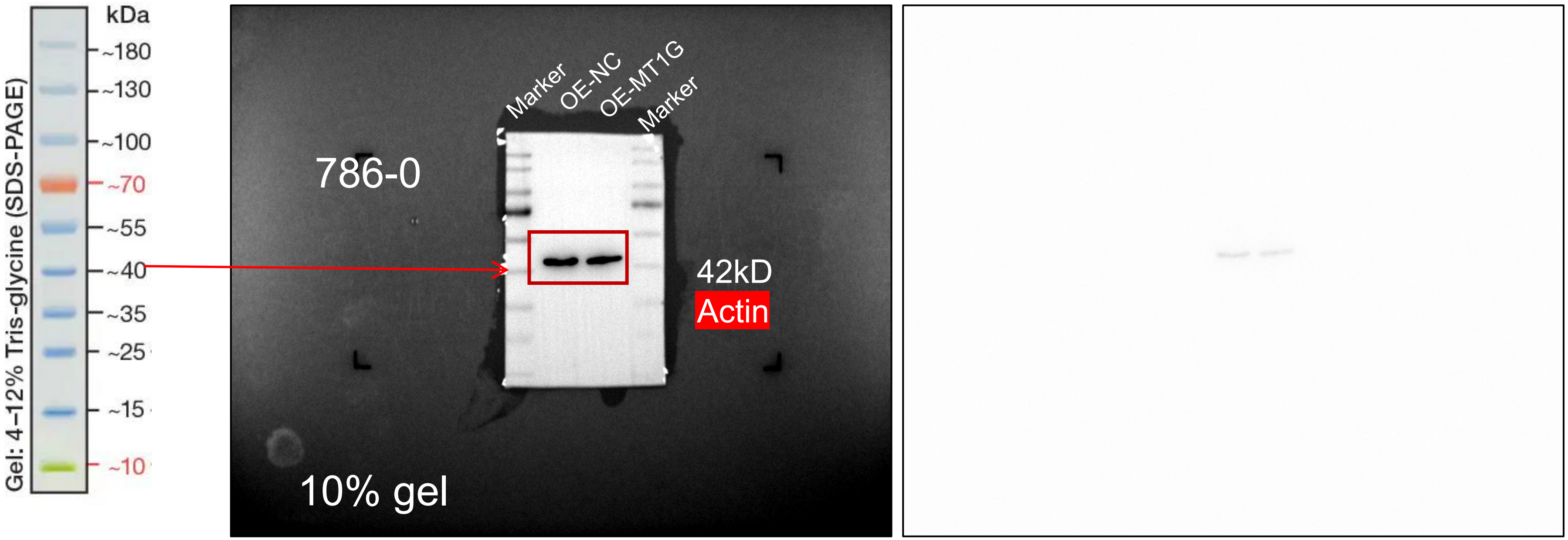

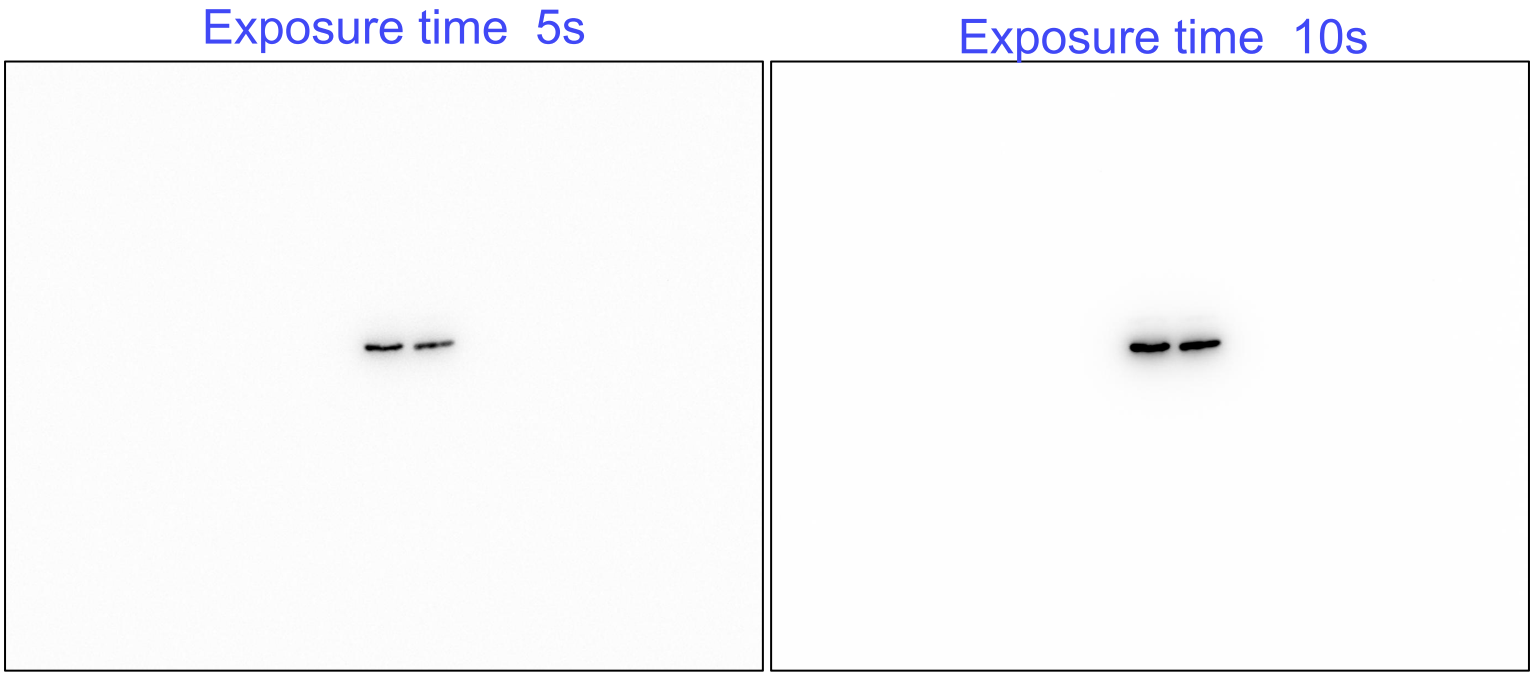

Supplementary Fig.2c

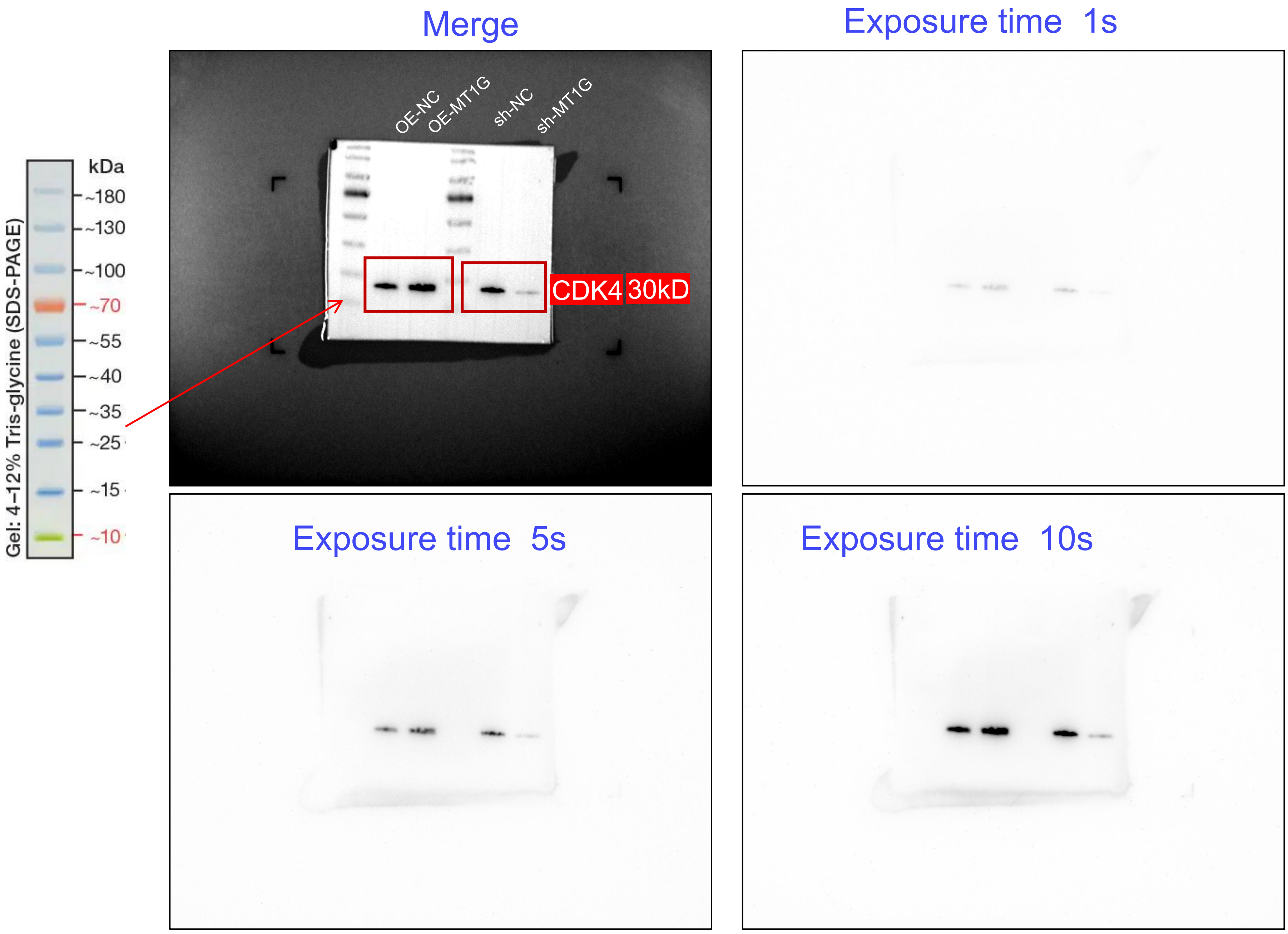

Supplementary Fig.2c

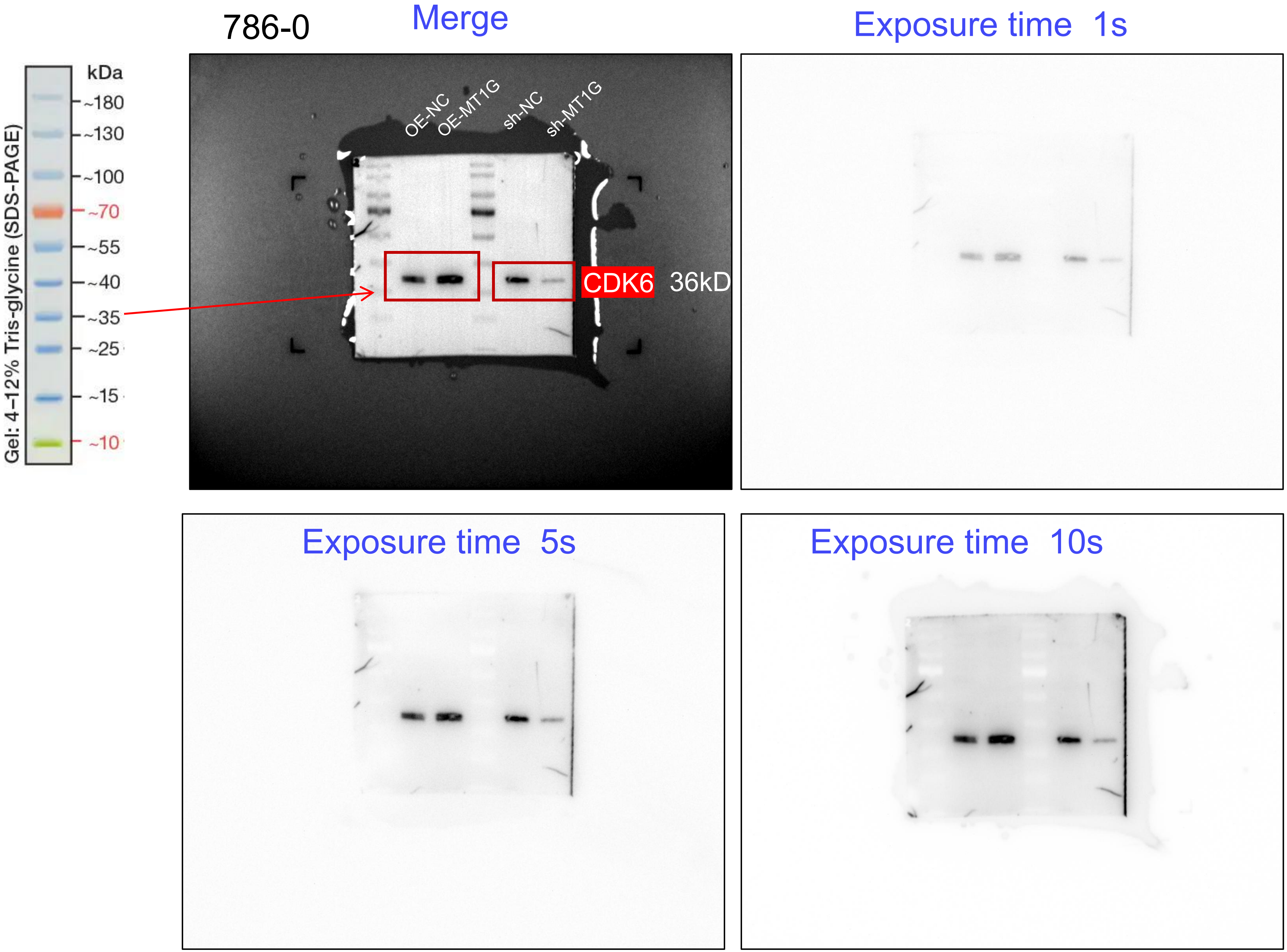

Supplementary Fig.2c

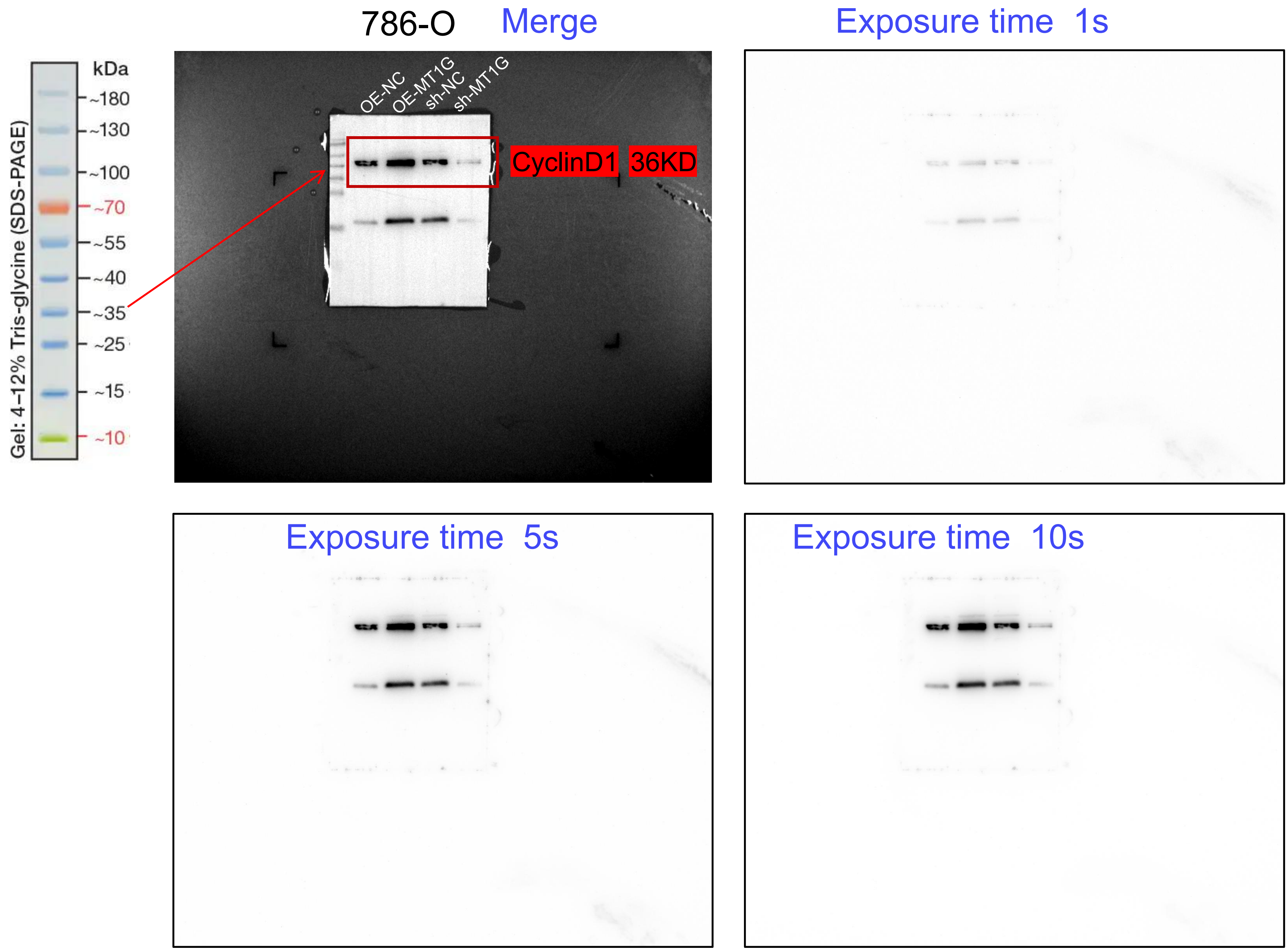

Supplementary Fig.2c

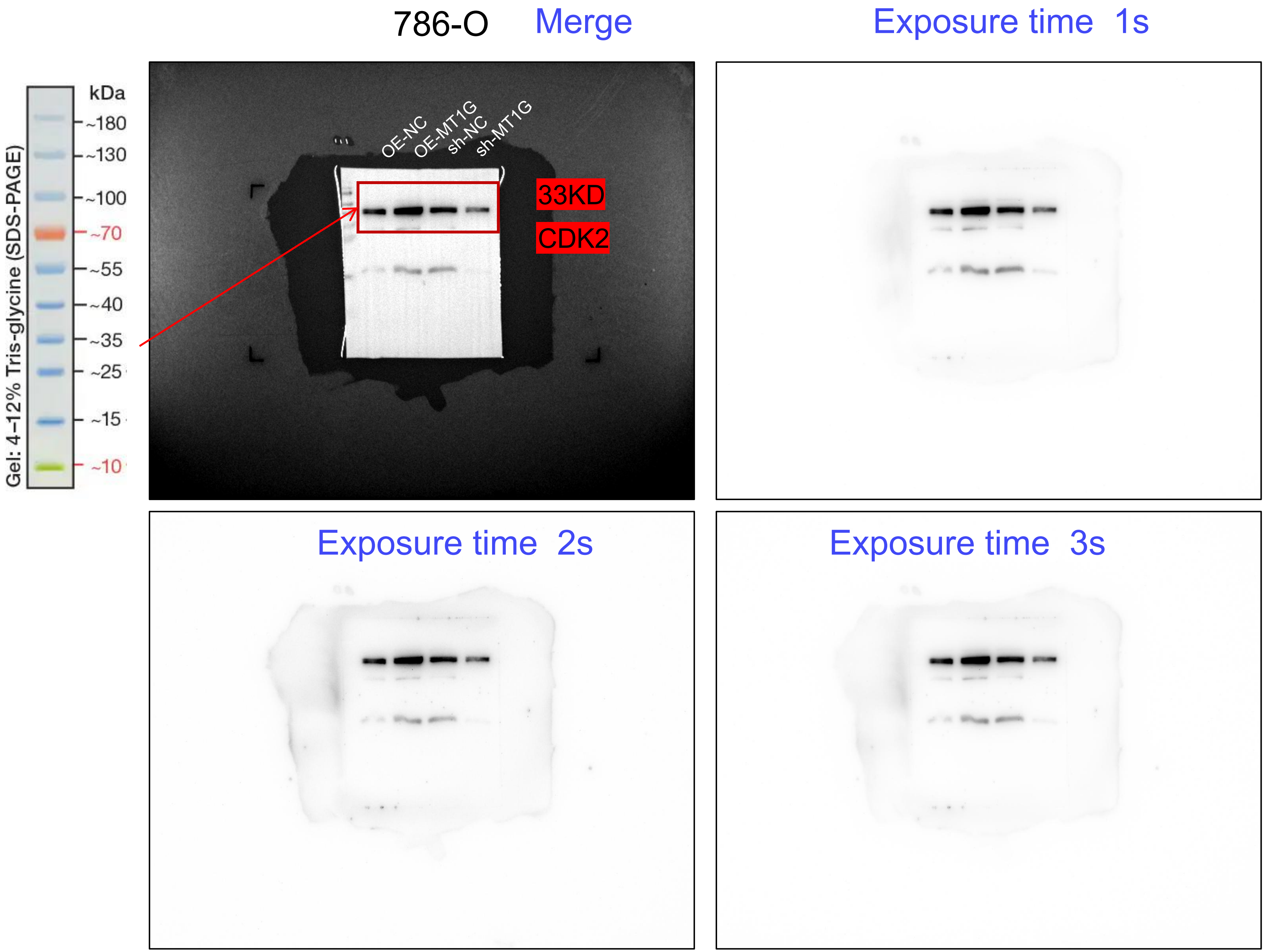

Supplementary Fig.2c

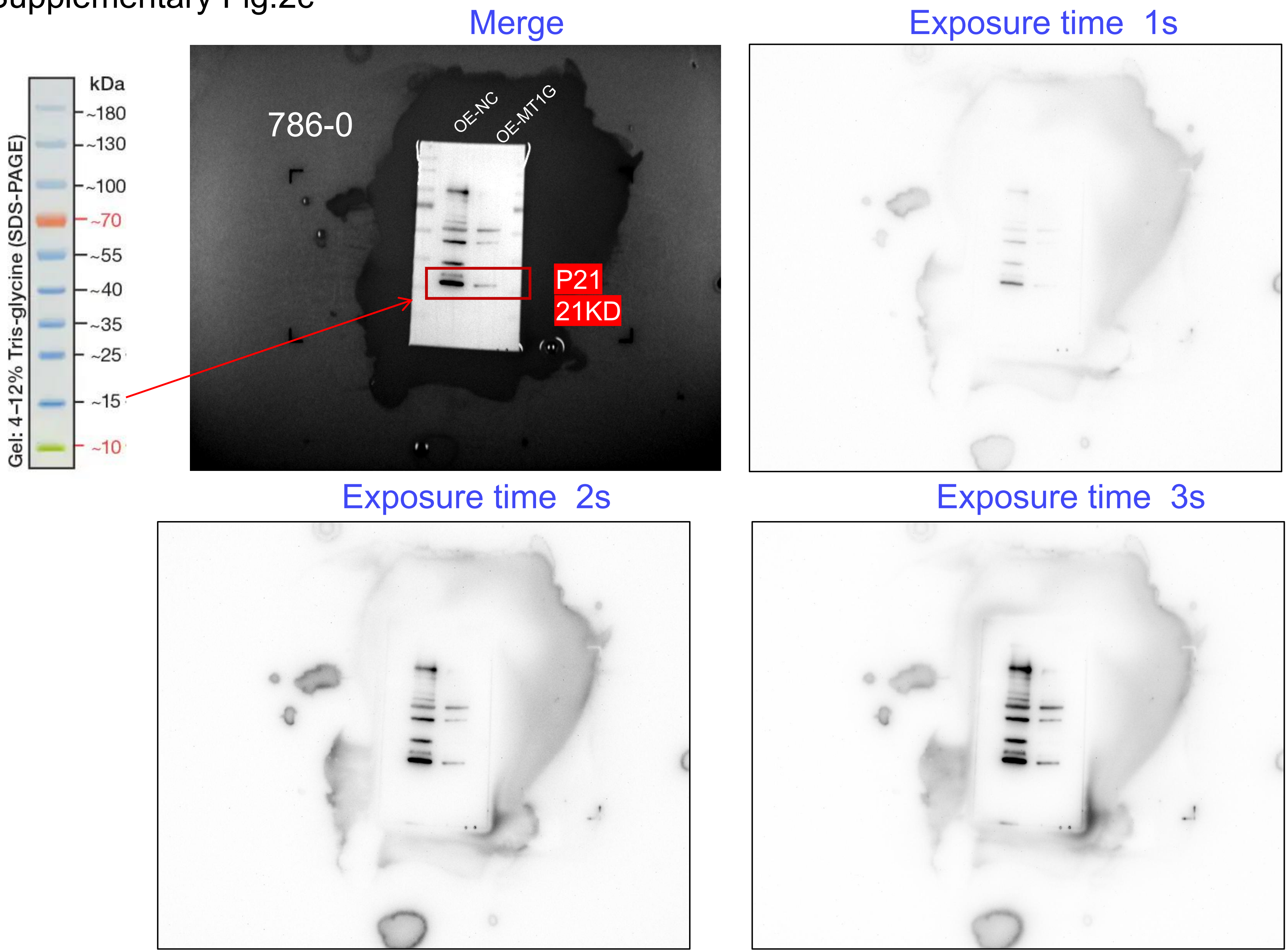

Supplementary Fig.2c

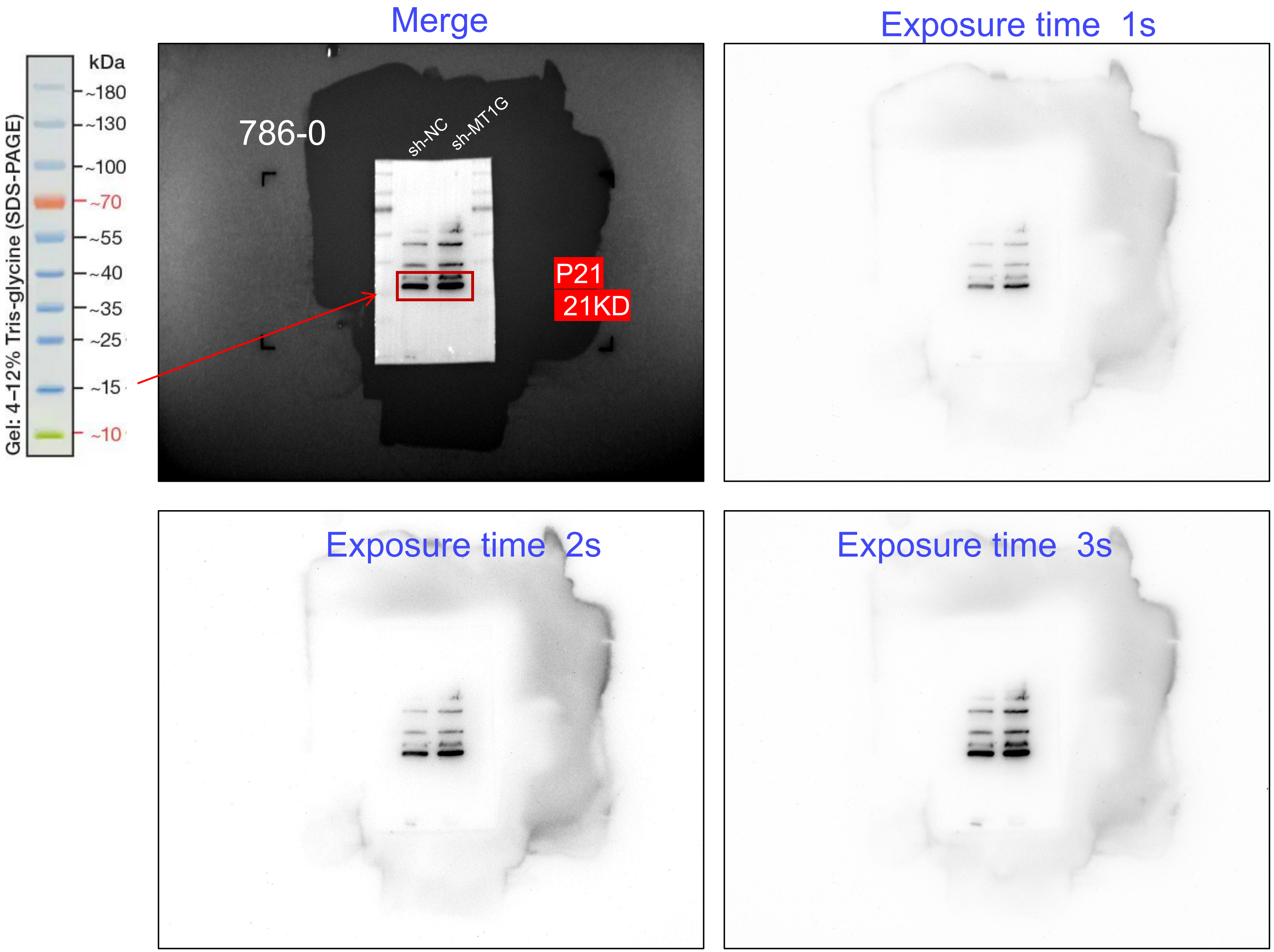

Supplementary Fig.2c

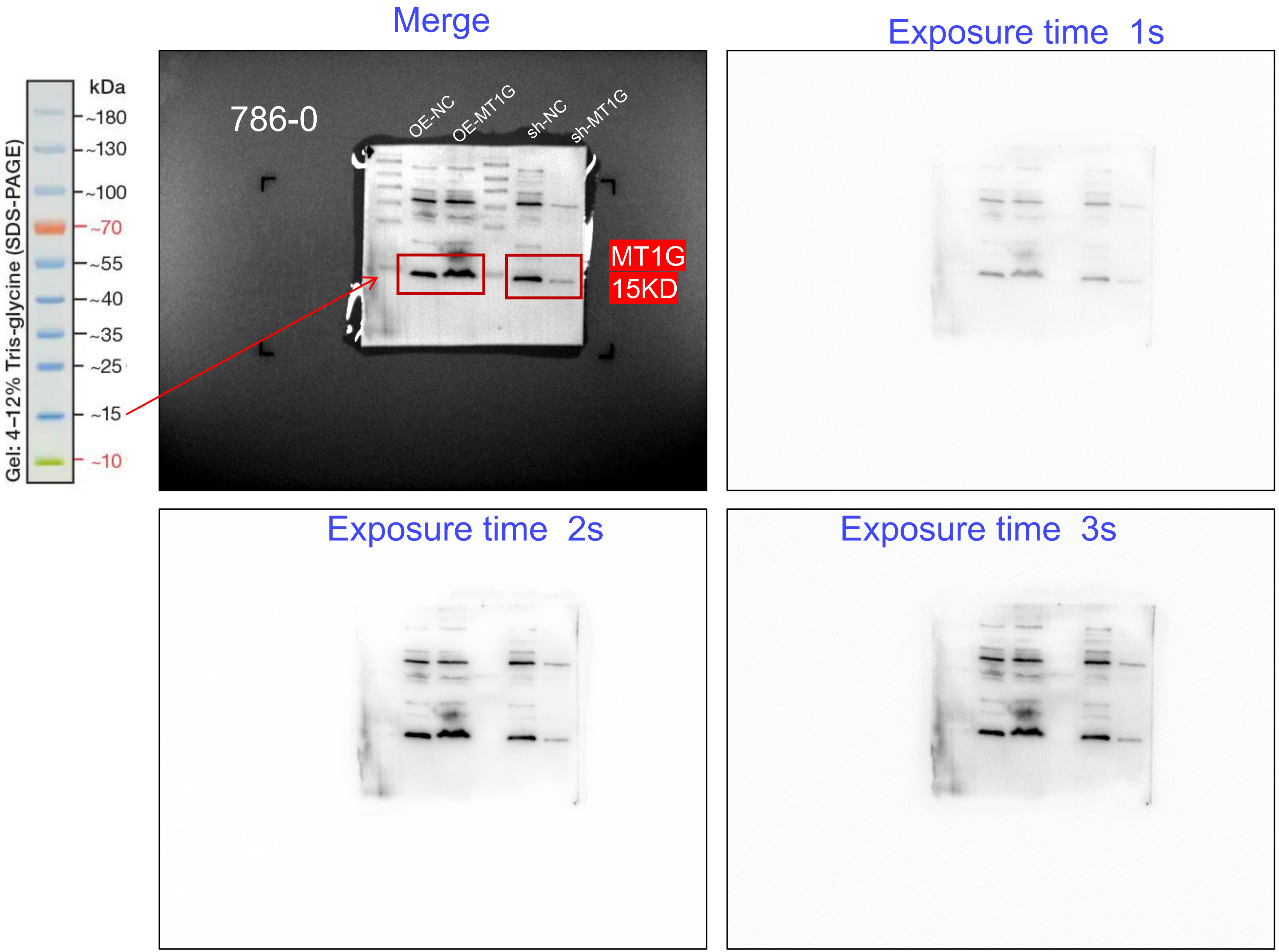

Supplementary Fig.3I

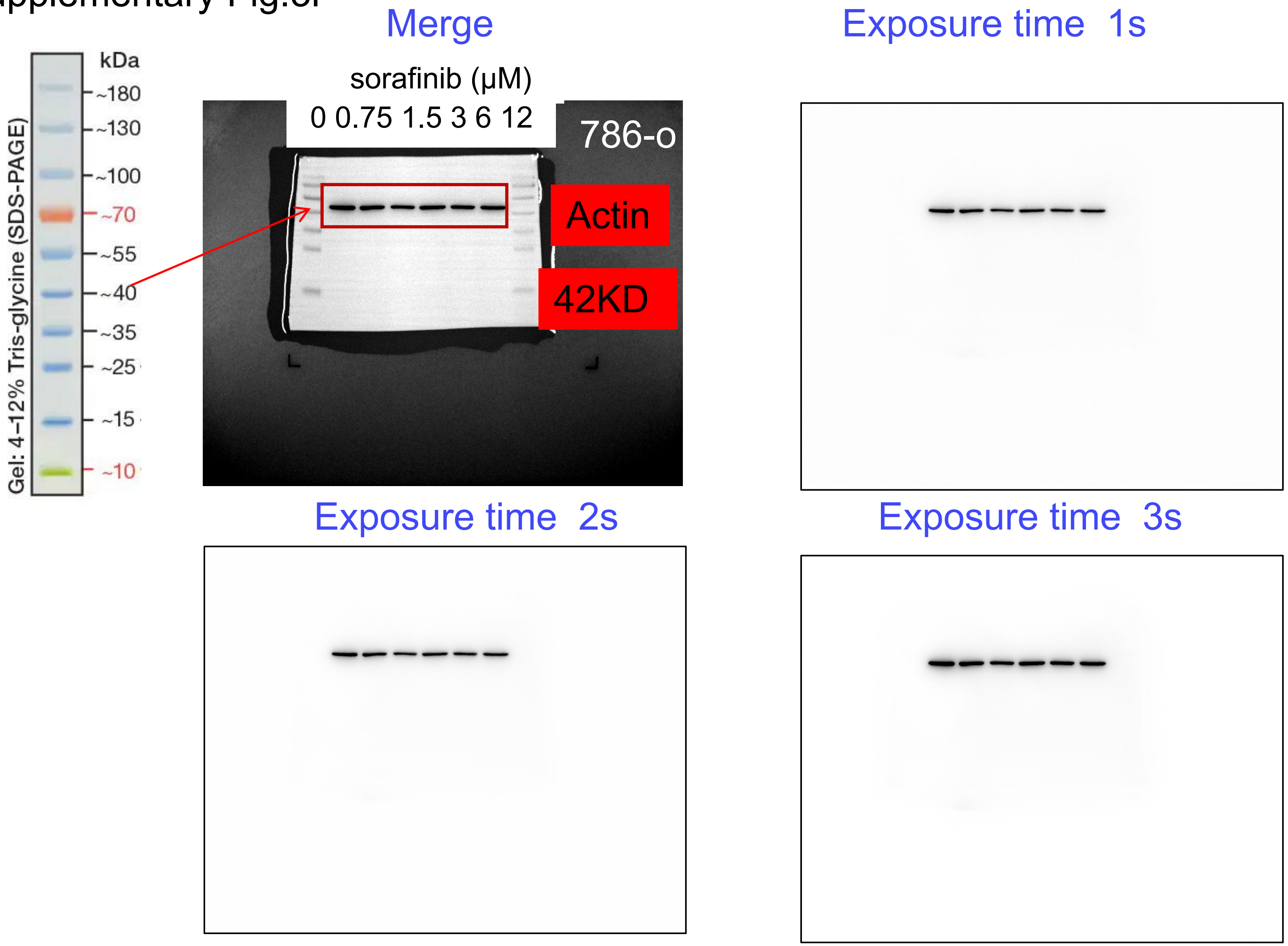

Supplementary Fig.3I

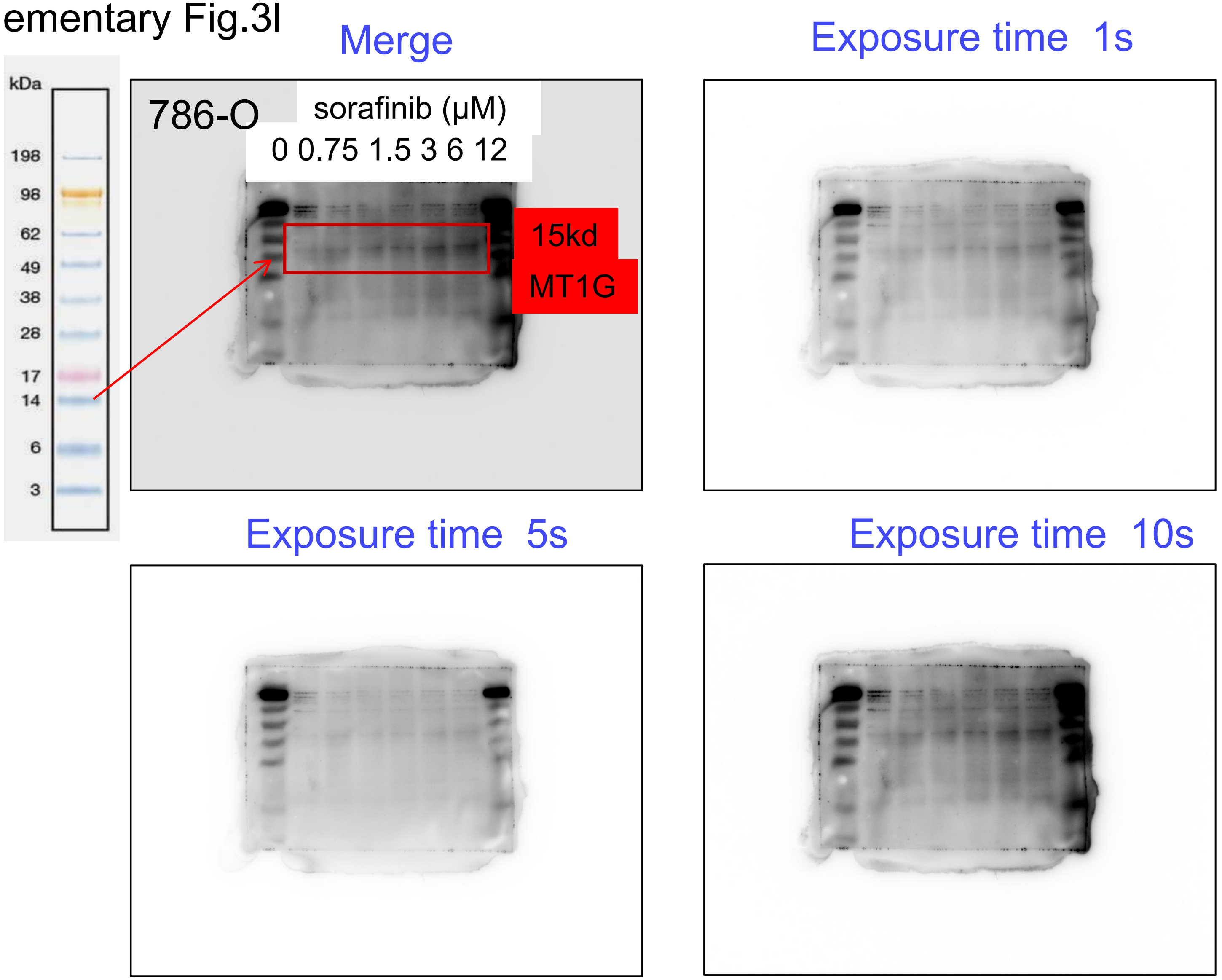

Supplementary Fig.3q

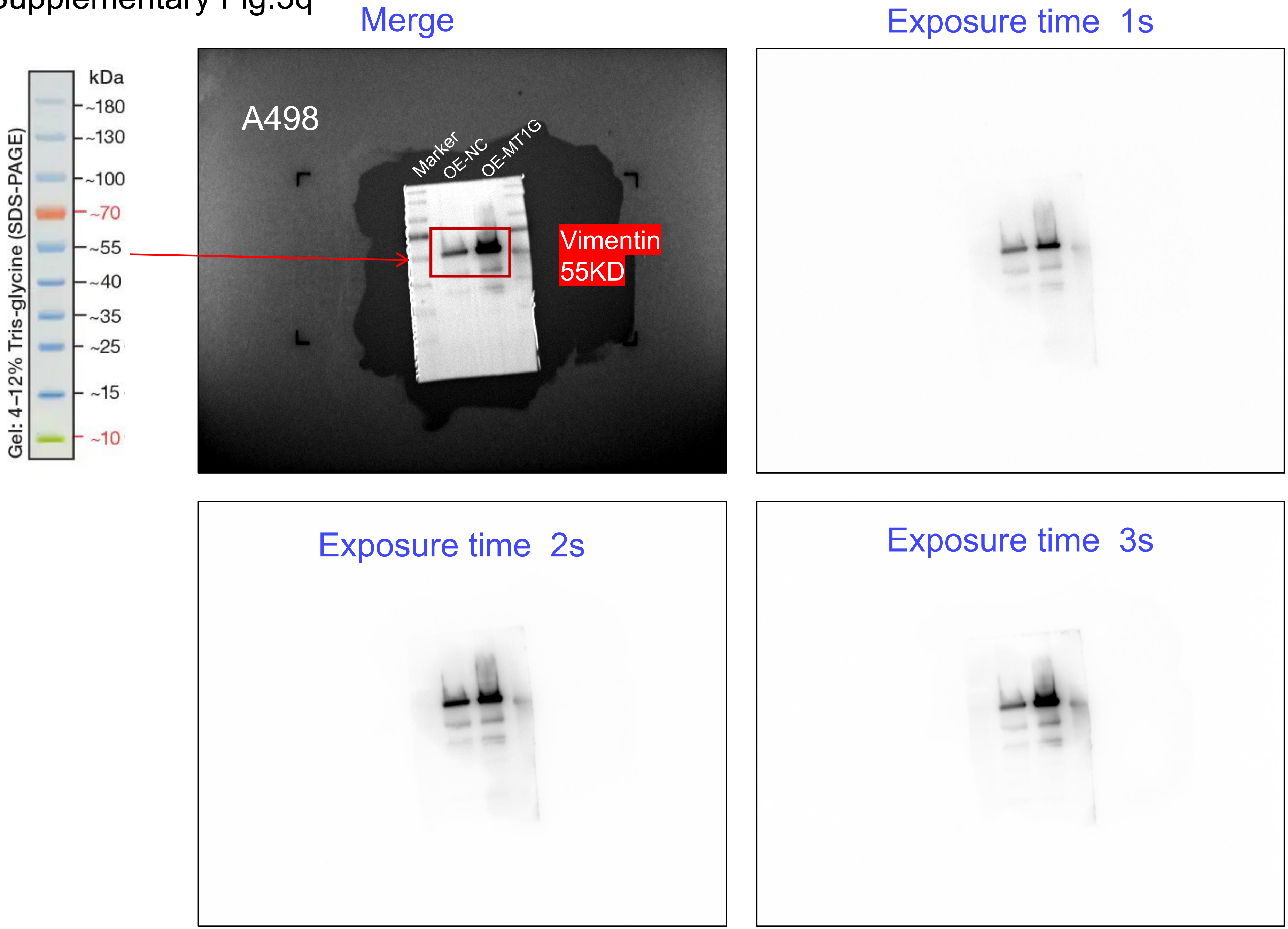

Supplementary Fig.3q

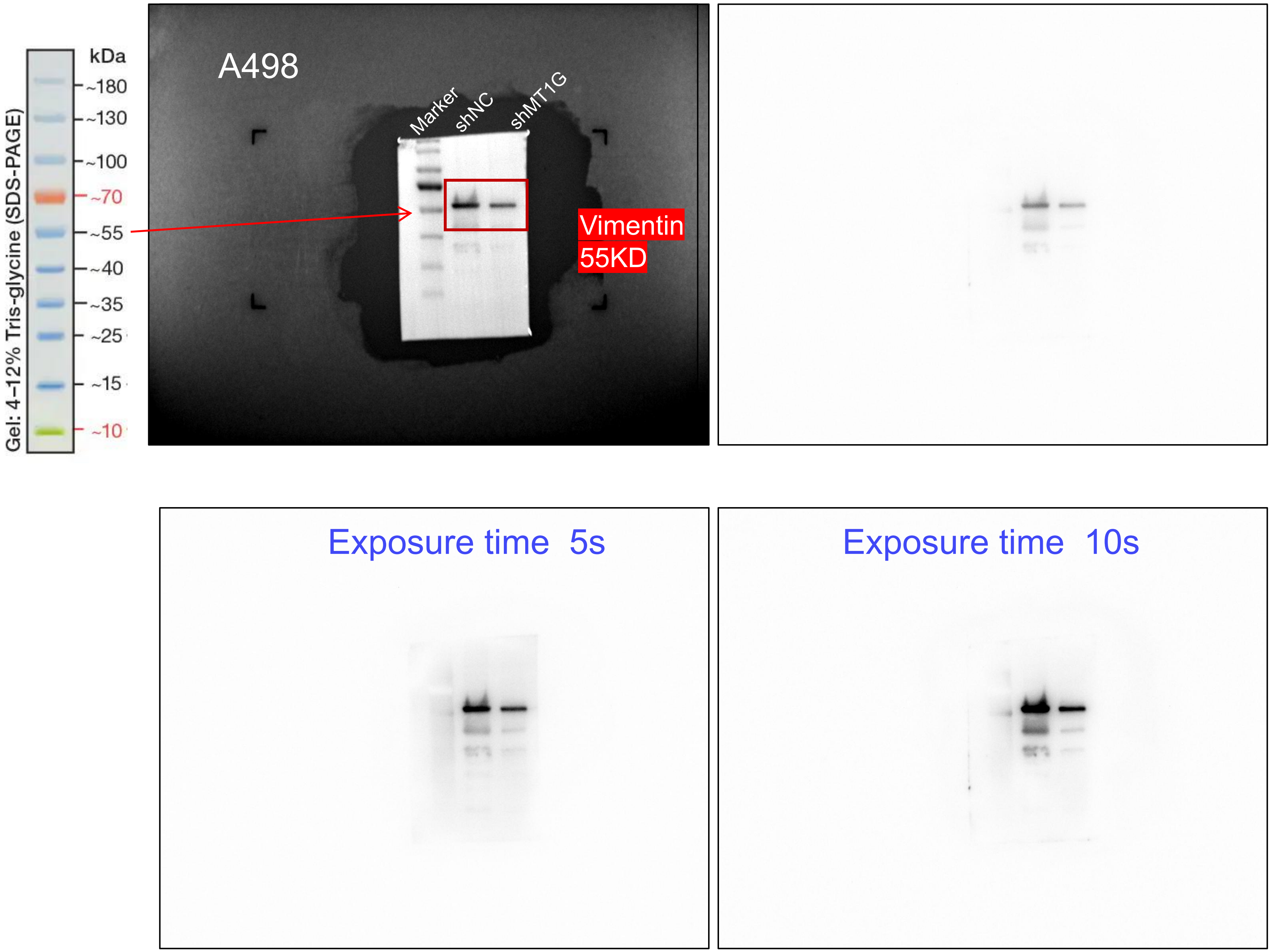

Supplementary Fig.3q

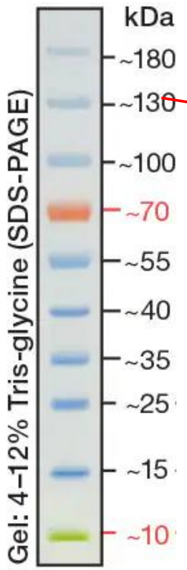

Merge

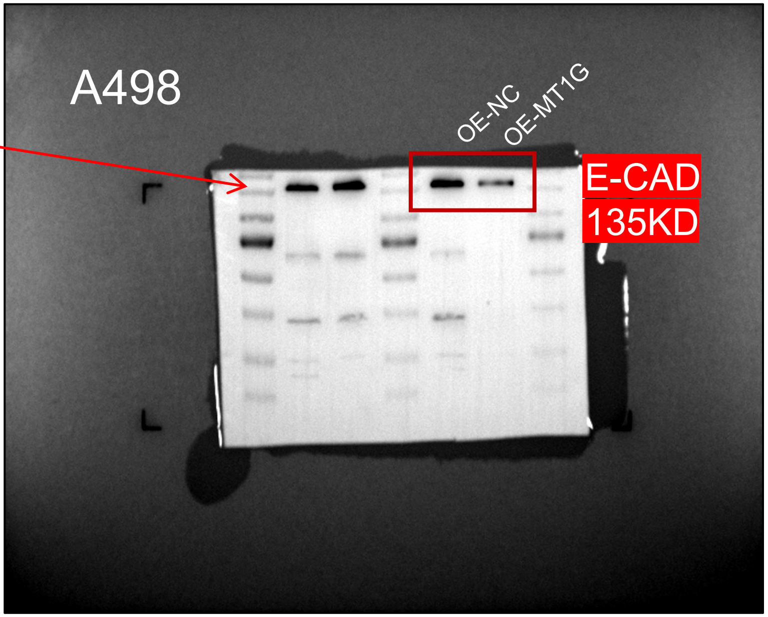

Exposure time 1s

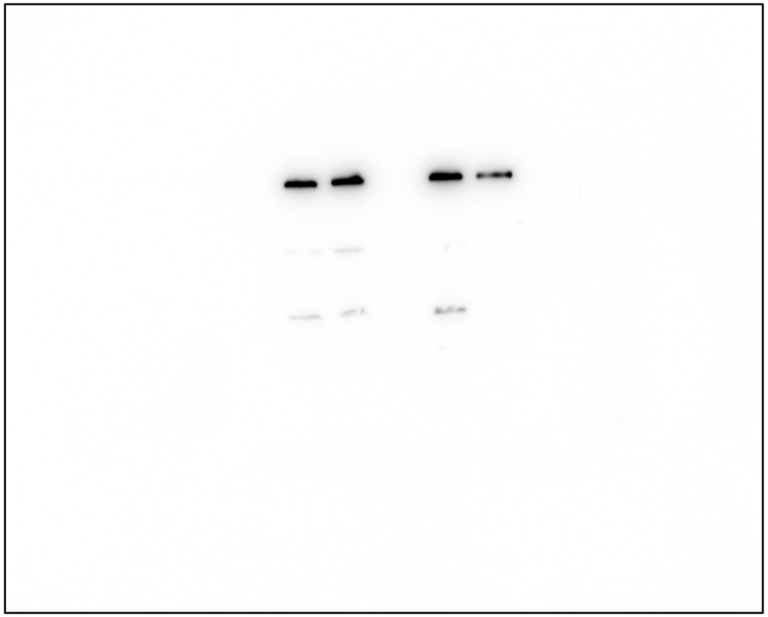

Exposure time 2s

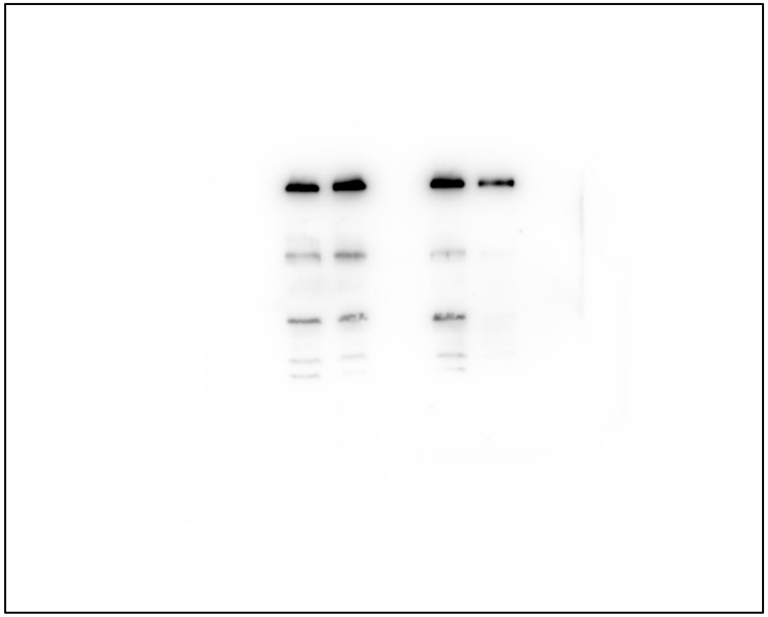

Exposure time 3s

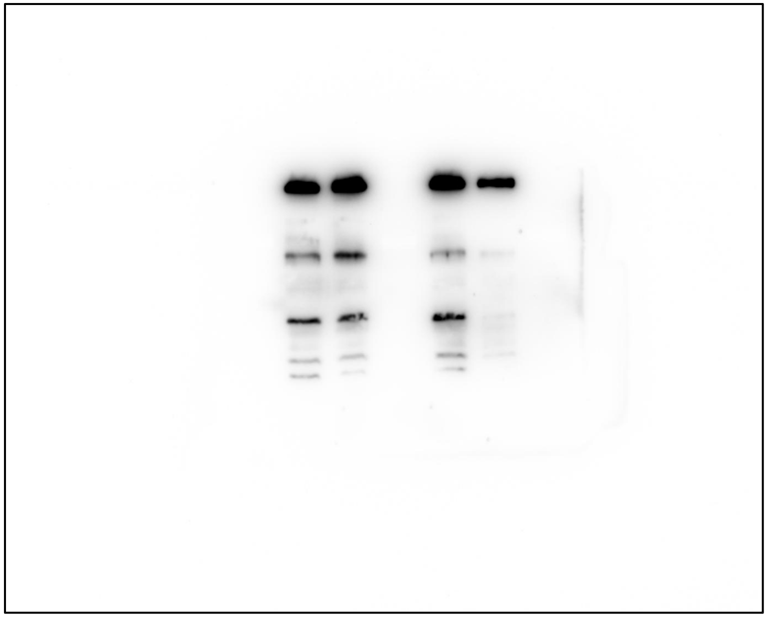

Supplementary Fig.3q

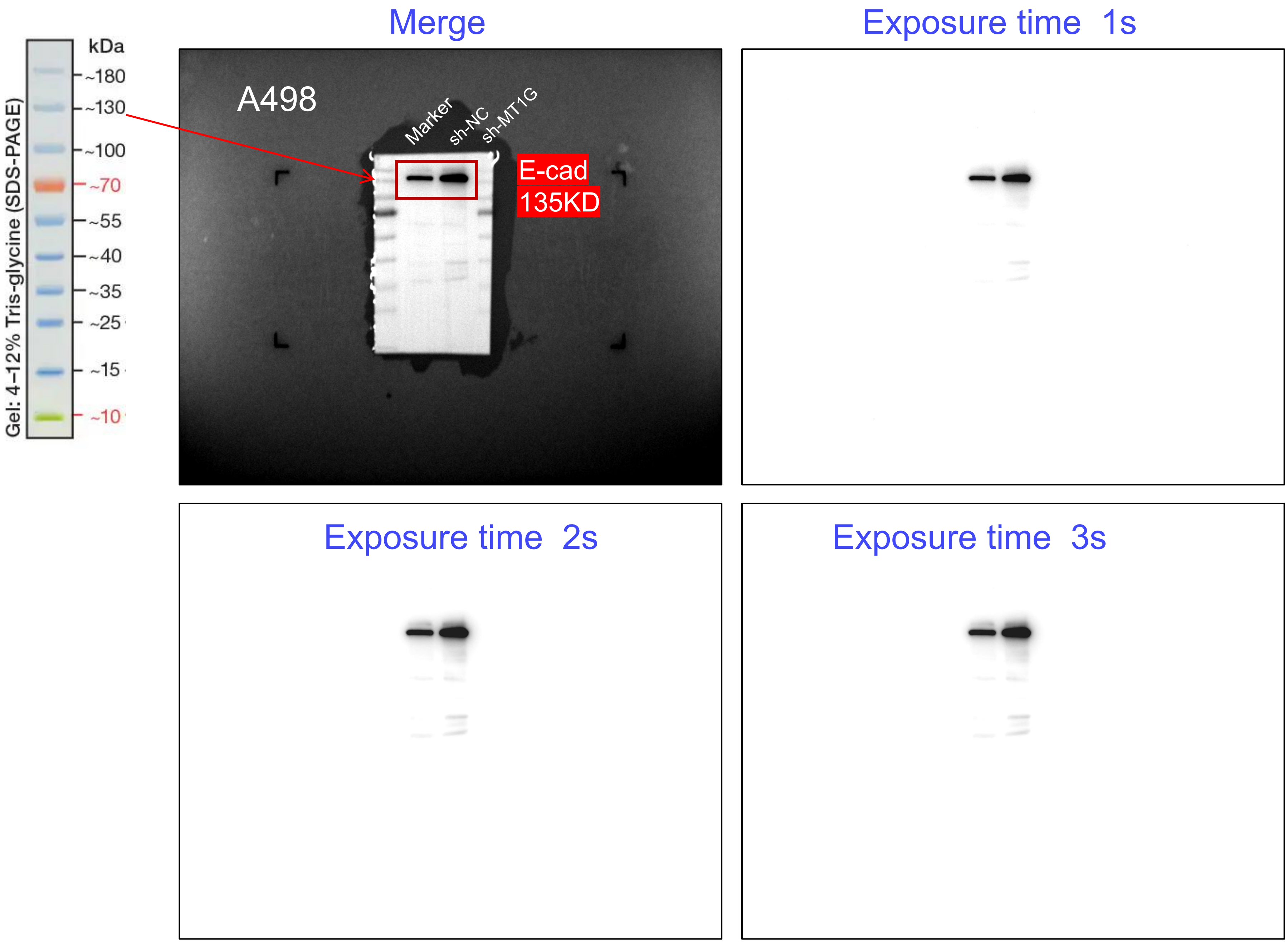

Supplementary Fig.3q

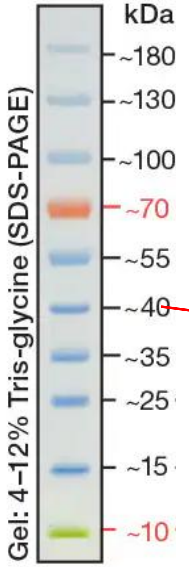

Merge

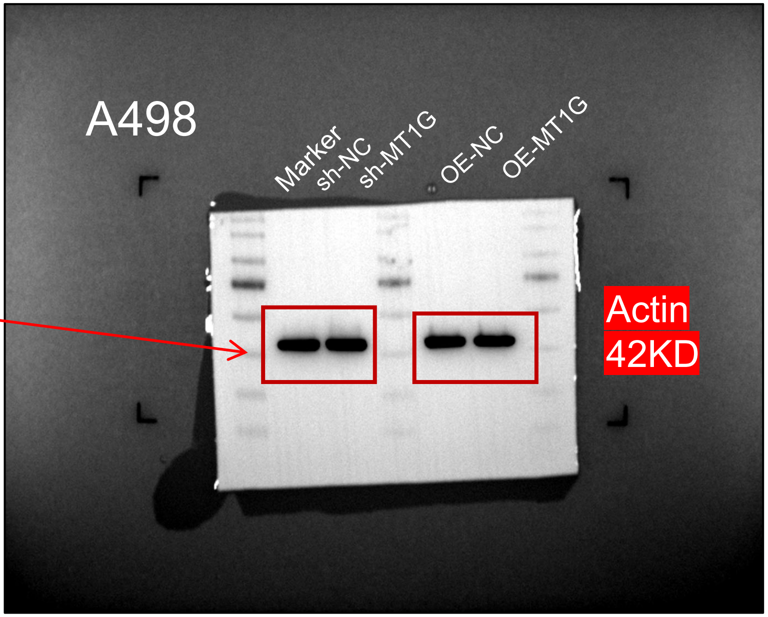

Exposure time 1s

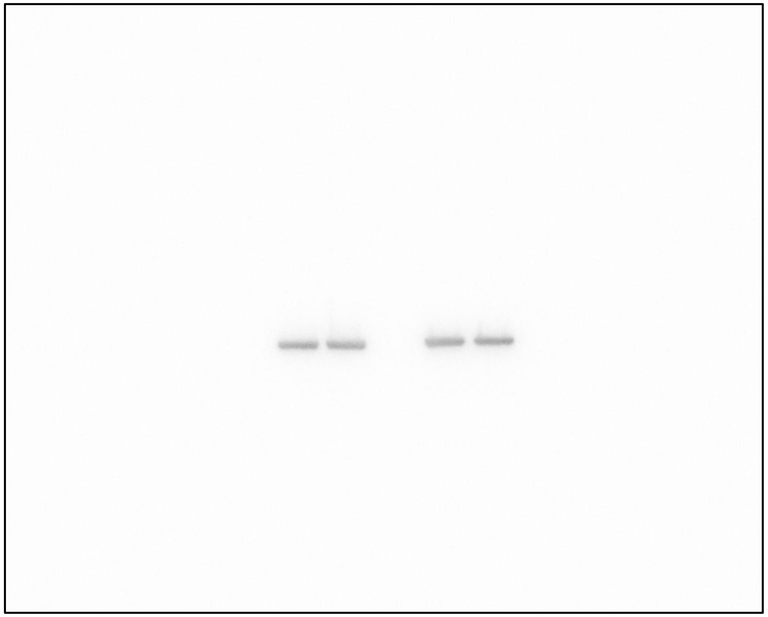

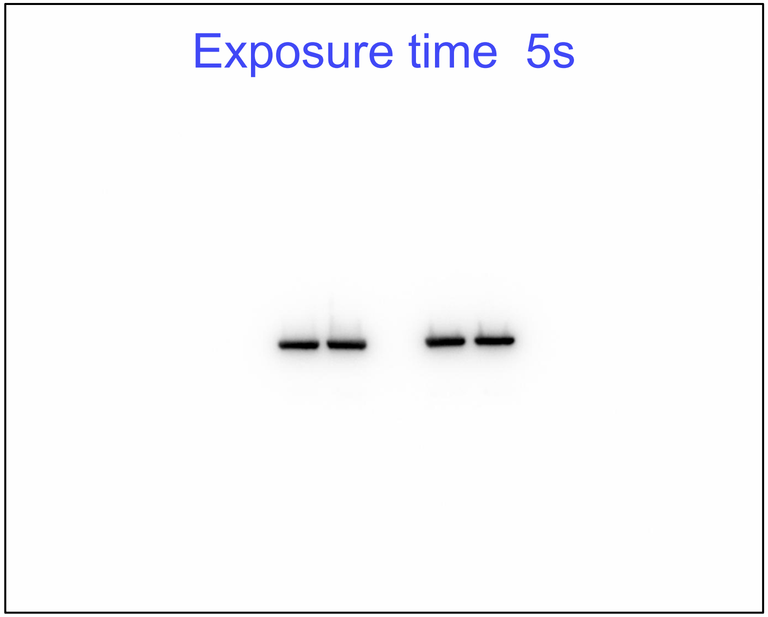

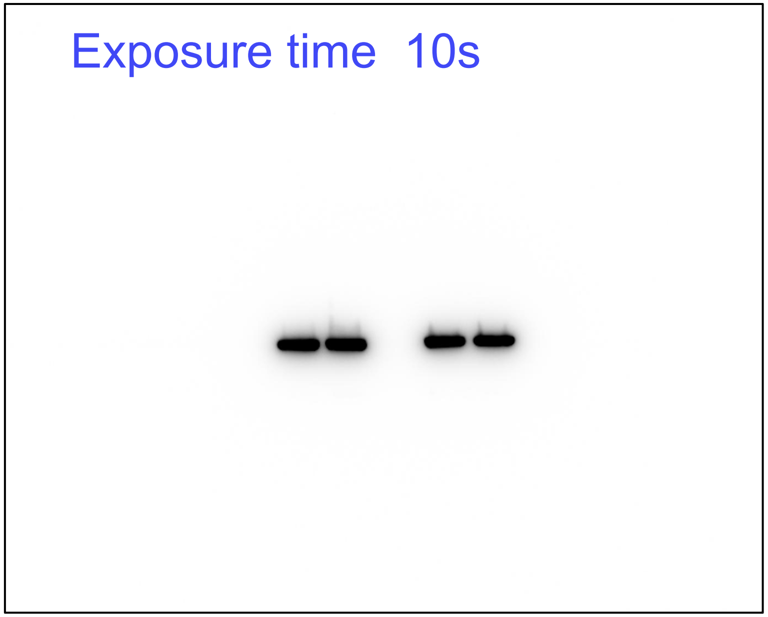

Supplementary Fig.3q

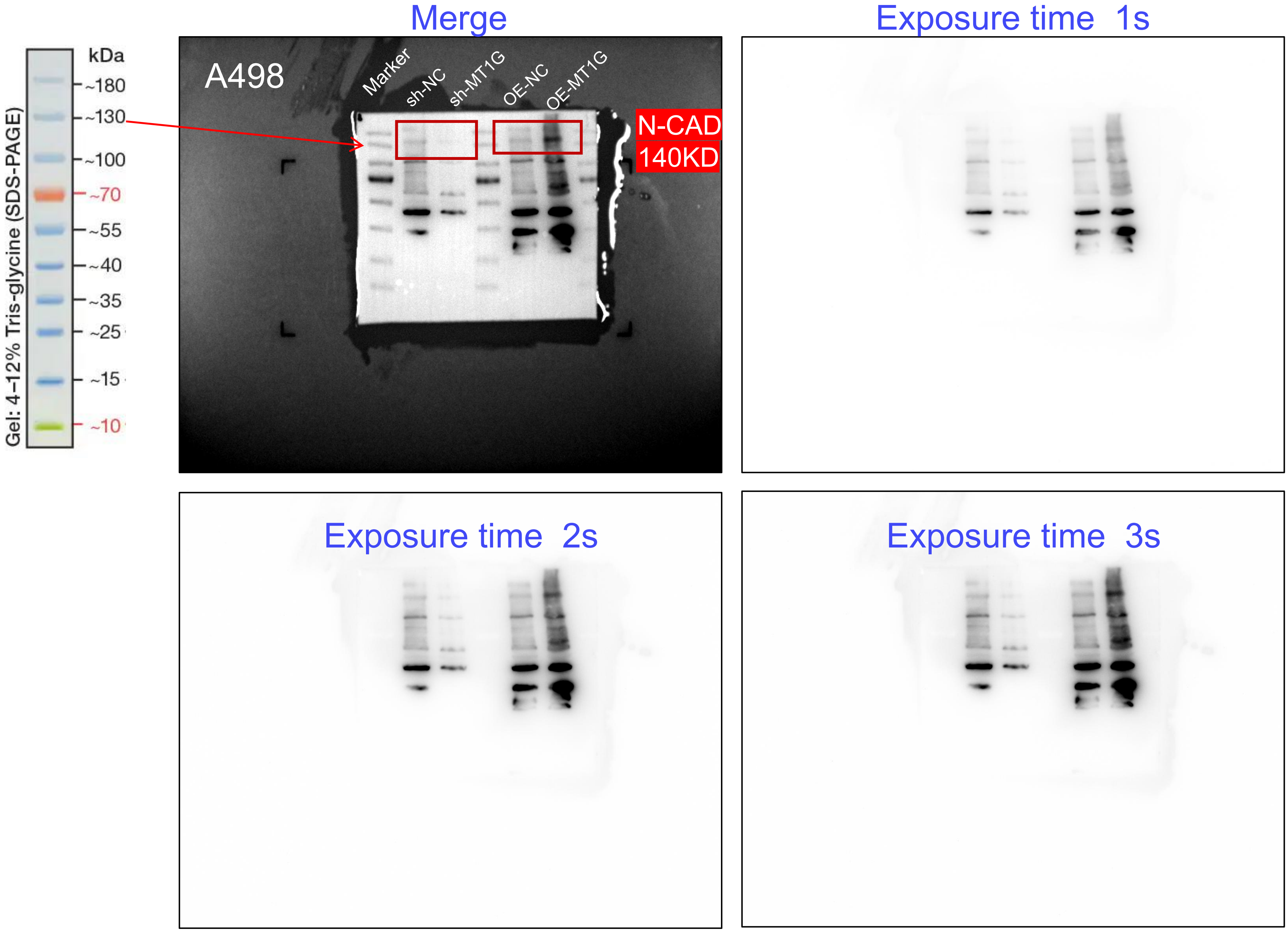

Supplementary Fig.3q

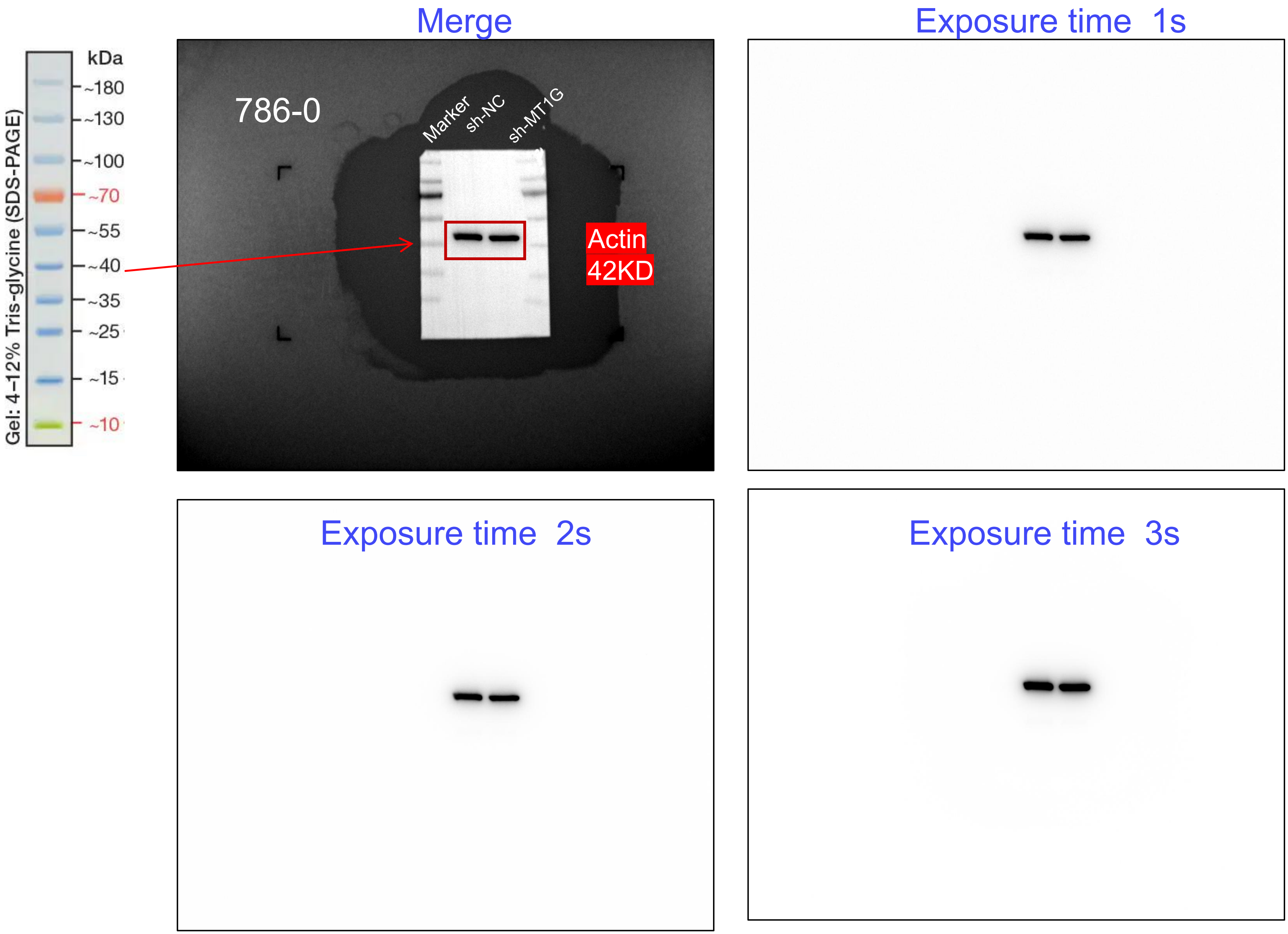

Supplementary Fig.3q

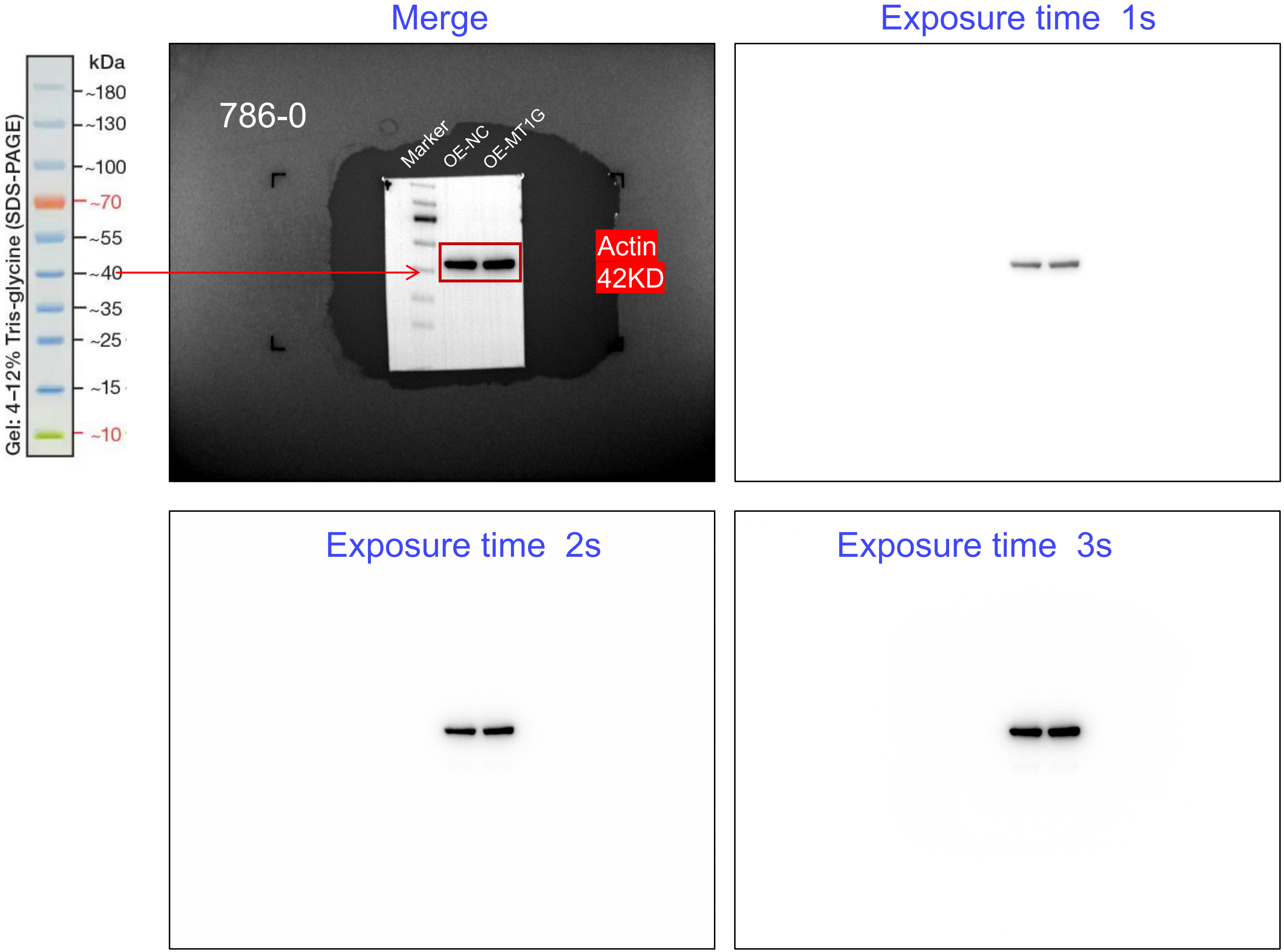

Supplementary Fig.3q

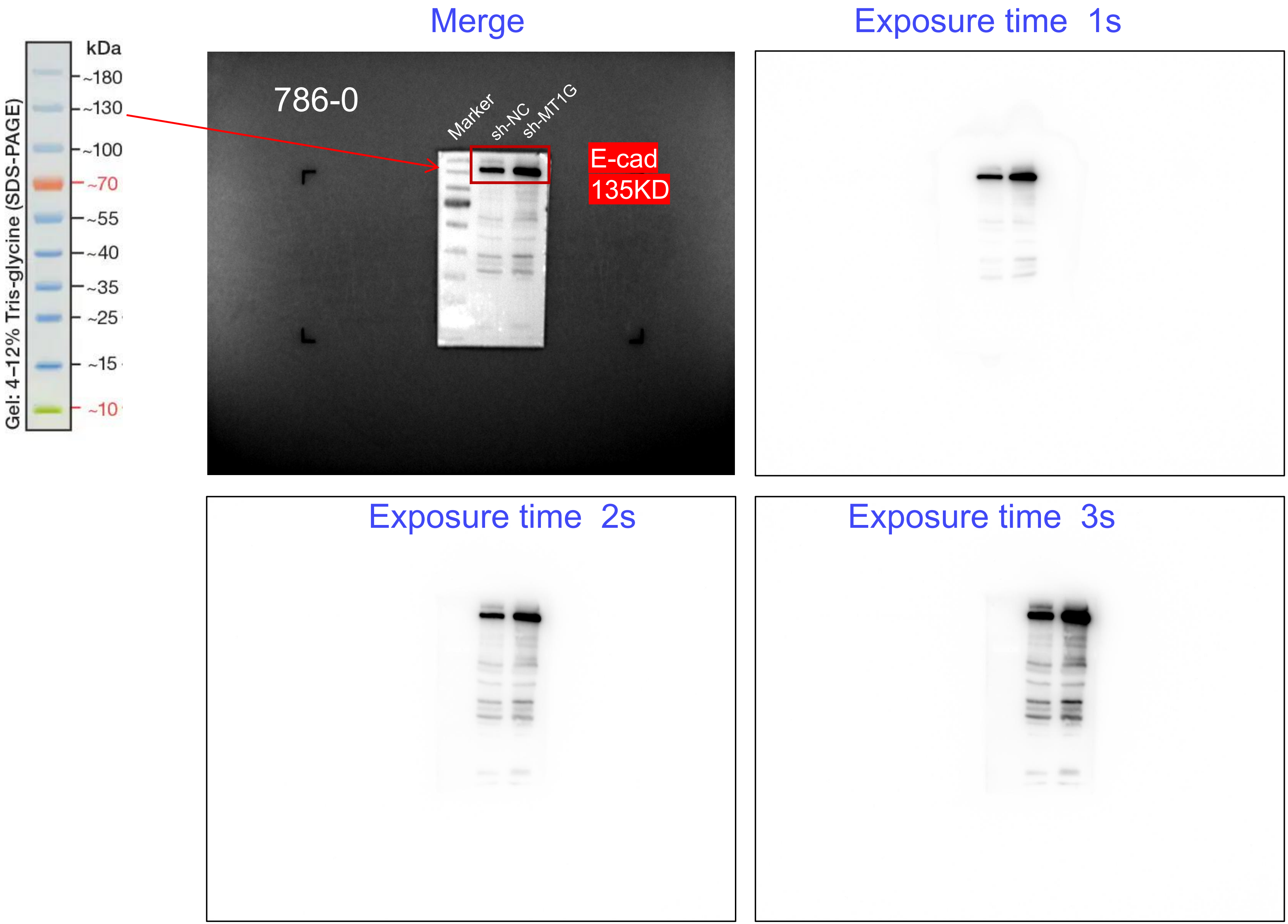

Supplementary Fig.3q

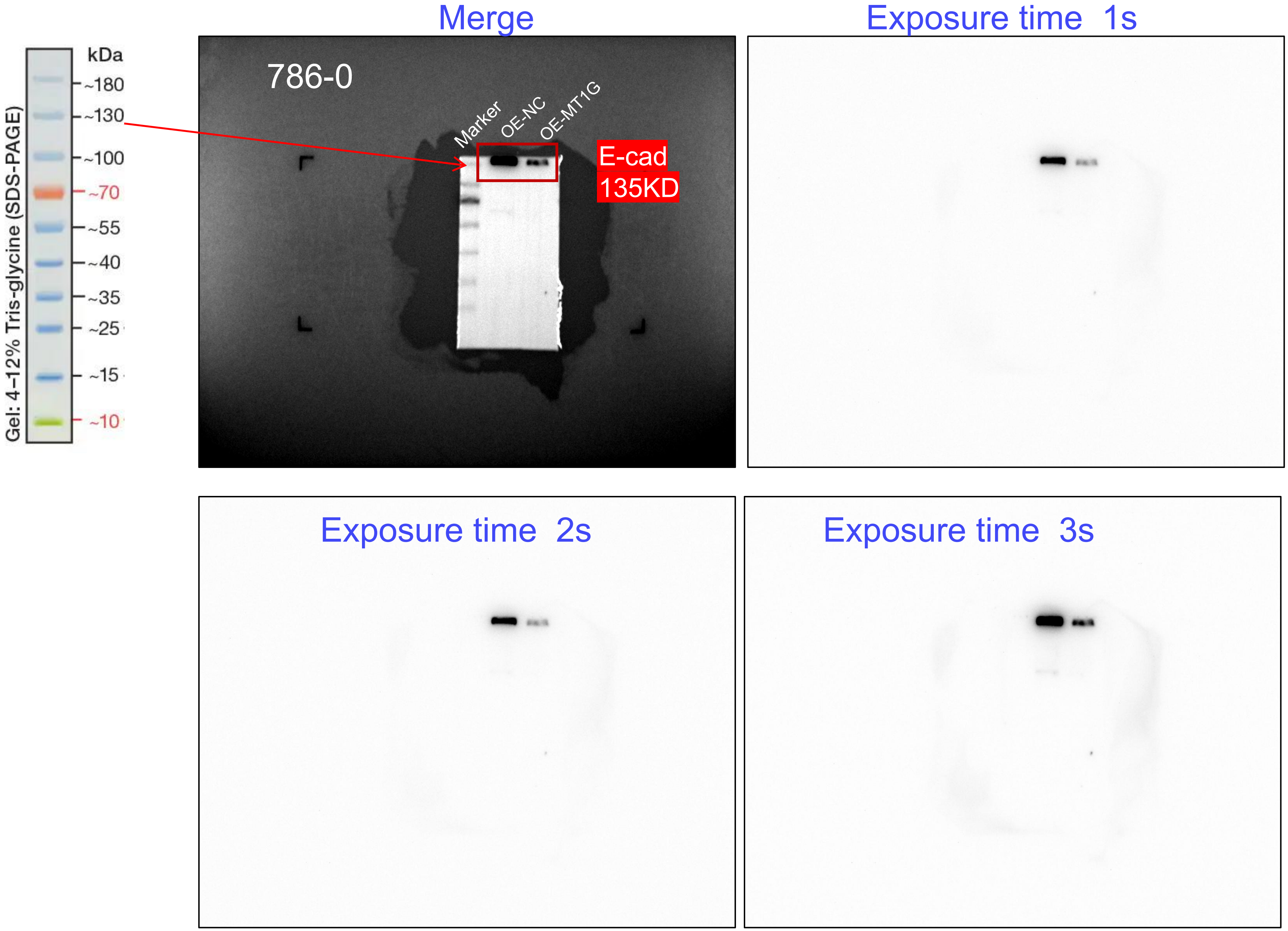

Supplementary Fig.3q

Merge

Exposure time 1s

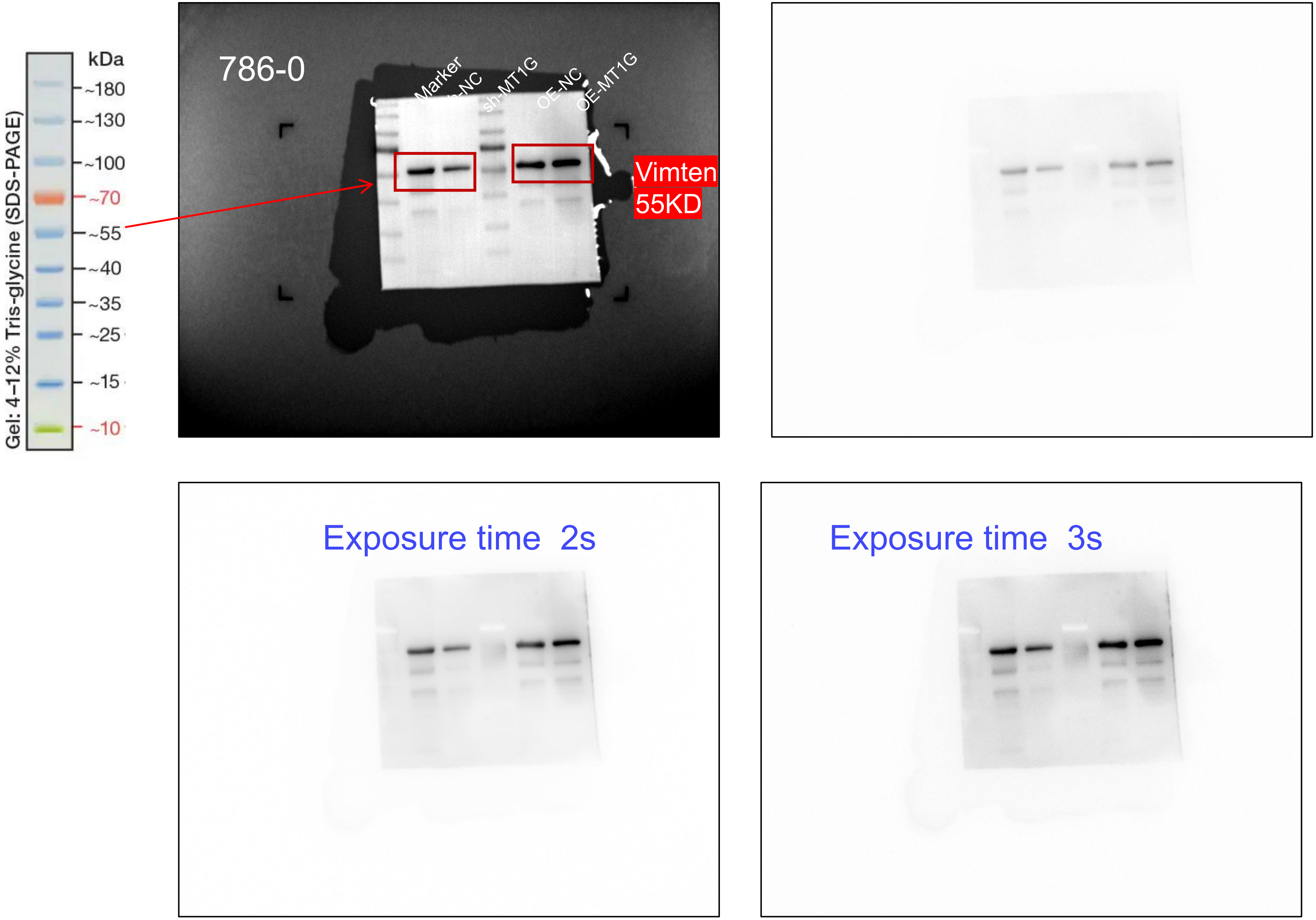

Supplementary Fig.3q

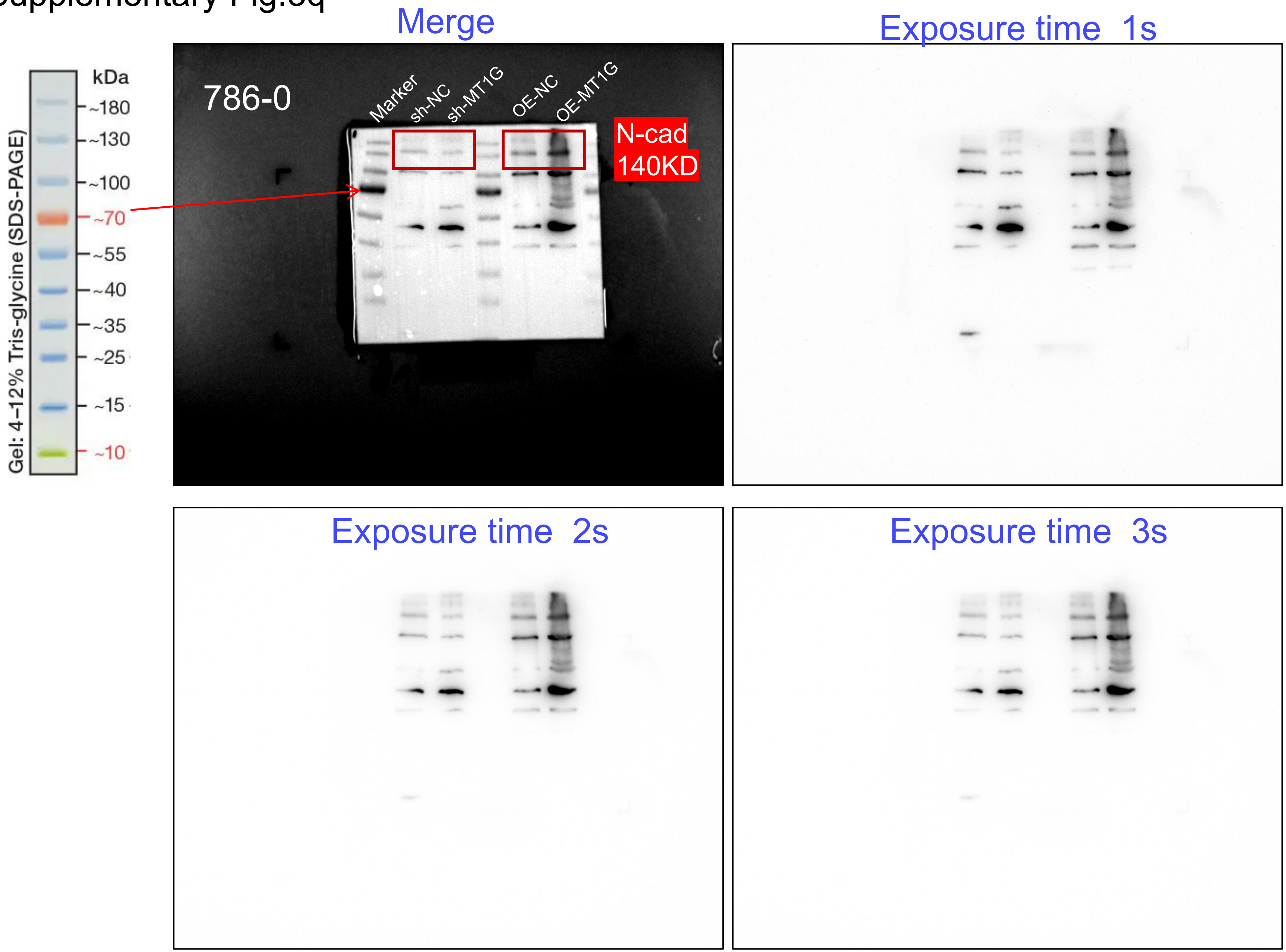

Fig.5 d

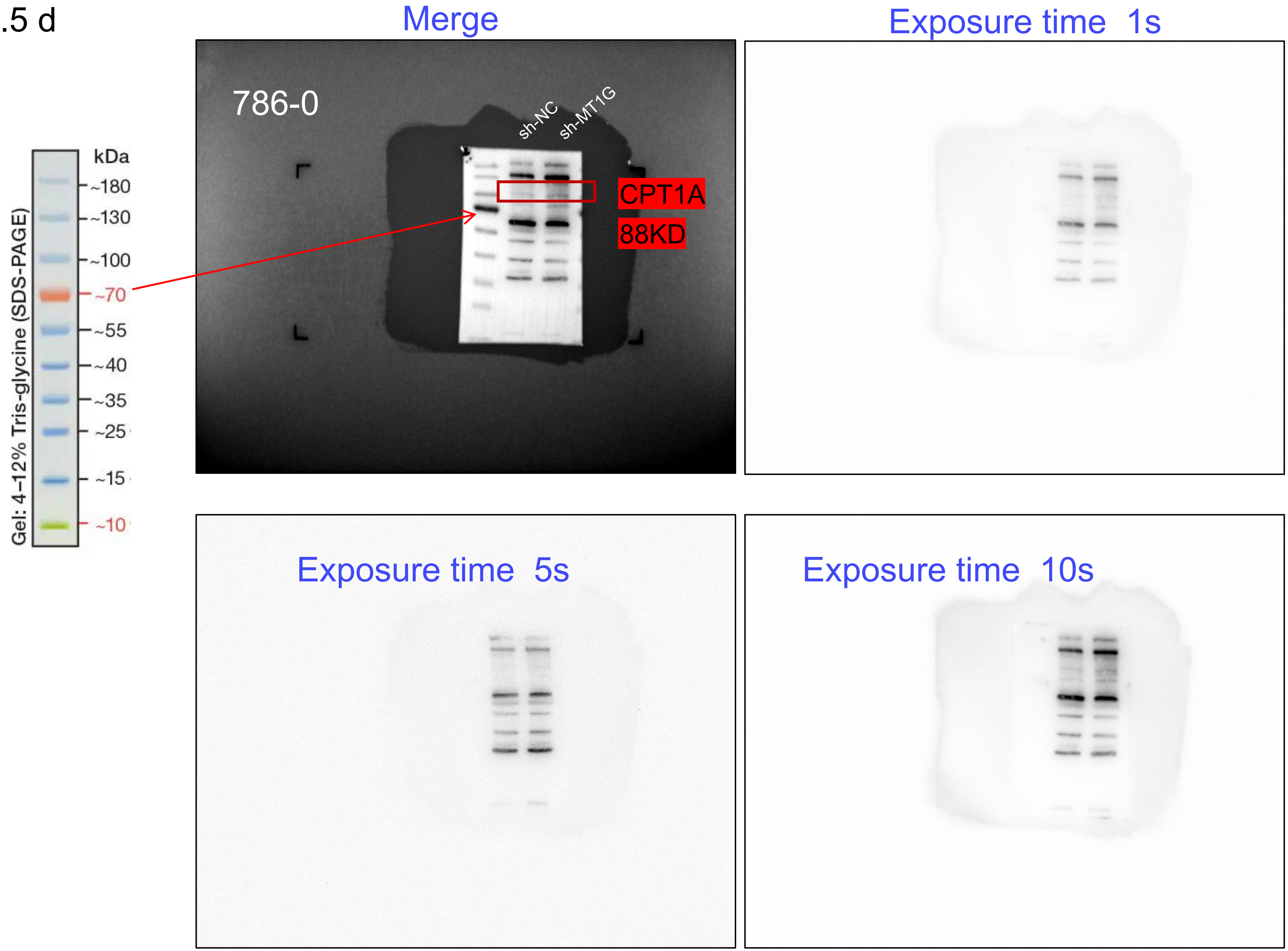
Merge
Exposure time 1s
786-0
sh-NC
sh-MT1G
CPT1A
88KD
kDa
~180
~130
~100
~70
~55
~40
~35
~25
~15
~10
Gel: 4–12% Tris-glycine (SDS-PAGE)
Exposure time 5s
Exposure time 10s

Fig.5 d

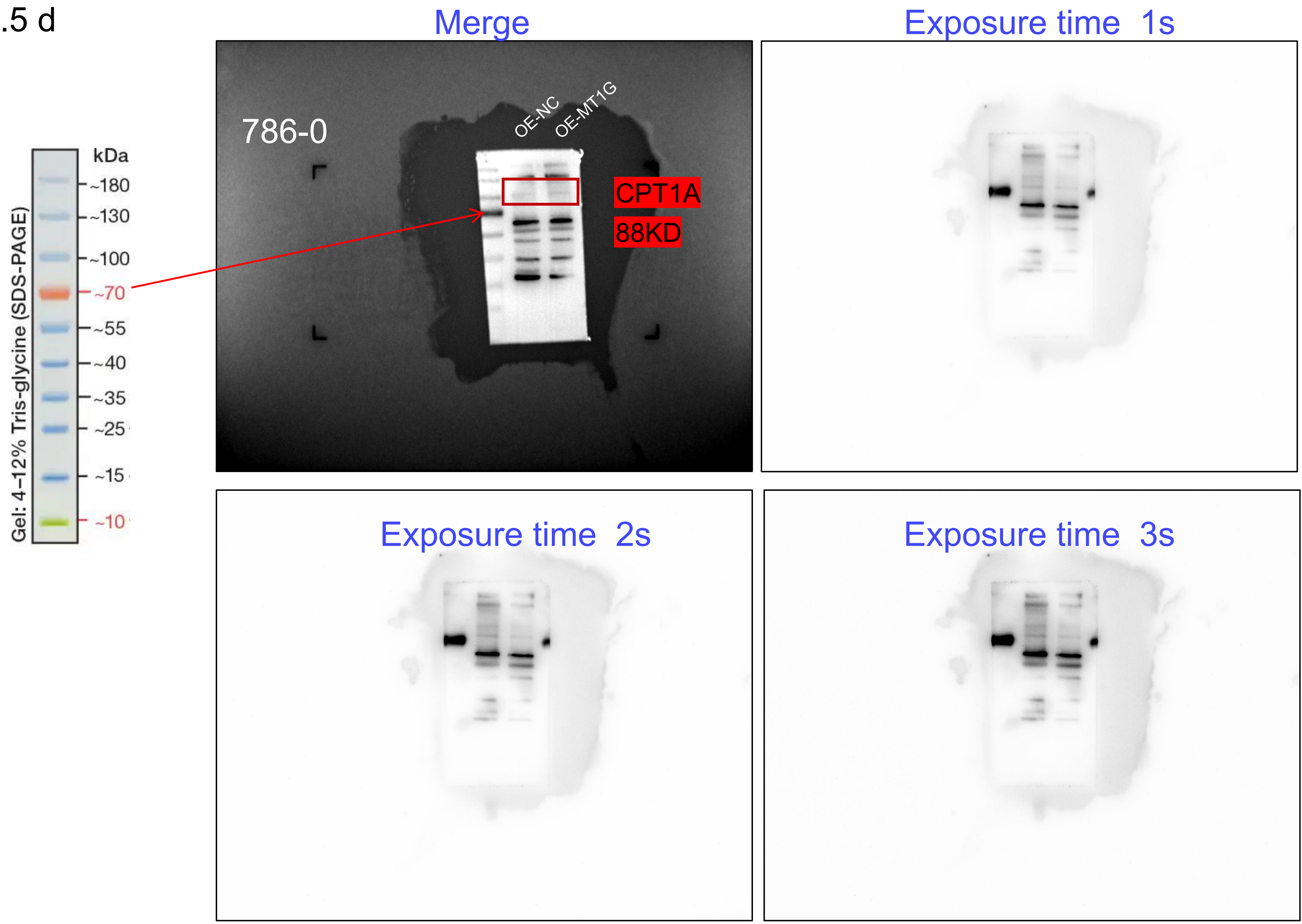
kDa
~180
~130
~100
~70
~55
~40
~35
~25
~15
~10
Gel: 4–12% Tris-glycine (SDS-PAGE)
Merge
786-0
OE-NC
OE-MT1G
CPT1A
88KD
Exposure time 1s
Exposure time 2s
Exposure time 3s

Fig.5 d

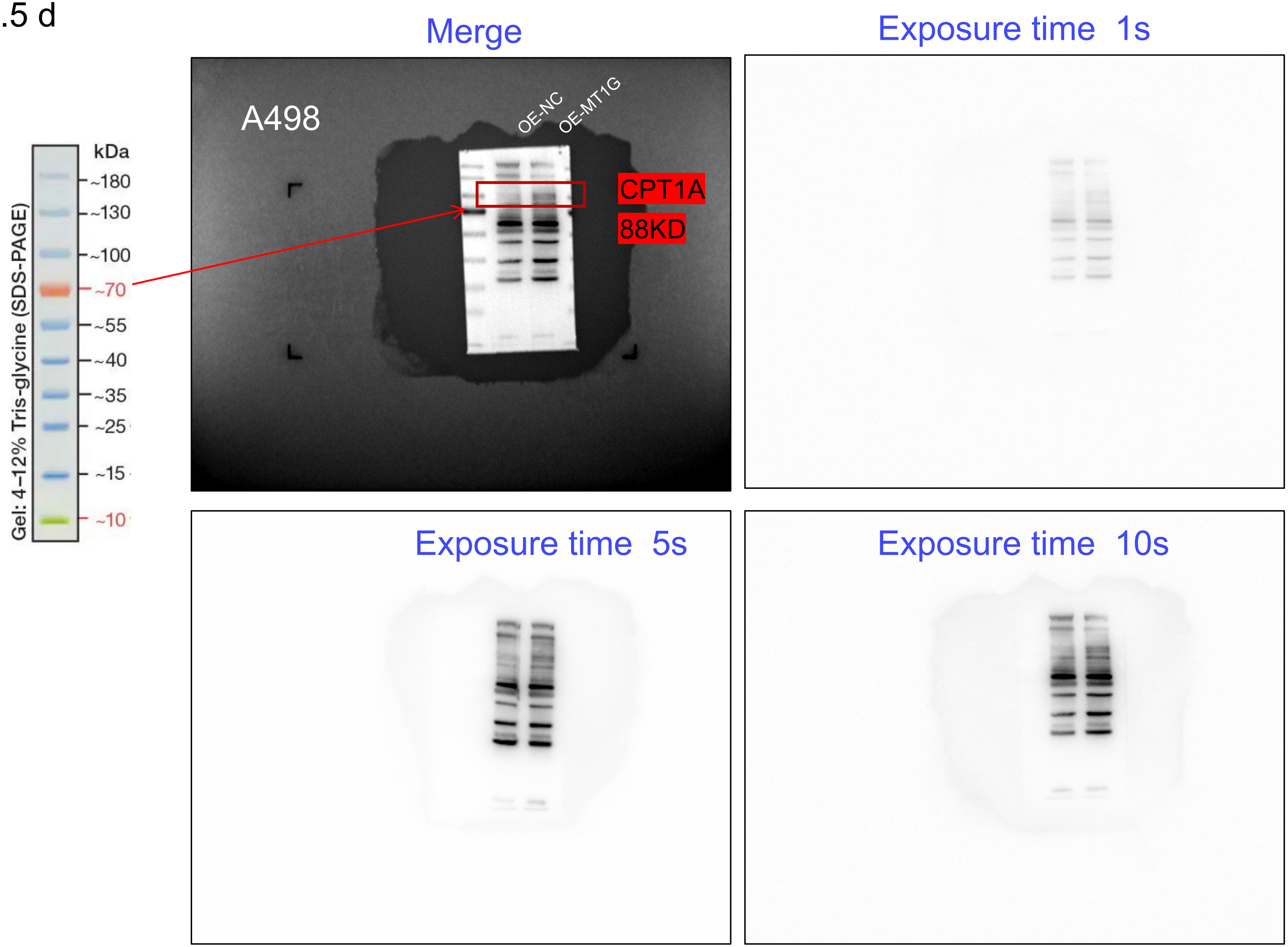
Merge
Exposure time 1s
Exposure time 5s
Exposure time 10s
A498
OE-NC
OE-MT1G
CPT1A
88KD
kDa
~180
~130
~100
~70
~55
~40
~35
~25
~15
~10
Gel: 4–12% Tris-glycine (SDS-PAGE)

Fig.5 d

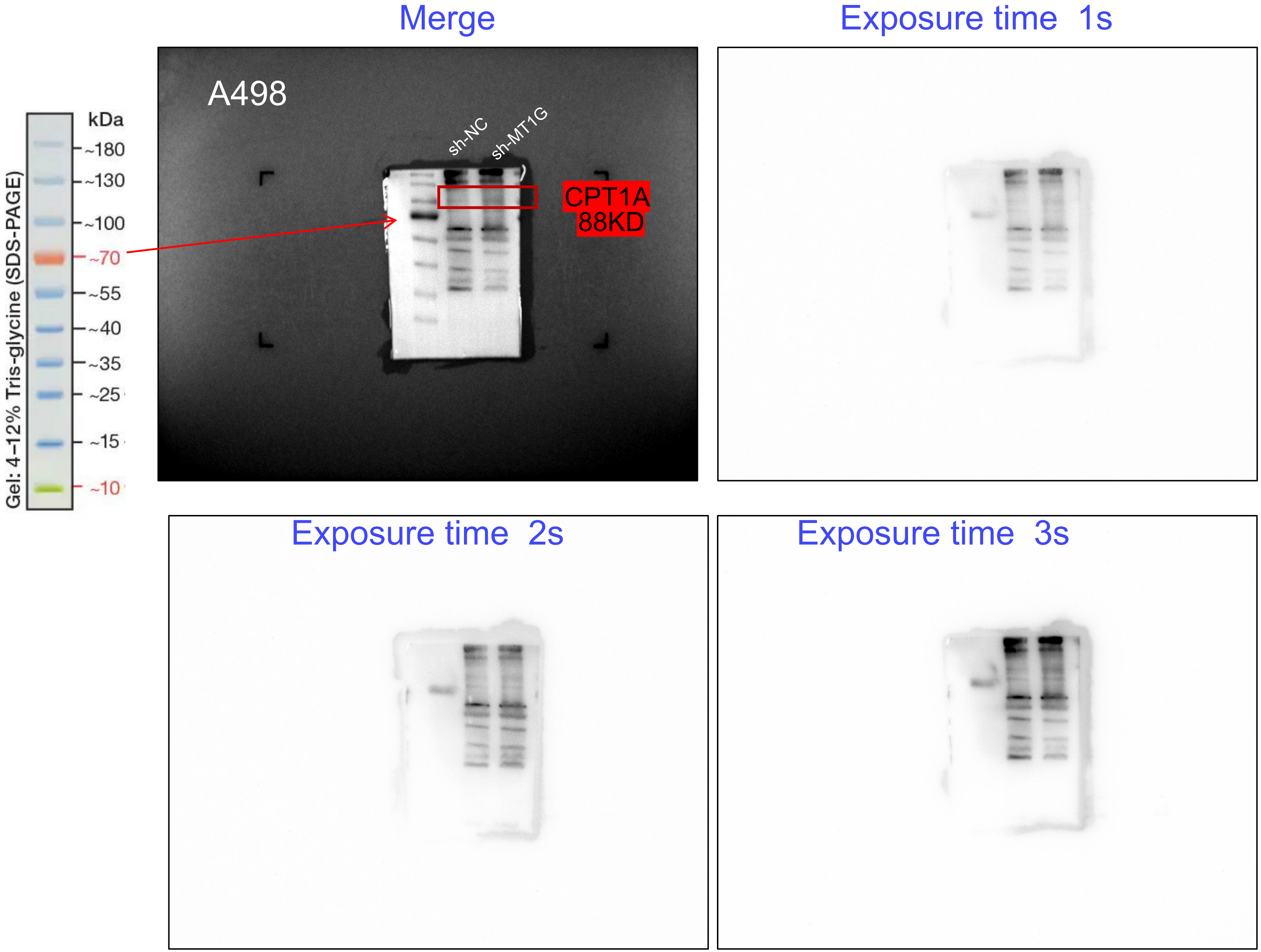
Merge
Exposure time 1s
Exposure time 2s
Exposure time 3s
A498
sh-NC
sh-MT1G
CPT1A
88KD
kDa
~180
~130
~100
~70
~55
~40
~35
~25
~15
~10
Gel: 4–12% Tris-glycine (SDS-PAGE)

Fig.5 d

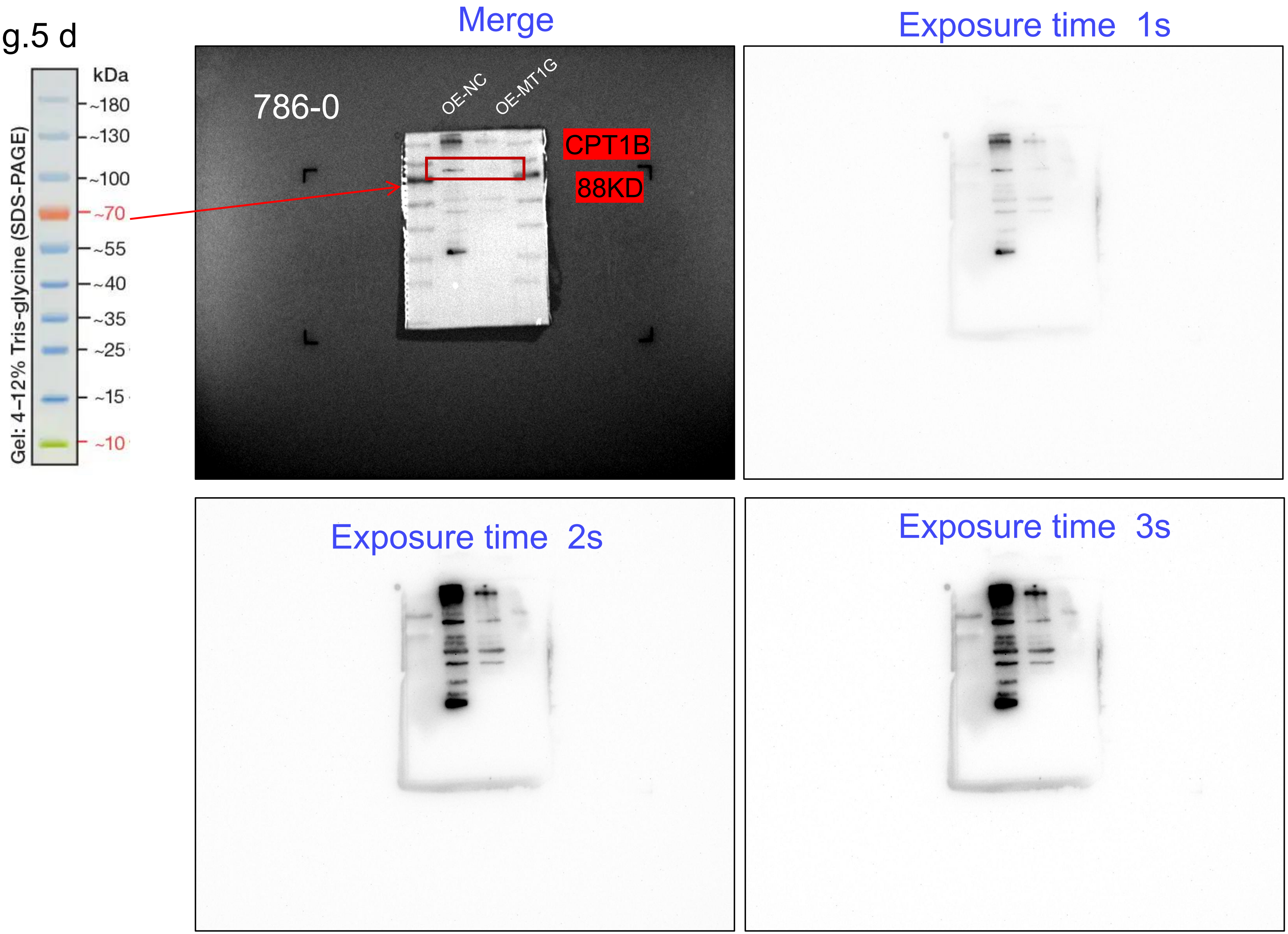

Merge
Exposure time 1s
Exposure time 2s
Exposure time 3s
kDa
~180
~130
~100
~70
~55
~40
~35
~25
~15
~10
Gel: 4–12% Tris-glycine (SDS-PAGE)
786-0
OE-NC
OE-MT1G
CPT1B
88KD

Fig.5 d

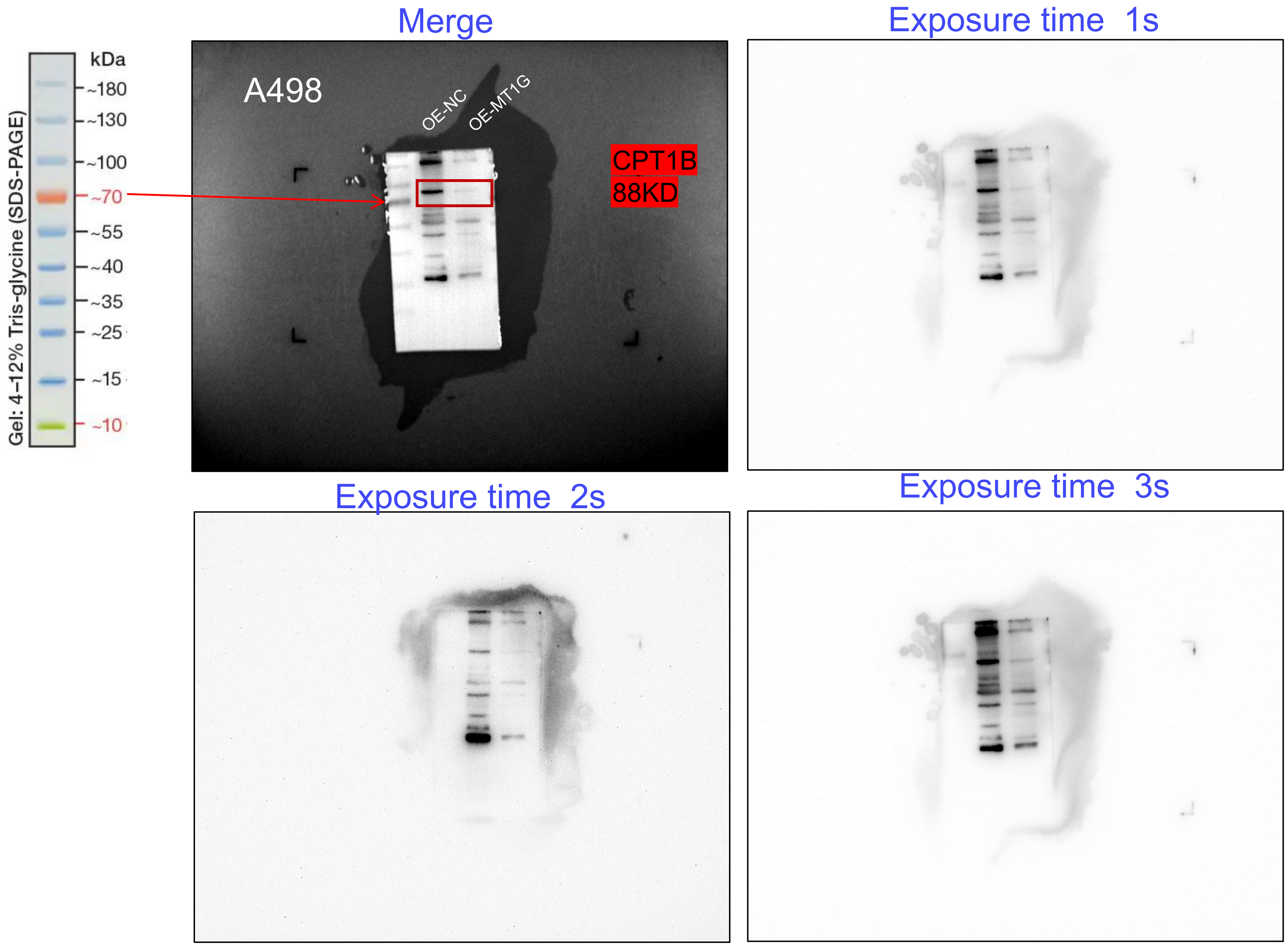

Merge
Exposure time 1s
Exposure time 2s
Exposure time 3s
kDa
~180
~130
~100
~70
~55
~40
~35
~25
~15
~10
Gel: 4–12% Tris-glycine (SDS-PAGE)
A498
OE-NC
OE-MT1G
CPT1B
88KD

Fig.5 d

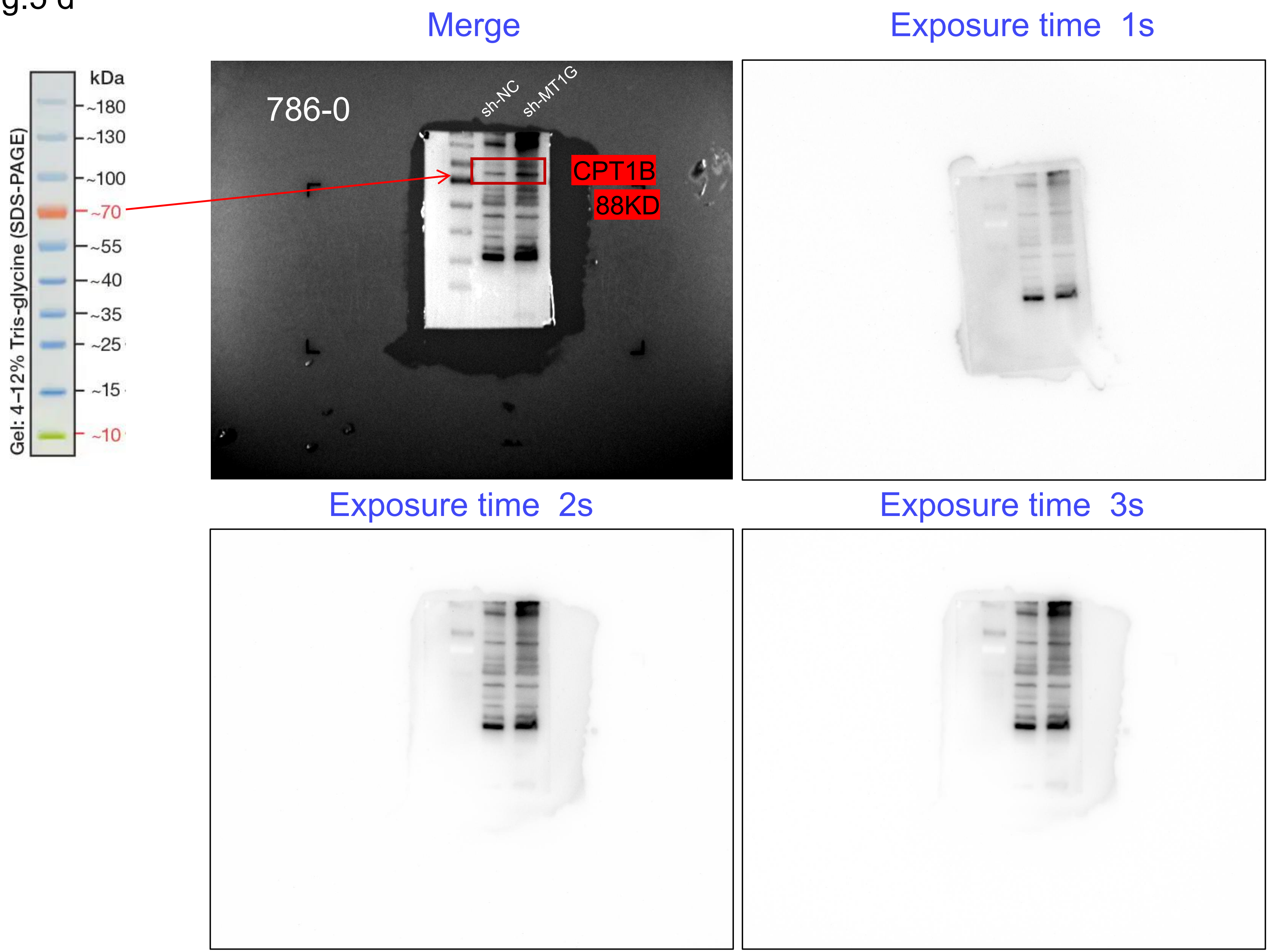

Merge
Exposure time 1s
Exposure time 2s
Exposure time 3s
kDa
~180
~130
~100
~70
~55
~40
~35
~25
~15
~10
Gel: 4–12% Tris-glycine (SDS-PAGE)
786-0
sh-NC
sh-MT1G
CPT1B
88KD

Fig.5 d

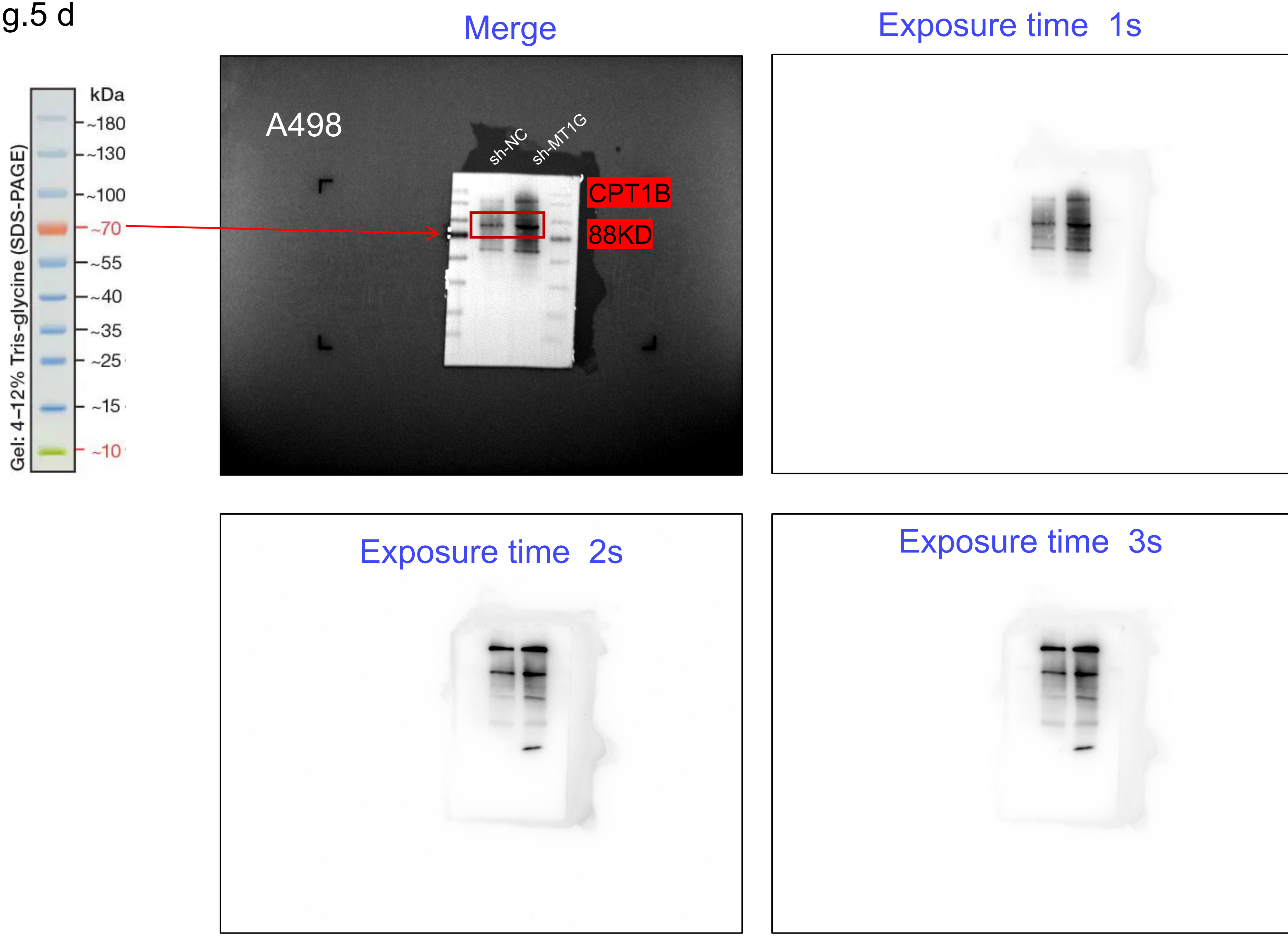

Gel: 4–12% Tris-glycine (SDS-PAGE)
kDa
~180
~130
~100
~70
~55
~40
~35
~25
~15
~10
Merge
A498
sh-NC
sh-MT1G
CPT1B
88KD
Exposure time 1s
Exposure time 2s
Exposure time 3s

Fig.5 d

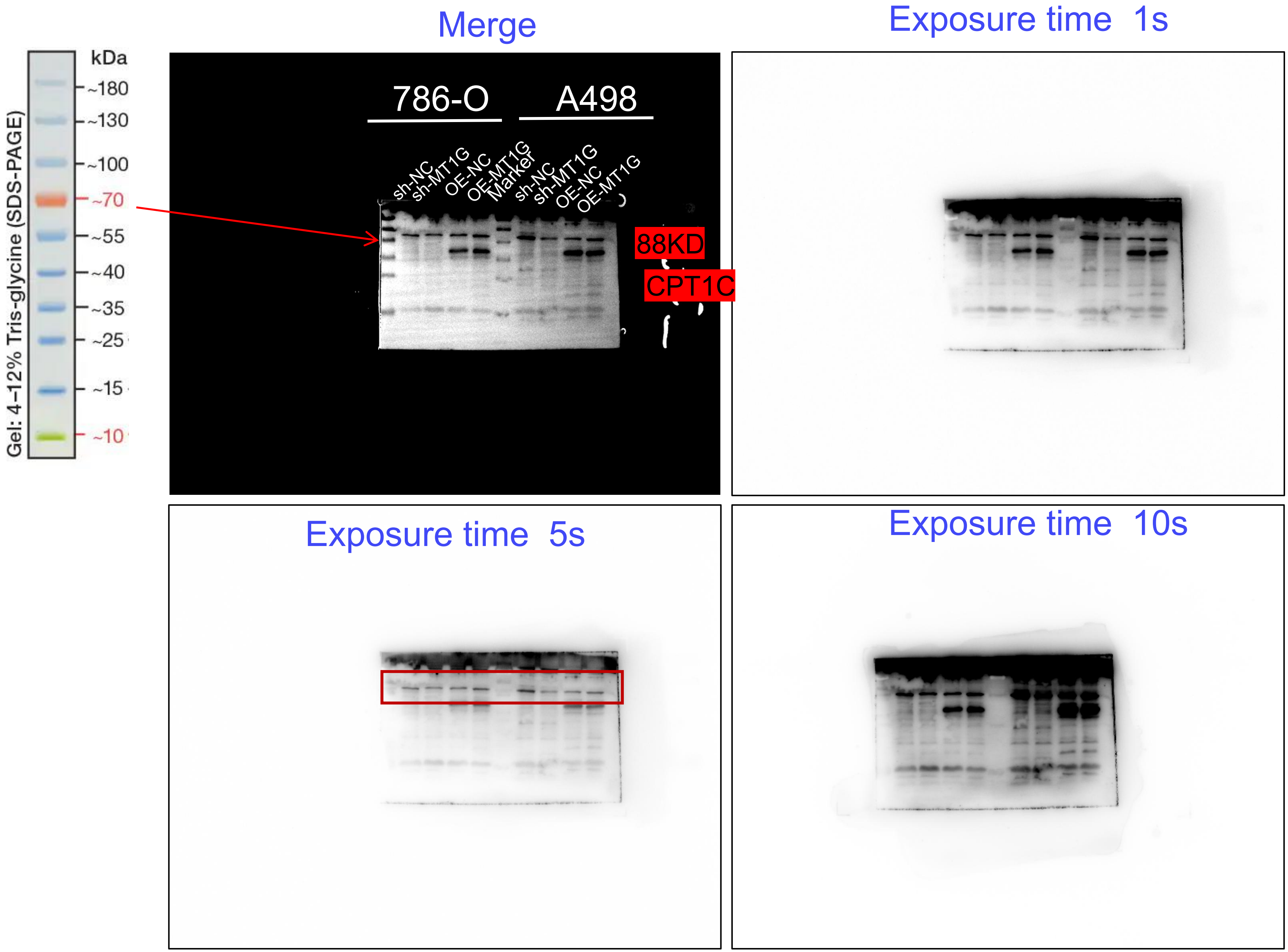
Merge
Exposure time 1s
Exposure time 5s
Exposure time 10s
kDa
~180
~130
~100
~70
~55
~40
~35
~25
~15
~10
Gel: 4–12% Tris-glycine (SDS-PAGE)
786-O
A498
sh-NC
sh-MT1G
OE-NC
OE-MT1G
Marker
sh-NC
sh-MT1G
OE-NC
OE-MT1G
88KD
CPT1C

Fig.5 d

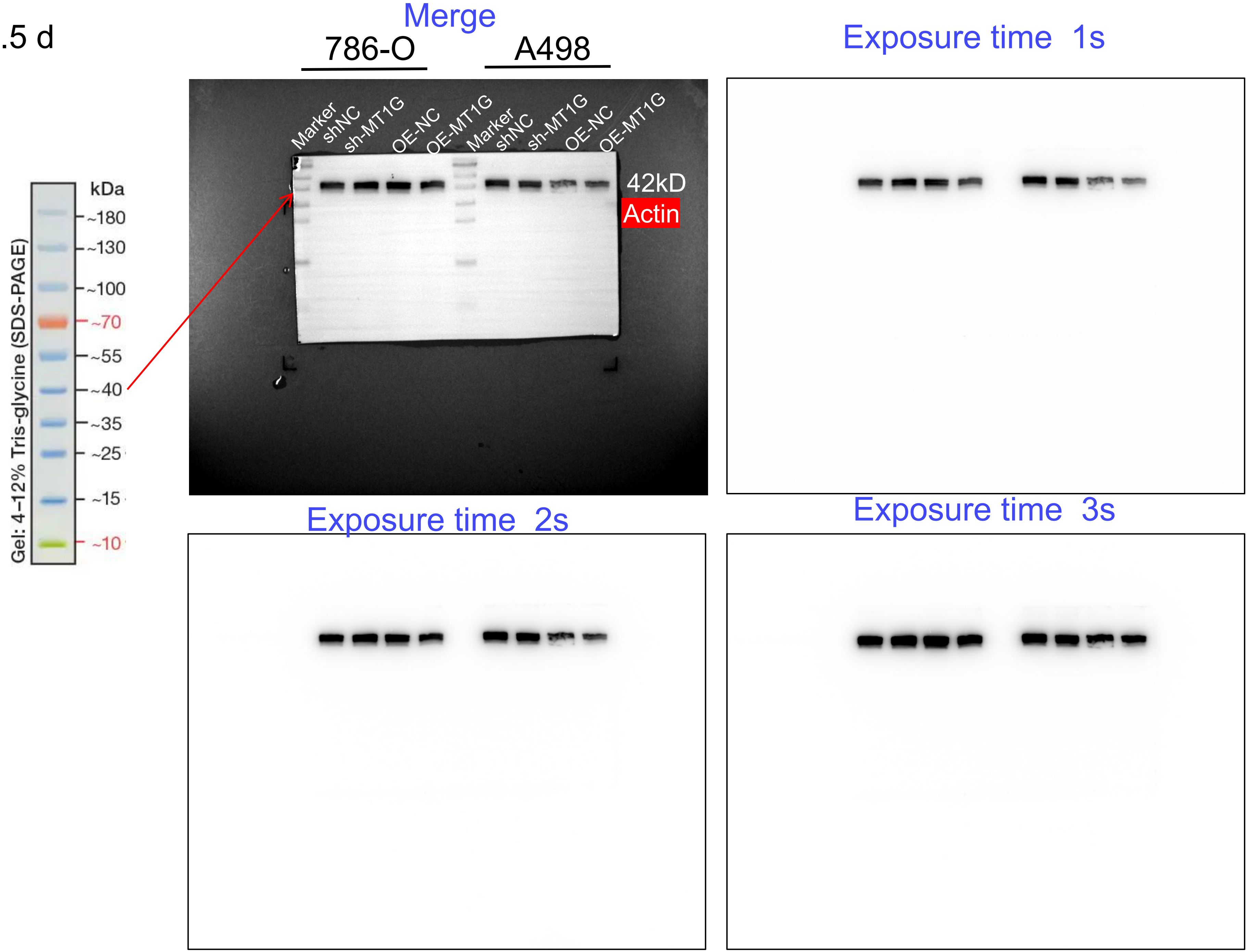

Merge
786-O
A498
Marker
shNC
sh-MT1G
OE-NC
OE-MT1G
Marker
shNC
sh-MT1G
OE-NC
OE-MT1G
42kD
Actin
kDa
~180
~130
~100
~70
~55
~40
~35
~25
~15
~10
Gel: 4–12% Tris-glycine (SDS-PAGE)
Exposure time 1s
Exposure time 2s
Exposure time 3s

Fig.6g

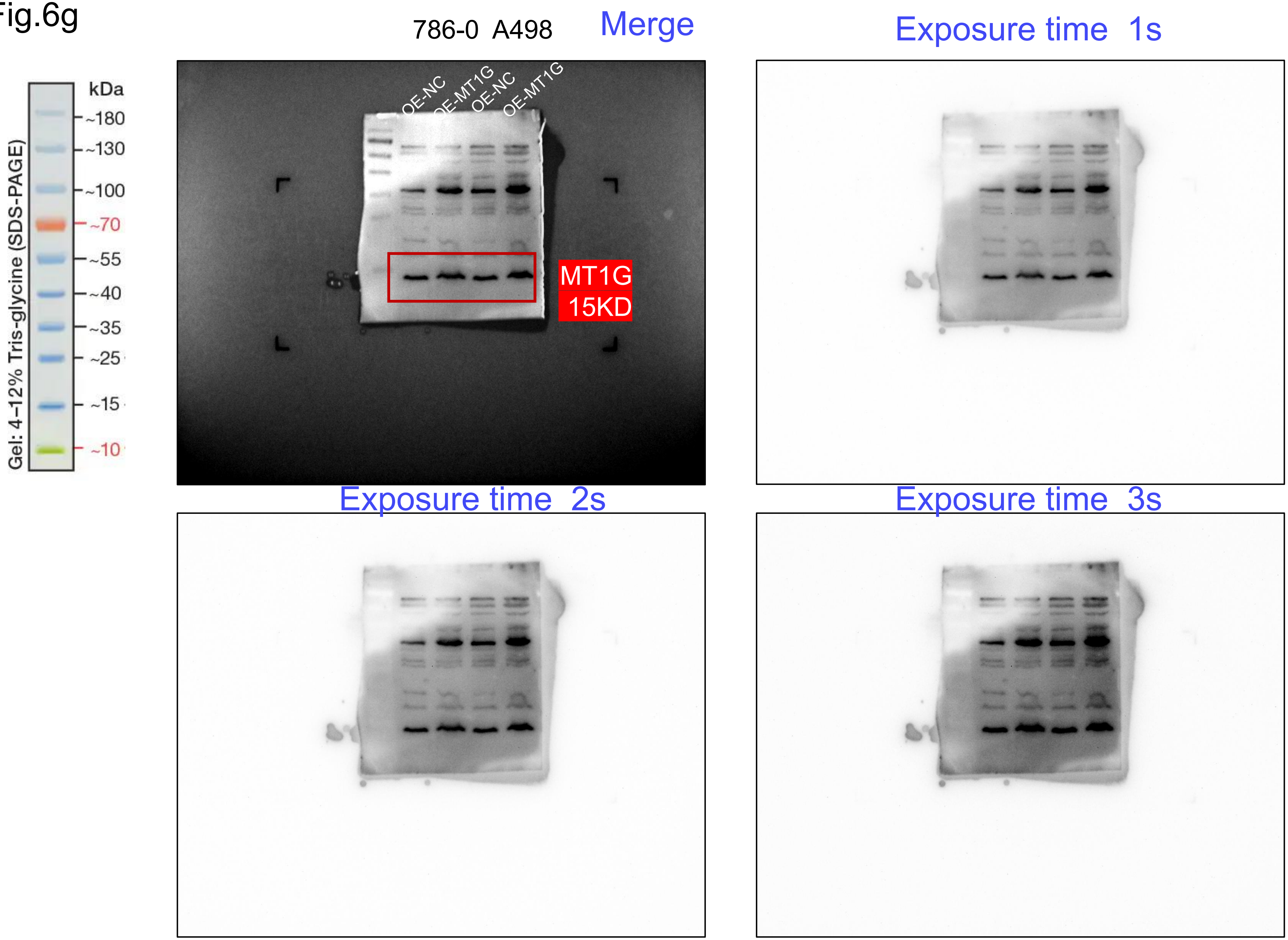

786-0 A498
Merge
Exposure time 1s
Exposure time 2s
Exposure time 3s
kDa
~180
~130
~100
~70
~55
~40
~35
~25
~15
~10
Gel: 4–12% Tris-glycine (SDS-PAGE)
OE-NC
OE-MT1G
OE-NC
OE-MT1G
MT1G
15KD

Fig.6g

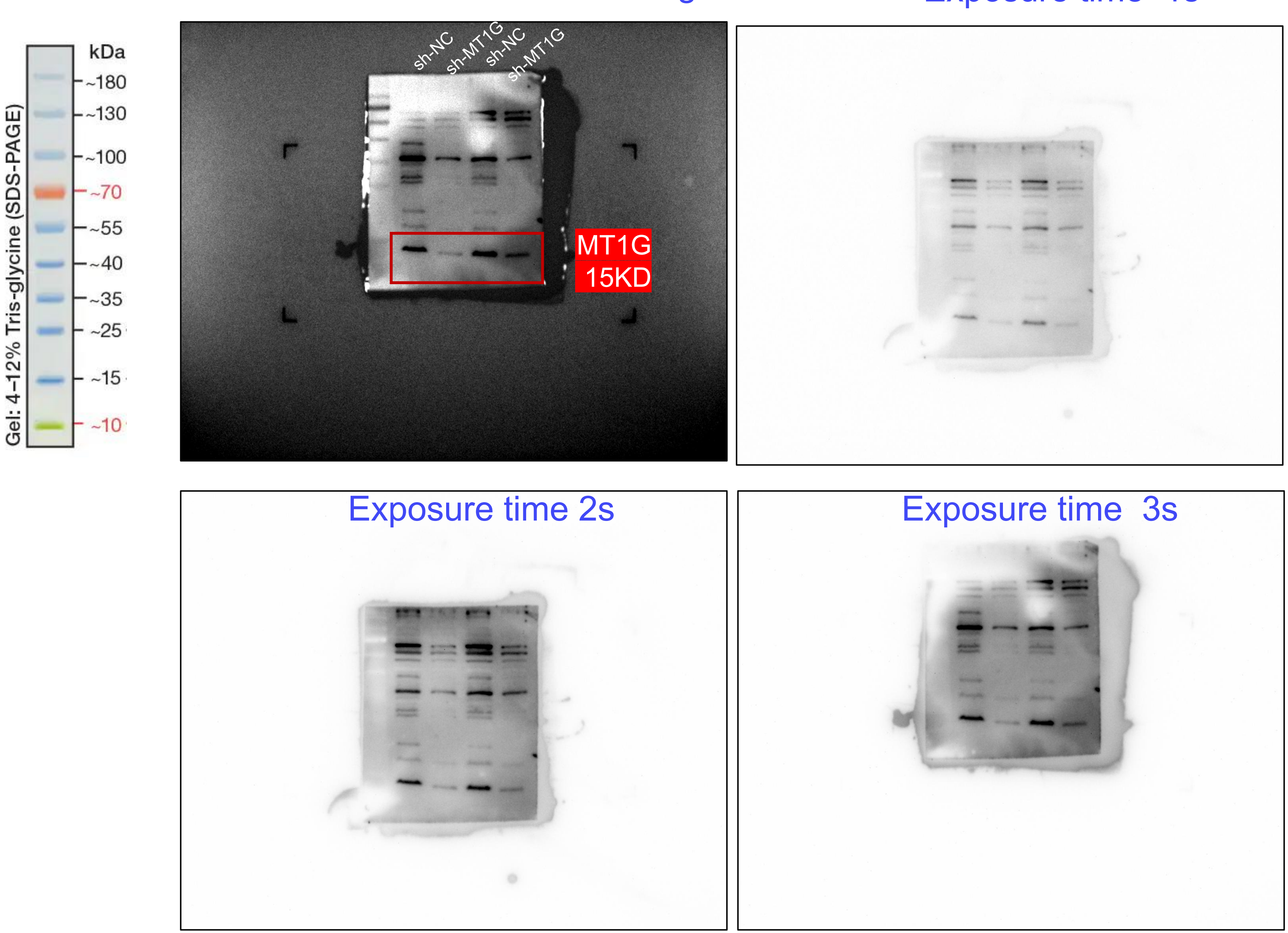

Gel: 4–12% Tris-glycine (SDS-PAGE)
kDa
~180
~130
~100
~70
~55
~40
~35
~25
~15
~10
786-0 A498
Merge
sh-NC
sh-MT1G
sh-NC
sh-MT1G
MT1G
15KD
Exposure time 1s
Exposure time 2s
Exposure time 3s

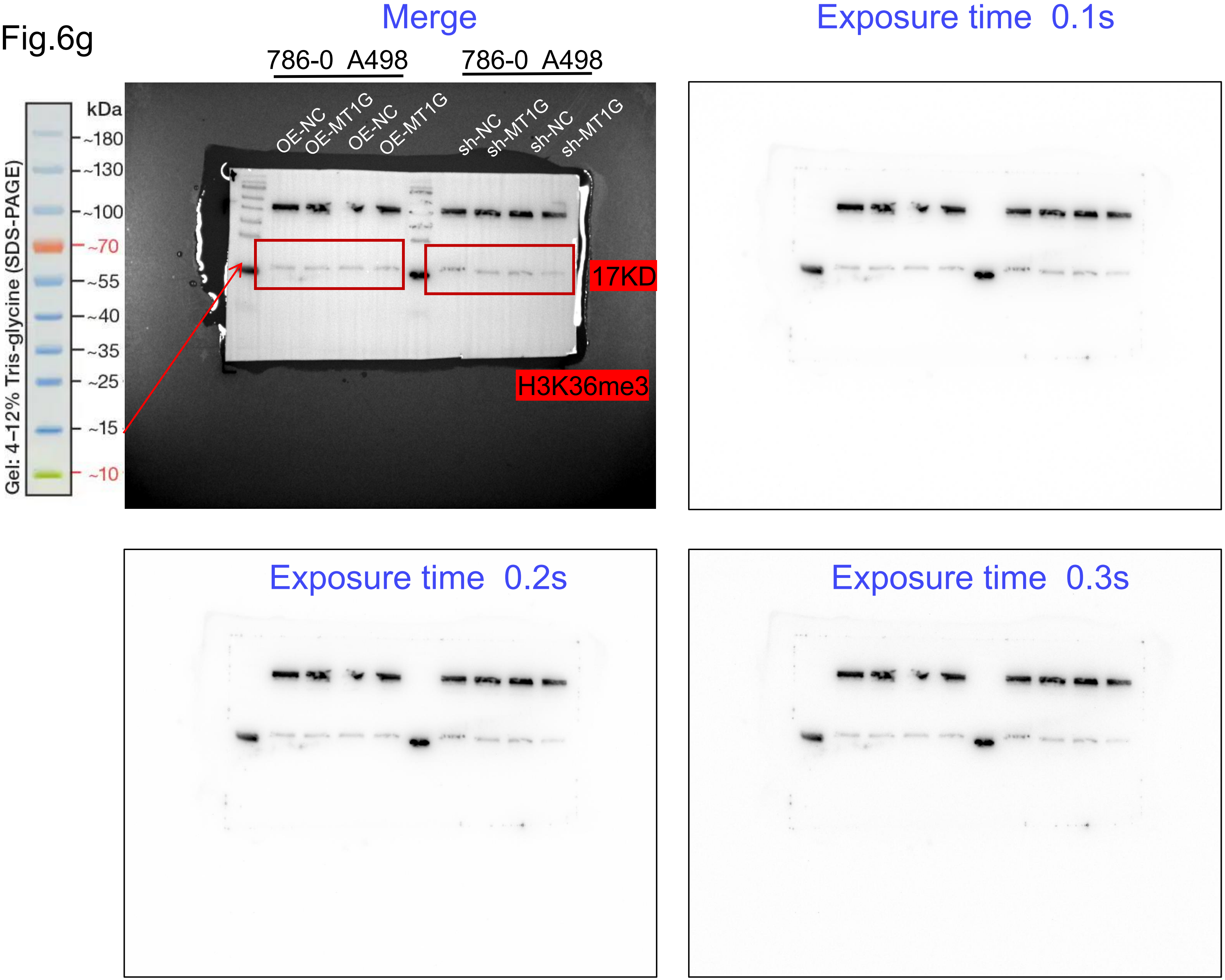
Merge
Exposure time 0.1s
786-0 A498
786-0 A498
kDa
~180
~130
~100
~70
~55
~40
~35
~25
~15
~10
Gel: 4–12% Tris-glycine (SDS-PAGE)
OE-NC
OE-MT1G
OE-NC
OE-MT1G
sh-NC
sh-MT1G
sh-NC
sh-MT1G
17KD
H3K36me3
Exposure time 0.2s
Exposure time 0.3s

Fig.6g

Fig.6g

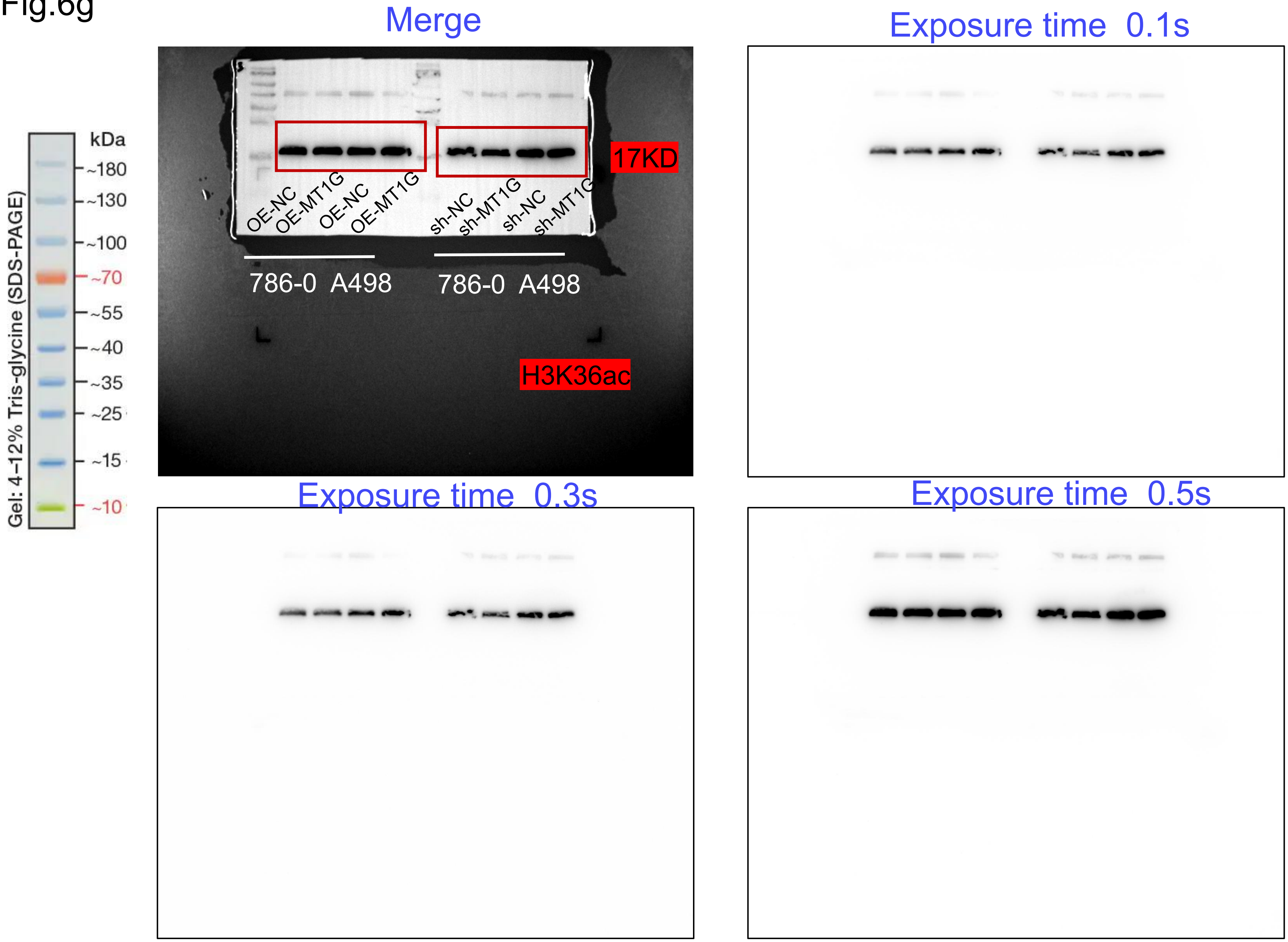
Merge
Exposure time 0.1s
Exposure time 0.3s
Exposure time 0.5s
Gel: 4–12% Tris-glycine (SDS-PAGE)
kDa
~180
~130
~100
~70
~55
~40
~35
~25
~15
~10
17KD
OE-NC
OE-MT1G
OE-NC
OE-MT1G
sh-NC
sh-MT1G
sh-NC
sh-MT1G
786-0 A498
786-0 A498
H3K36ac

Fig.6g

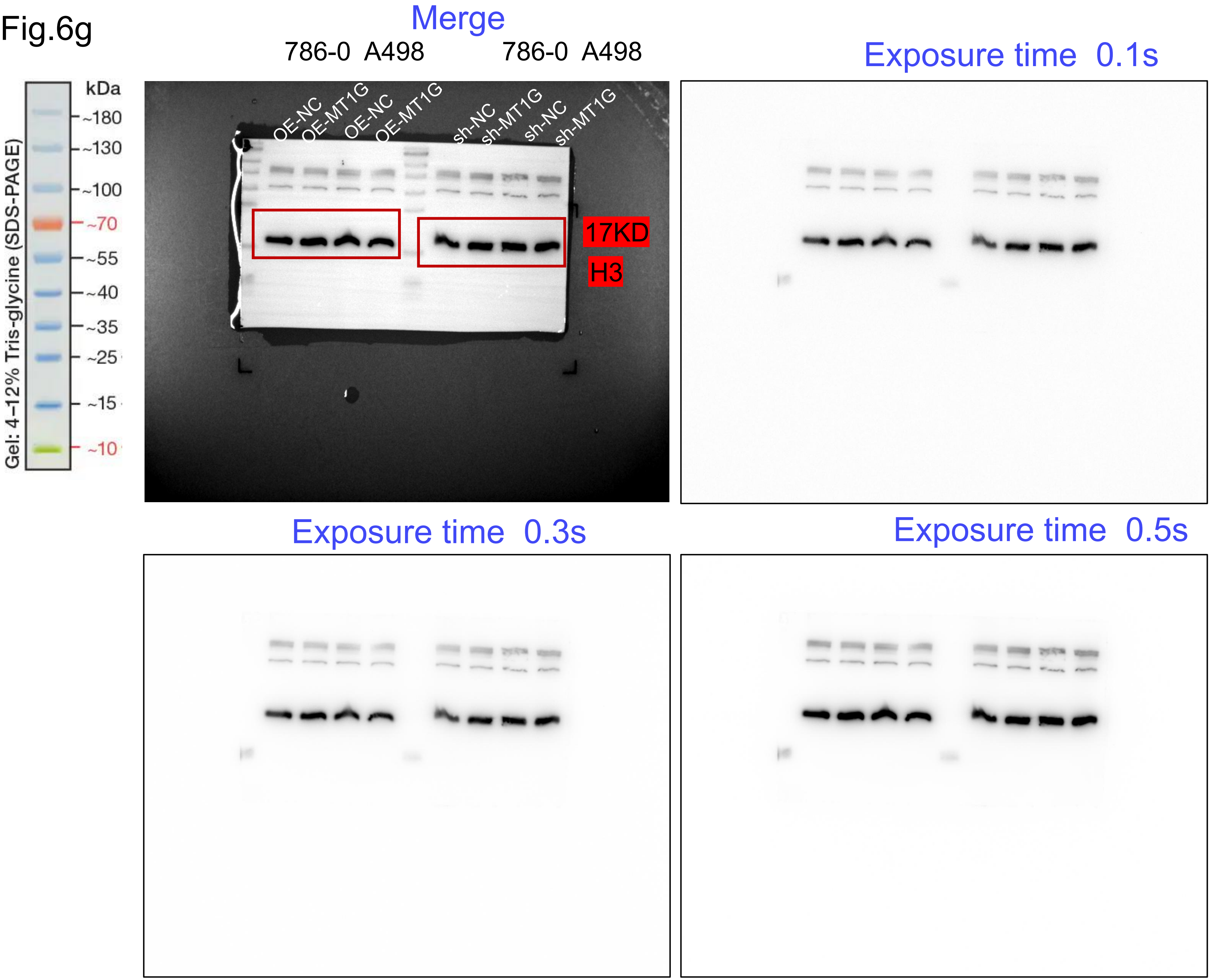

Merge
786-0 A498
786-0 A498
OE-NC
OE-MT1G
OE-NC
OE-MT1G
sh-NC
sh-MT1G
sh-NC
sh-MT1G
17KD
H3
kDa
~180
~130
~100
~70
~55
~40
~35
~25
~15
~10
Gel: 4–12% Tris-glycine (SDS-PAGE)
Exposure time 0.1s
Exposure time 0.3s
Exposure time 0.5s

Fig.6g

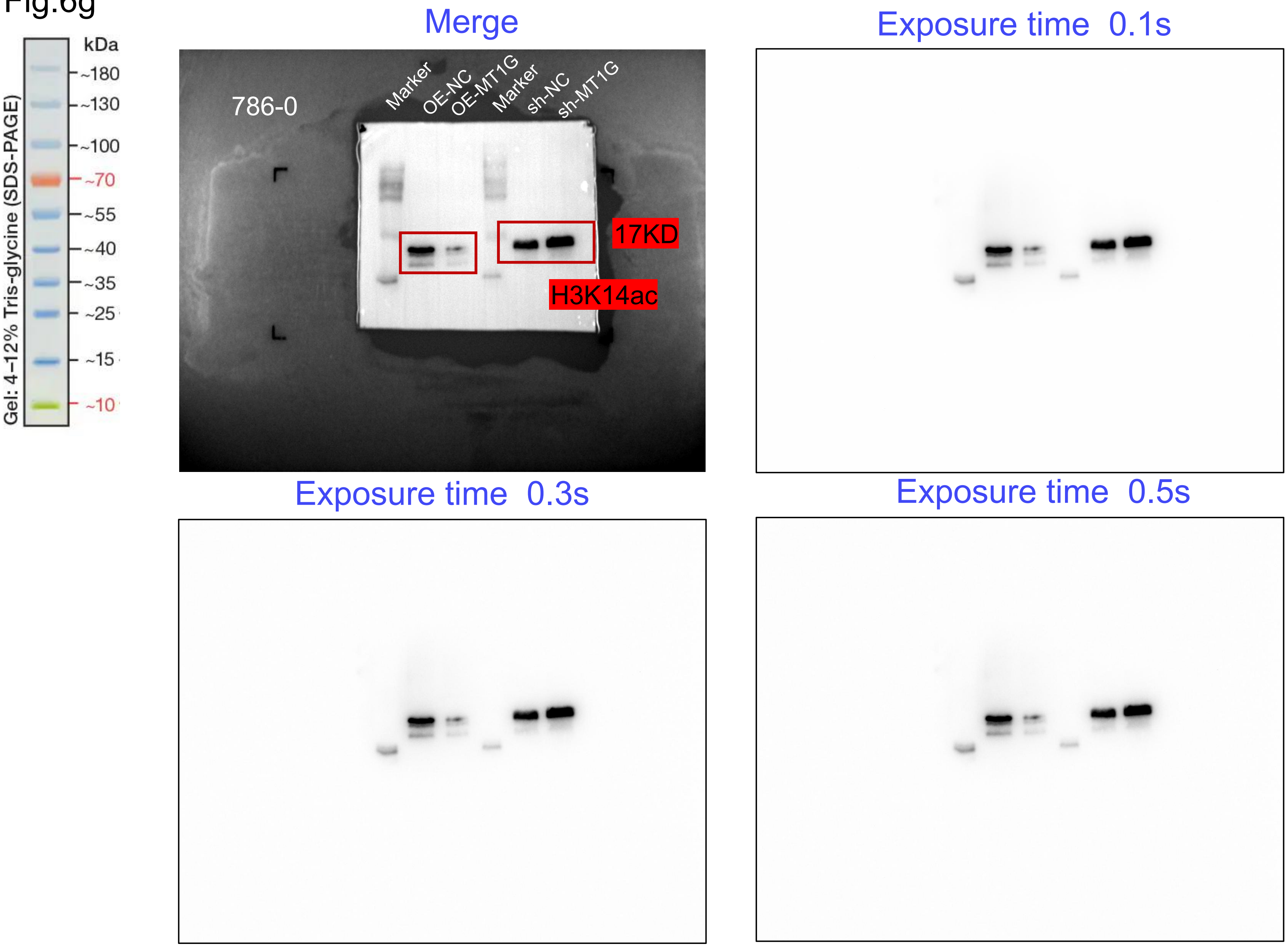
kDa
~180
~130
~100
~70
~55
~40
~35
~25
~15
~10
Gel: 4–12% Tris-glycine (SDS-PAGE)
Merge
786-0
Marker
OE-NC
OE-MT1G
Marker
sh-NC
sh-MT1G
17KD
H3K14ac
Exposure time 0.1s
Exposure time 0.3s
Exposure time 0.5s

Fig.6g

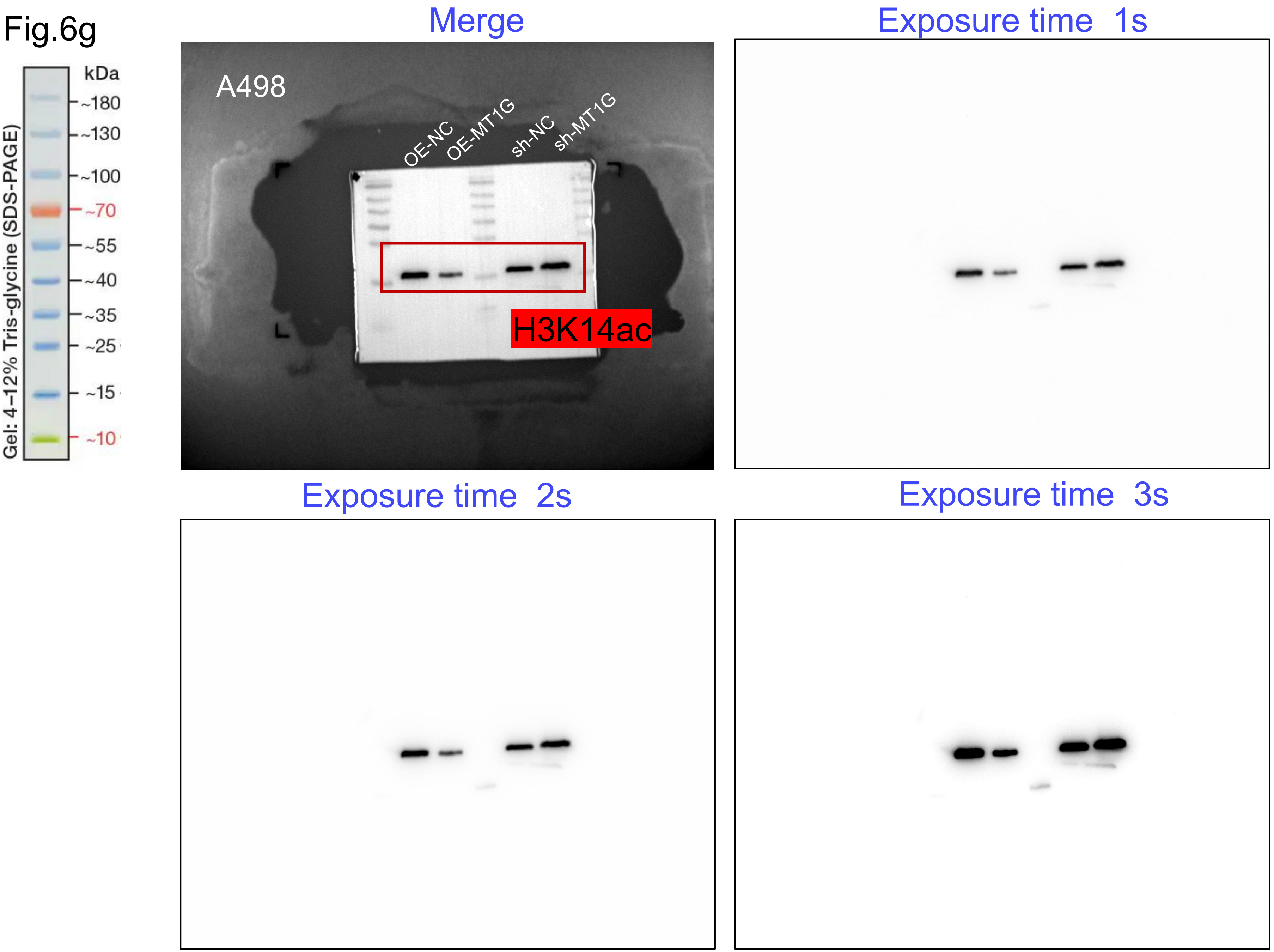

Merge
Exposure time 1s
Exposure time 2s
Exposure time 3s
kDa
~180
~130
~100
~70
~55
~40
~35
~25
~15
~10
Gel: 4–12% Tris-glycine (SDS-PAGE)
A498
OE-NC
OE-MT1G
sh-NC
sh-MT1G
H3K14ac

Fig.6g

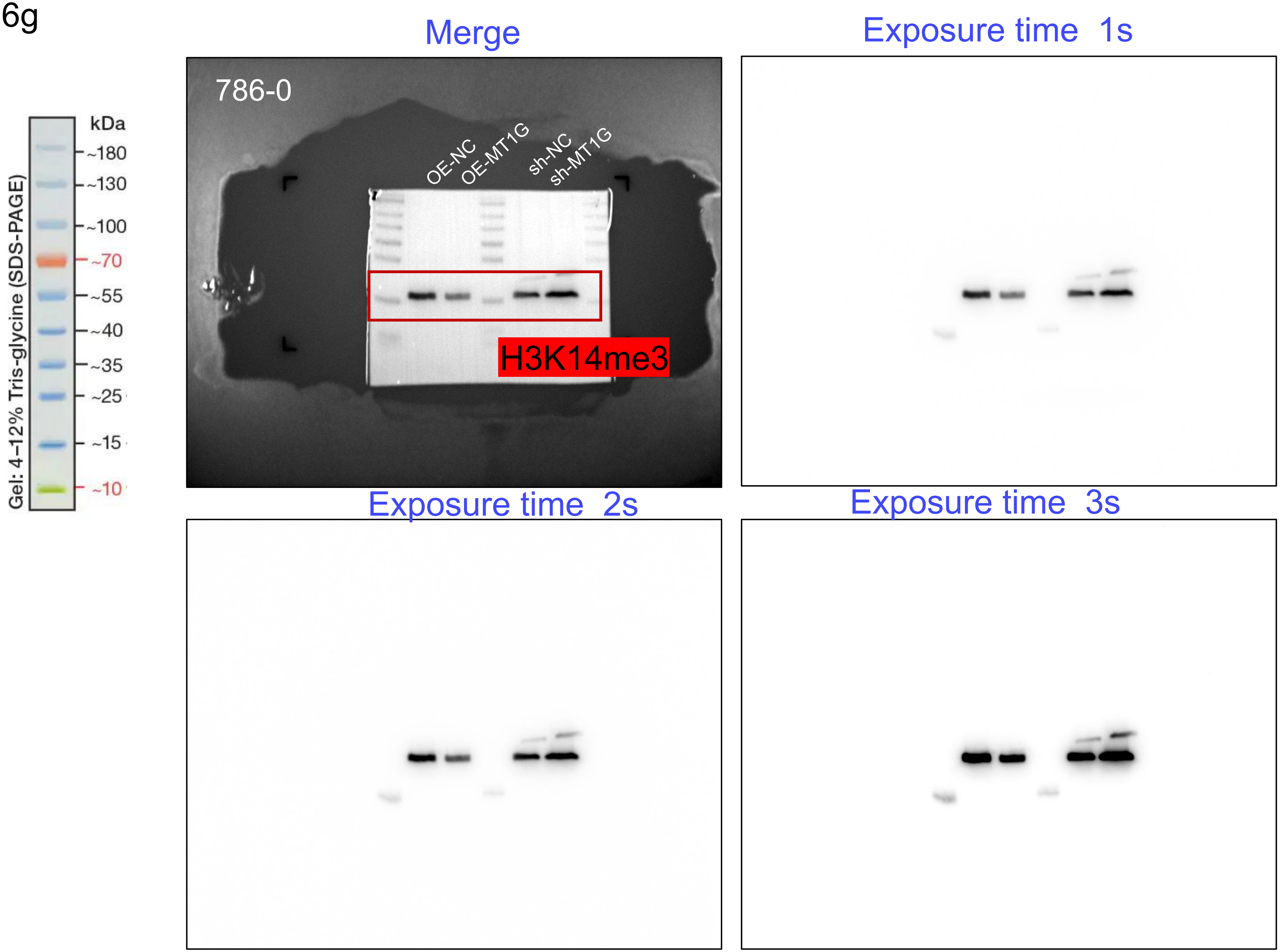
Gel: 4–12% Tris-glycine (SDS-PAGE)
kDa
~180
~130
~100
~70
~55
~40
~35
~25
~15
~10
Merge
786-0
OE-NC
OE-MT1G
sh-NC
sh-MT1G
H3K14me3
Exposure time 1s
Exposure time 2s
Exposure time 3s

Fig.6g

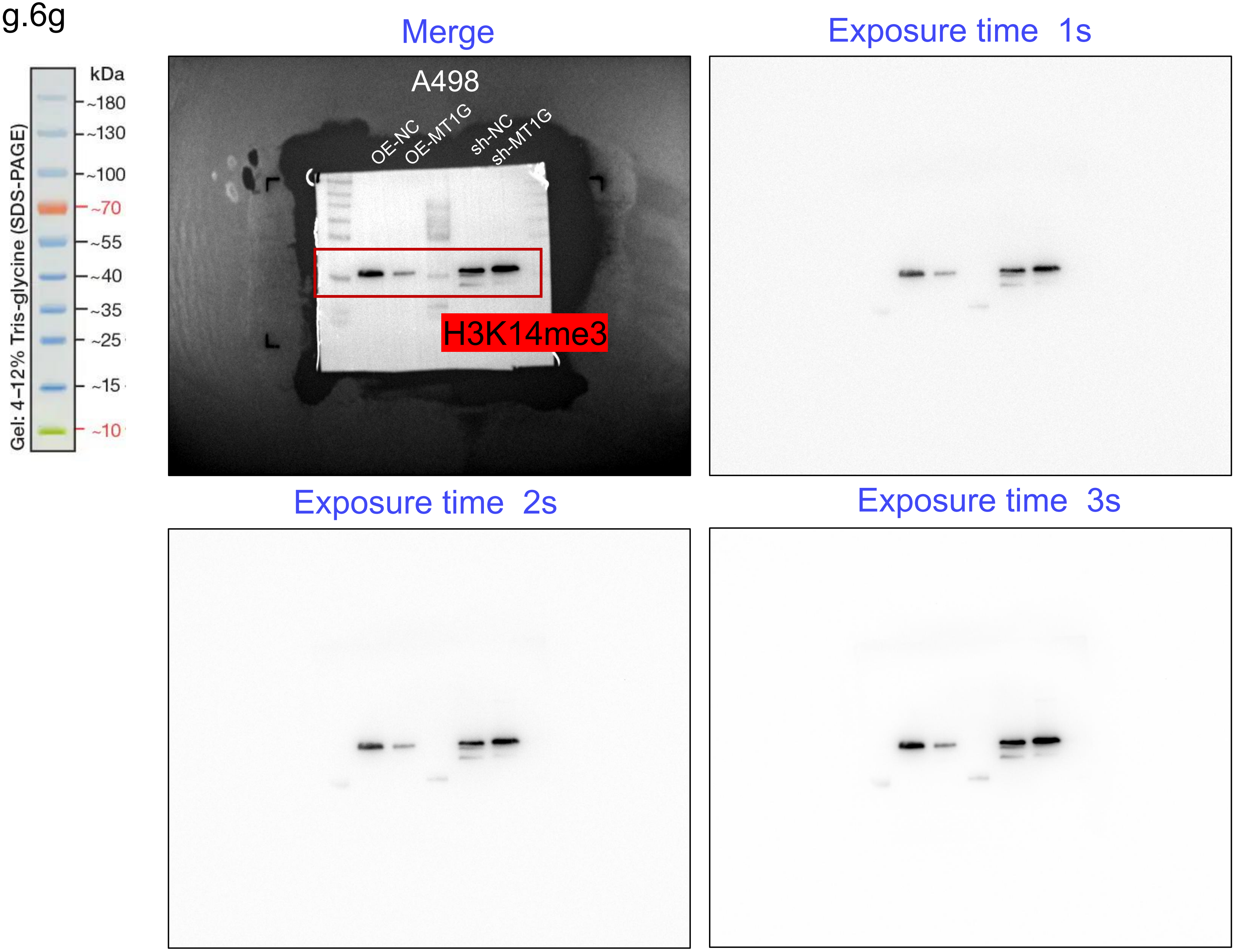
kDa
~180
~130
~100
~70
~55
~40
~35
~25
~15
~10
Gel: 4–12% Tris-glycine (SDS-PAGE)
Merge
A498
OE-NC
OE-MT1G
sh-NC
sh-MT1G
H3K14me3
Exposure time 1s
Exposure time 2s
Exposure time 3s

Fig.6g

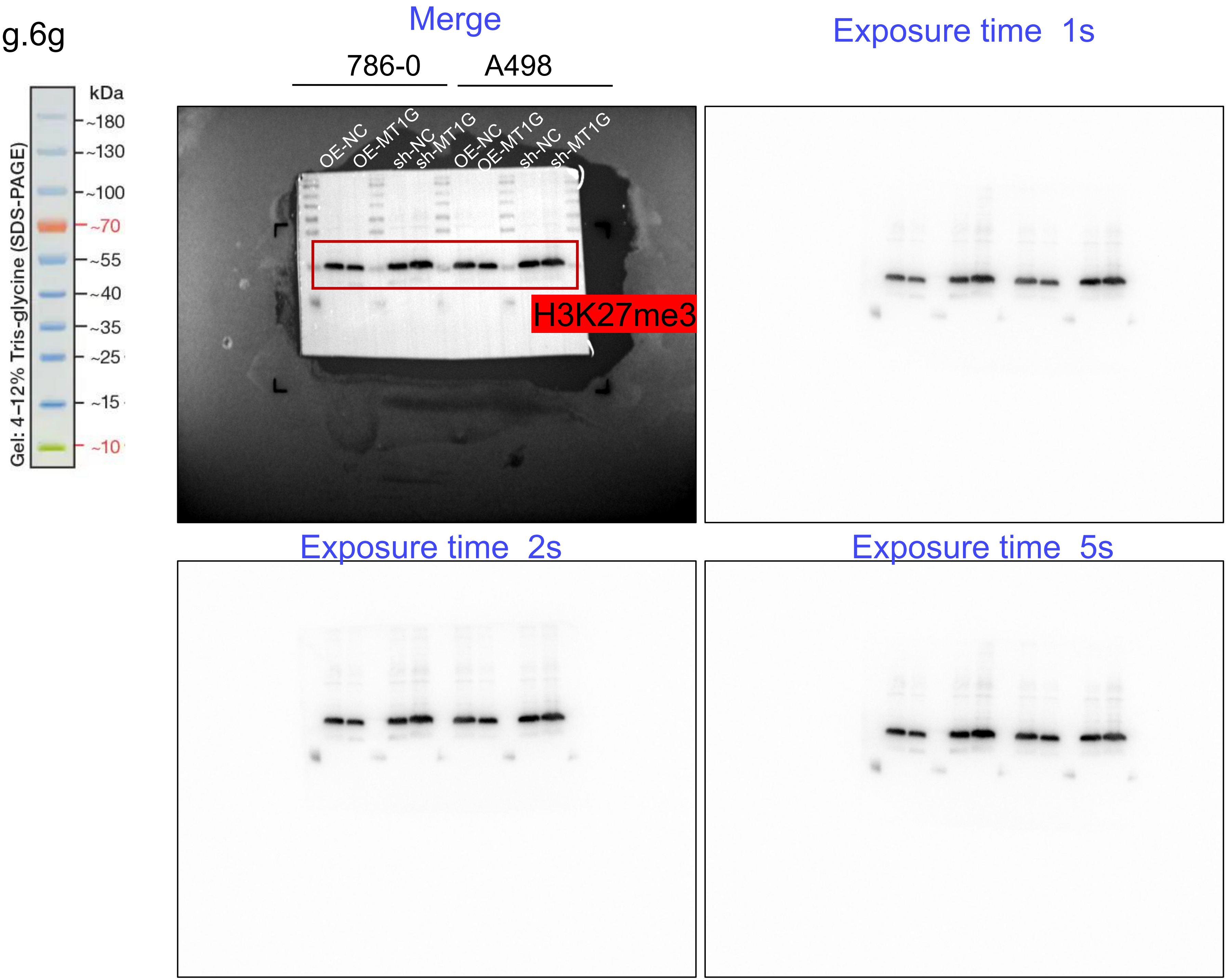
Merge
786-0
A498
kDa
~180
~130
~100
~70
~55
~40
~35
~25
~15
~10
Gel: 4–12% Tris-glycine (SDS-PAGE)
OE-NC
OE-MT1G
sh-NC
sh-MT1G
OE-NC
OE-MT1G
sh-NC
sh-MT1G
H3K27me3
Exposure time 1s
Exposure time 2s
Exposure time 5s

Fig.6g

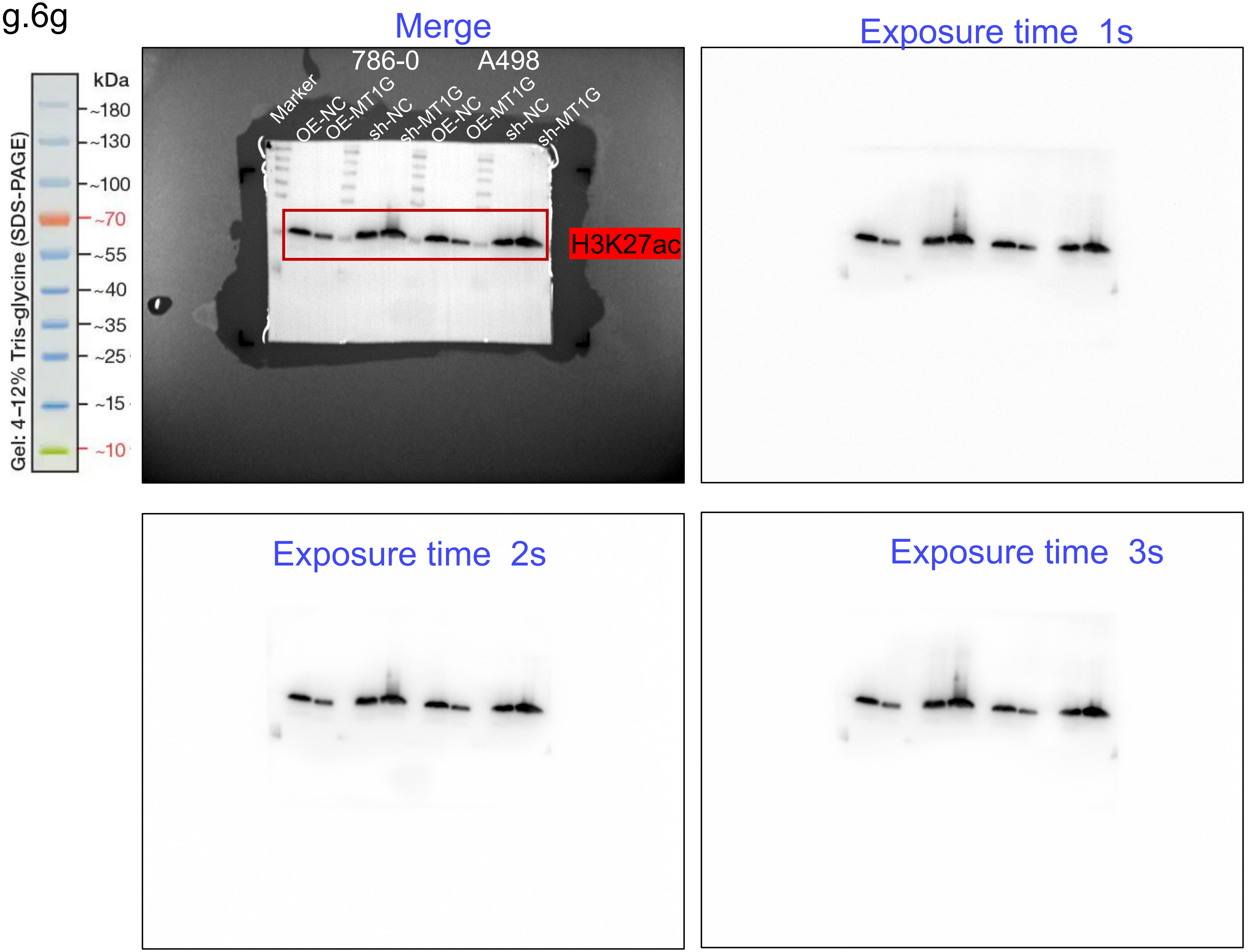
Merge
Exposure time 1s
Exposure time 2s
Exposure time 3s
kDa
~180
~130
~100
~70
~55
~40
~35
~25
~15
~10
Gel: 4–12% Tris-glycine (SDS-PAGE)
786-0
A498
Marker
OE-NC
OE-MT1G
sh-NC
sh-MT1G
OE-NC
OE-MT1G
sh-NC
sh-MT1G
H3K27ac

Fig.6g

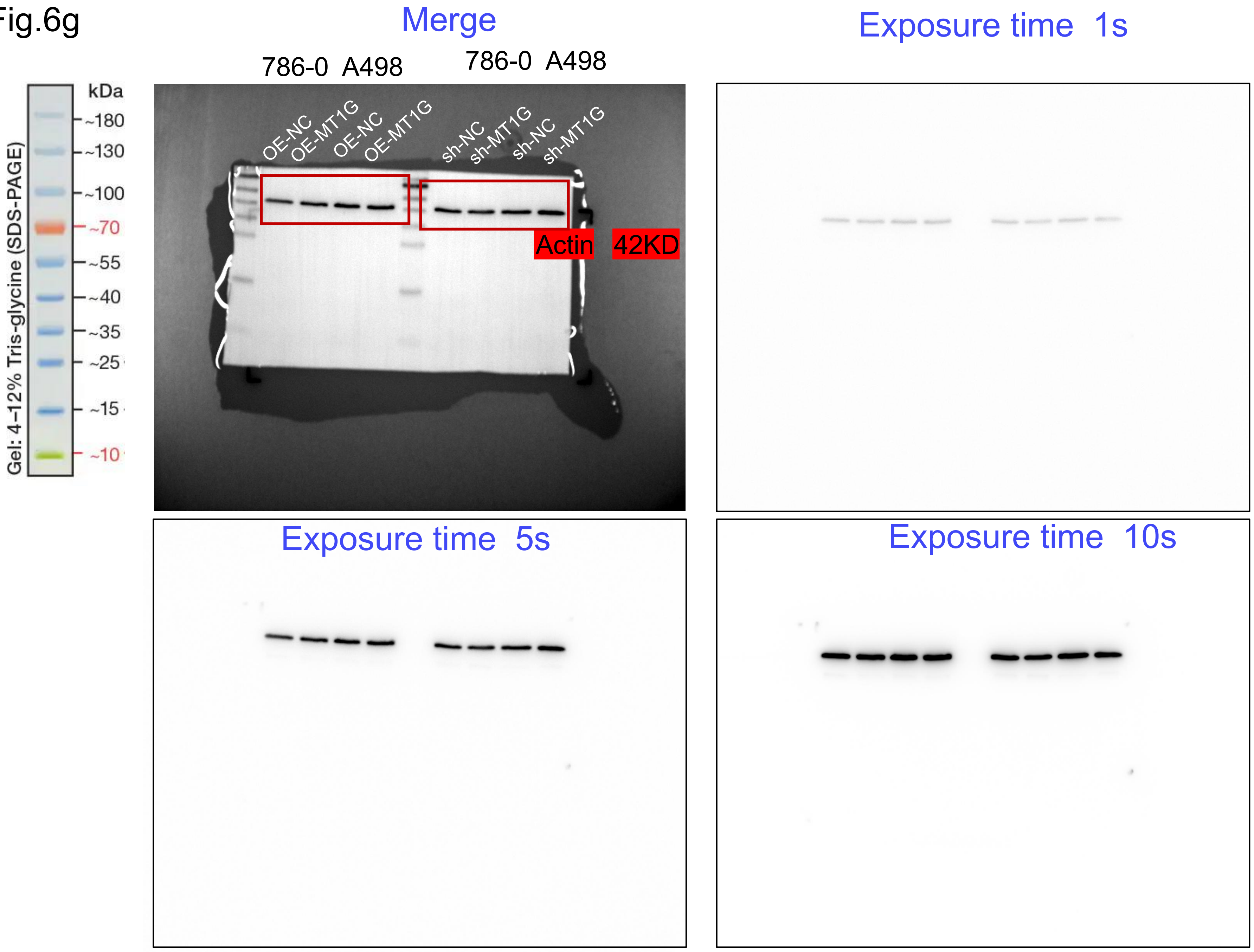

Merge
Exposure time 1s
786-0 A498
786-0 A498
kDa
~180
~130
~100
~70
~55
~40
~35
~25
~15
~10
Gel: 4–12% Tris-glycine (SDS-PAGE)
OE-NC
OE-MT1G
OE-NC
OE-MT1G
sh-NC
sh-MT1G
sh-NC
sh-MT1G
Actin
42KD
Exposure time 5s
Exposure time 10s

Supplementary Fig.8a

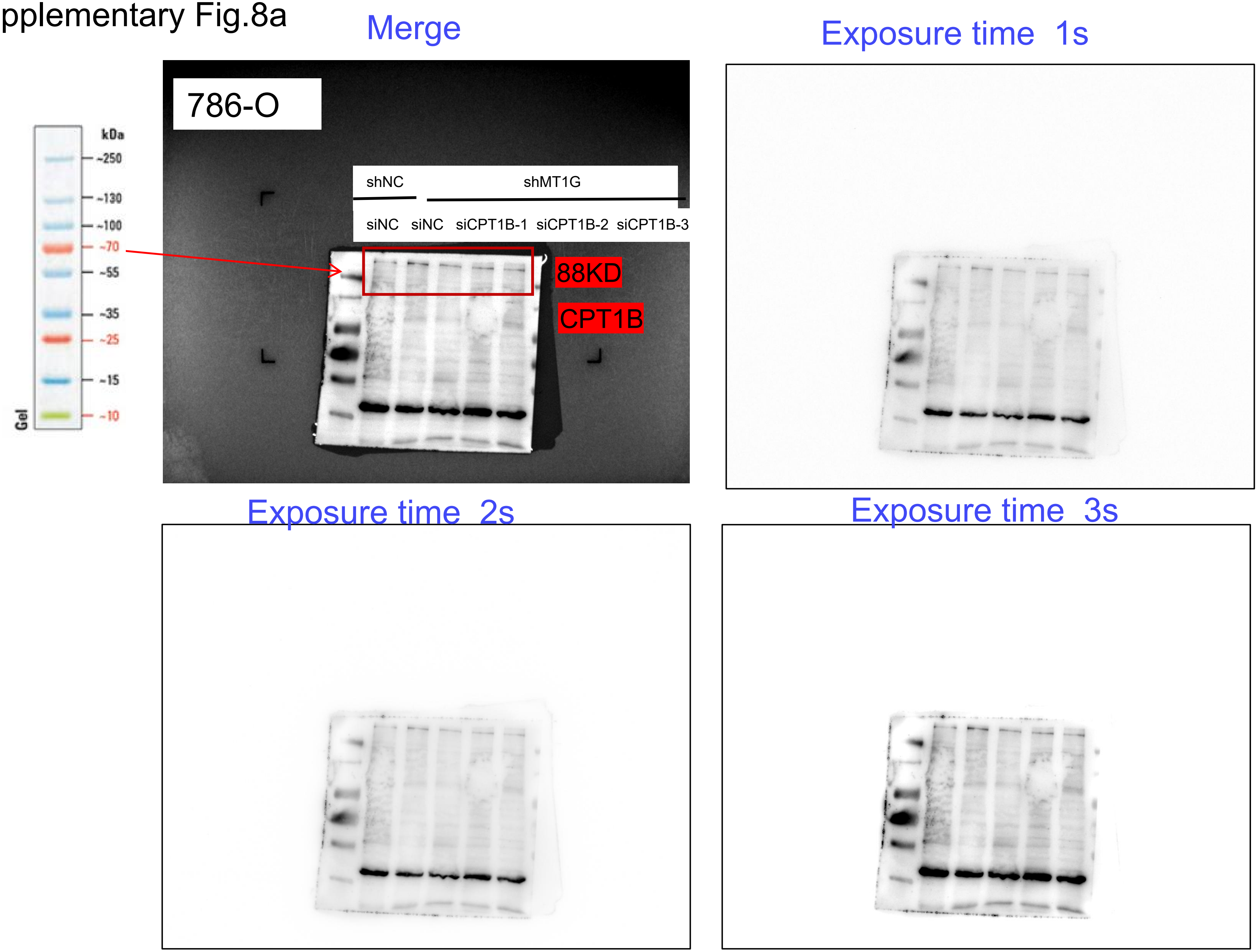

Supplementary Fig.8a

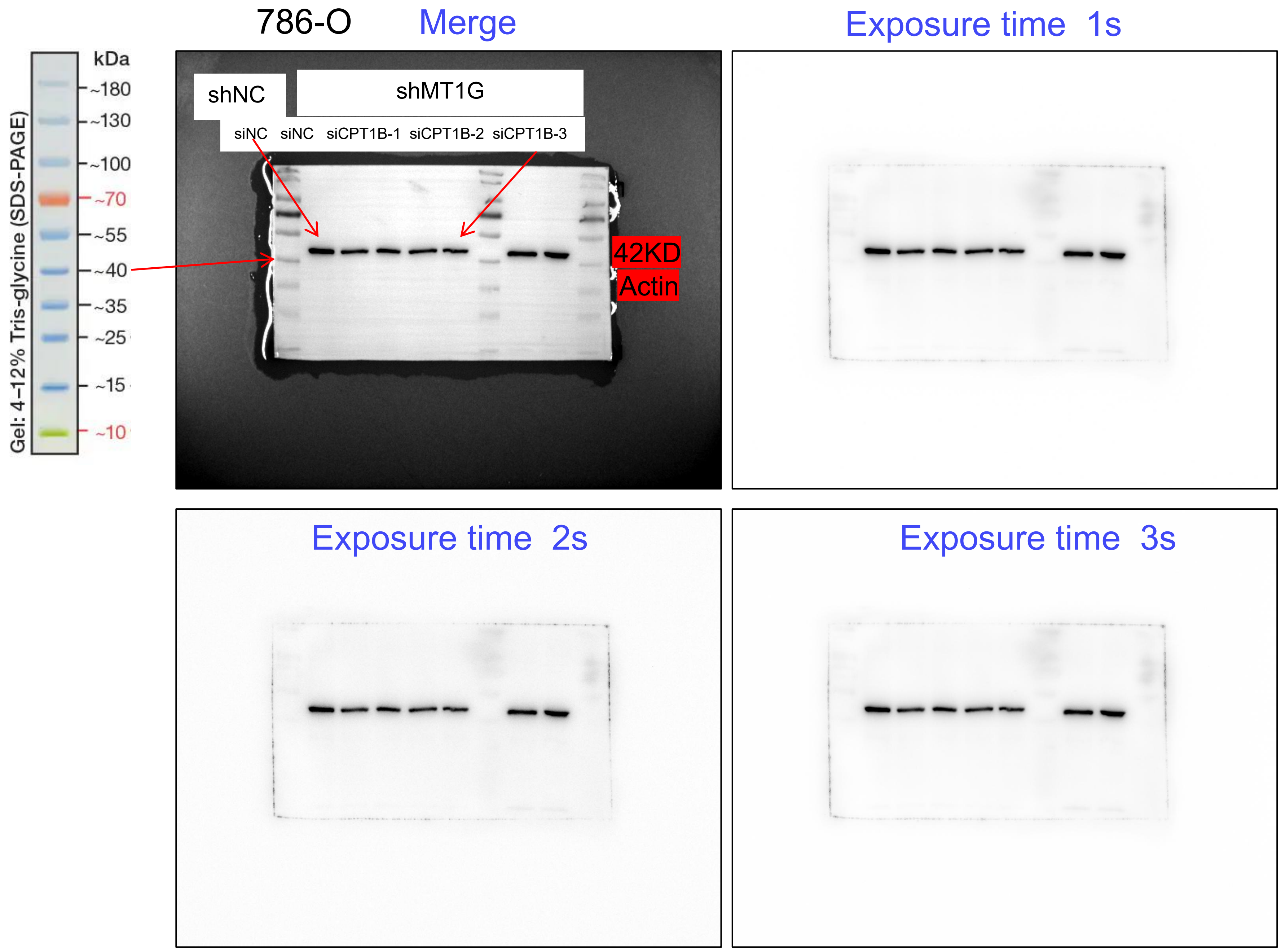

Supplement: Supplementary file 2 — WB uncropped figures 24.5.21 [file 41416_2024_2747_MOESM2_ESM.pdf]
